# Activity and Selectivity of Novel Chemical Metallic Complexes with Potential Anticancer Effects on Melanoma Cells

**DOI:** 10.3390/molecules28124851

**Published:** 2023-06-19

**Authors:** Maria Camilla Ciardulli, Annaluisa Mariconda, Marco Sirignano, Erwin Pavel Lamparelli, Raffaele Longo, Pasqualina Scala, Raffaella D’Auria, Antonietta Santoro, Liberata Guadagno, Giovanna Della Porta, Pasquale Longo

**Affiliations:** 1 Department of Medicine, Surgery and Dentistry, University of Salerno, Via S. Allende, 84081 Baronissi, Italy; mciardulli@unisa.it (M.C.C.); elamparelli@unisa.it (E.P.L.); pscala@unisa.it (P.S.); radauria@unisa.it (R.D.); ansantoro@unisa.it (A.S.); 2 Department of Science, University of Basilicata, Viale dell’Ateneo Lucano 10, 85100 Potenza, Italy; annaluisa.mariconda@unibas.it; 3 Department of Chemistry and Biology “Adolfo Zambelli”, University of Salerno, Via Giovanni Paolo II, 132, 84084 Fisciano, Italy; msirignano@unisa.it (M.S.); plongo@unisa.it (P.L.); 4 Department of Industrial Engineering, University of Salerno, Via Giovanni Paolo II, 132, 84084 Fisciano, Italy; rlongo@unisa.it (R.L.); lguadagno@unisa.it (L.G.); 5 Interdepartment Centre BIONAM, University of Salerno, Via Giovanni Paolo II, 84084 Fisciano, Italy

**Keywords:** human malignant melanoma cells, immortal keratinocyte from adult human skin, N-heterocyclic carbene complexes, metallorganic anticancer agents, toxicity, selectivity, 3D culture, live cell imaging

## Abstract

Human malignant melanoma cells from lymph node metastatic site (MeWo) were selected for testing several synthesized and purified silver(I) and gold(I) complexes stabilized by unsymmetrically substituted N-heterocyclic carbene (NHC) ligands, called L20 (N-methyl, N′-[2-hydroxy ethylphenyl]imidazol-2-ylide) and M1 (4,5-dichloro, N-methyl, N′-[2-hydroxy ethylphenyl]imidazol-2-ylide), having halogenide (Cl^−^ or I^−^) or aminoacyl (Gly=N-(*tert*-Butoxycarbonyl)glycinate or Phe=(S)-N-(*tert*-Butoxycarbonyl)phenylalaninate) counterion. For AgL20, AuL20, AgM1 and AuM1, the Half-Maximal Inhibitory Concentration (IC_50_) values were measured, and all complexes seemed to reduce cell viability more effectively than Cisplatin, selected as control. The complex named AuM1 was the most active just after 8 h of treatment at 5 μM, identified as effective growth inhibition concentration. AuM1 also showed a linear dose and time-dependent effect. Moreover, AuM1 and AgM1 modified the phosphorylation levels of proteins associated with DNA lesions (H2AX) and cell cycle progression (ERK). Further screening of complex aminoacyl derivatives indicated that the most powerful were those indicated with the acronyms: GlyAg, PheAg, AgL20Gly, AgM1Gly, AuM1Gly, AgL20Phe, AgM1Phe, AuM1Phe. Indeed, the presence of Boc-Glycine (Gly) and Boc-L-Phenylalanine (Phe) showed an improved efficacy of Ag main complexes, as well as that of AuM1 derivatives. Selectivity was further checked on a non-cancerous cell line, a spontaneously transformed aneuploid immortal keratinocyte from adult human skin (HaCaT). In such a case, AuM1 and PheAg complexes resulted as the most selective allowing HaCaT viability at 70 and 40%, respectively, after 48 h of treatment at 5 μM. The same complexes tested on 3D MeWo static culture induced partial spheroid disaggregation after 24 h of culture, with almost half of the cells dead.

## 1. Introduction

Melanoma is one of the leading causes of death from skin cancer, and its incidence is increasing globally [1]. Due to the aggressiveness of melanoma, patients with this disorder are often diagnosed at advanced stages with local or even remote metastasis, when it is incurable by surgery. Indeed, patients with metastatic melanoma have a median survival time of less than 1 year [2], while the long-term survival rate is only 5% [3]. Since 2011, treatment of melanoma has been revolutionized as a result of better understanding of its biology and tumor immunology [4]. However, the therapies against malignant melanoma still lack efficacy. This is mainly because of the heterogeneity of this cancer [5], as well as metastatic dissemination [6].

Immunotherapy has been a revolutionary treatment for melanoma. Monoclonal antibodies against CTLA-4 (ipilimumab) and PD-1 (pembrolizumab, nivolumab), blocking the tumor-induced immune checkpoints, have successfully prolonged the overall survival of patients with advanced melanoma [7,8,9]. At the same time, it should be noted that checkpoint blockade can sometimes lead to serious adverse events related to autoimmune toxicities, due to non-specific immunologic activation [10]. Furthermore, melanoma is one of the most drug-resistant human cancers [11]. As half of the advanced melanoma patients bear mutations at the residue Val 600 in the kinase BRAF that lead to constitutive activation of the MAPK pathway [12], targeted therapies including BRAF inhibitors (vemurafenib, dabrafenib) and MEK inhibitors (trametinib, cobimetinib) have been developed [13,14,15,16,17], improving the overall response rates and survival compared to chemotherapy [13]. However, BRAF inhibitors have a short-term effect in most patients due to the development of resistance at a median of 5–7 months [4]. Although these new inhibitors seem to be promising therapies in the treatment of melanoma, to date, cisplatin is still used as a chemoterapic drug. Cisplatin has cytotoxic effects caused by its interactions with DNA adducts, which result in the termination of the DNA damage-mediated apoptotic program and of DNA damage repair [18,19]. However, a common characteristic of melanoma is resistance to cisplatin [20], due to its increased detoxification in cytoplasm and minimization of its accumulation in tumor tissues [19,21]. Due to the heterogeneity of melanoma tumors and the frequent development of drug resistance, the design of novel effective chemotherapies with high selectivity and lower toxicity is important. Thus, much research activity is focused on the discovery of new organometallic complexes that are less toxic than platinum compounds and have less cross-resistance. Silver and gold complexes are among those that have recently been arousing considerable interest because of these characteristics [22,23,24,25].

**Figure 1:**
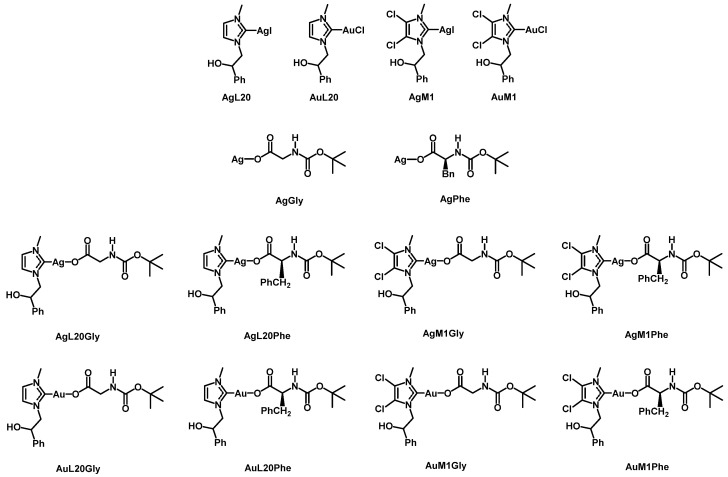
Chemical structure of silver(I) and gold(I) complexes tested in this work.

Recently, Longo and co-workers synthesized several silver and gold complexes stabilized by N-heterocyclic carbene ligands (i.e.,: [N-methyl, N′-(2-hydroxy-2-phenyl-ethyl) imidazole-2-ylidine]) and [4,5-dichloro-(N-methyl, N′-(2-hydroxy-2-phenyl-ethyl) imidazole-2-ylidine] [26,27,28]. These complexes have shown good antiproliferative activity against some carcinogenic cell lines, such as MCF-7 and MDA-MB-231 [28,29], and have been successfully included in biocompatible nanofibrous membranes for potential topical applications [30]. In this study, new derivatives of these complexes with different counterions, such as glycinates and phenylalaninates, both *tert*-Butoxycarbonyl (Boc) protected have been synthesized and named with the acronyms: AgL20Gly, AgL20Phe, AgM1Gly, AgM1Phe, AuL20Gly, Aul20Phe, AuM1Gly and AuM1Phe, to have molecules possibly more able to cross the cell membrane (see also Figure 1). Therefore, the aim of this study was to test the anti-proliferative effects of these new metallic complexes also in view of their potential use in pharmaceutical topical applications against melanoma. For this reason, we chose MeWo cells that are human malignant and chemoresistant melanoma cells from a lymph node metastatic site.

Growth of malignant tumors occurs in three-dimensional space and depends on the presence of a stromal component which performs critical functions of tumor cell protection and growth support. Therefore, development and analysis of tumor models, such as melanoma, in 3D cell cultures in vitro presents a significant interest [31]. Overall, the development of stroma-rich spheroids enlarges the arsenal of in vitro pre-clinical models for high-throughput screening of anticancer drugs [32].

In this study, MeWo cells were also used in 2D (monolayer) and 3D cultures (spheroids) to test the efficacy and selectivity of AgL20, AuL20, AgM1, AuM1 complexes and their aminoacyl derivatives at different concentrations (1 μM, 5 μM, 10 μM, 20 μM) for 24 h and 48 h, to evaluate the Minimum Inhibition Concentration (MIC) and the Half-Maximal Inhibitory Concentration (IC_50_). Time-lapse imaging was used to understand the time course of complex-induced death at the highest concentration tested, whereas Flow Cytometry and Western blot analysis were performed to study apoptosis, DNA damage and cell cycle alterations. The phosphorylation of the histone H2AX and of the kinase ERK, related to DNA Damage Response (DRR) and cell proliferation regulation, respectively, was investigated. Moreover, a spontaneously transformed aneuploid immortal keratinocyte cell line from adult human skin (HaCaT) was also selected to check the safety of all complexes and derivatives on a non-cancerous cell line, evaluating their selectivity. MeWo 3D cultures were also investigated to monitor complexes diffusion into cell aggregates.

## 2. Results and Discussion

### 2.1. Complexes Description, Synthesis and Chemical Characterizations

The description of complexes and their innovative design is illustrated in Figure 1. N-methyl, N′-[(2-hydroxy-2-phenyl)-ethyl]-imidazolium iodide and 4,5-dichloro-(N-methyl-N′-[(2-hydroxy-2-phenyl)ethyl])-imidazolium iodide, were prepared by reaction of imidazole or 4,5-dichloroimidazole with 1,2-epoxyethylbenzene, to obtain by opening of epoxy-ring the monoalkylated product. The second nitrogen atom is methylated by reaction with iodomethane. The products are a mixture of the racemic iodide salts.

**Figure 2:**
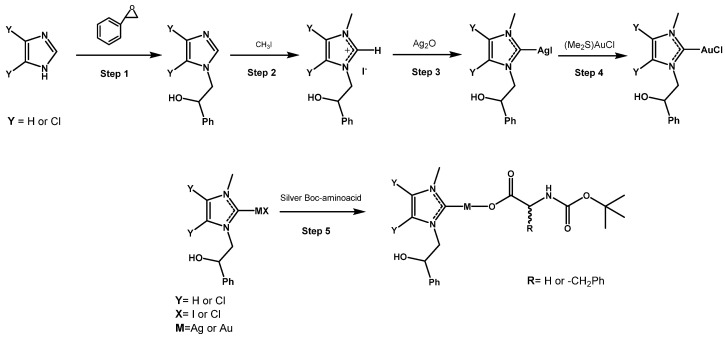
Description of the chemical synthesis of silver(I) and gold(I) complexes with amino acid anionic ligands.

In Figure 2, the reaction scheme (step 1 and 2) is reported. This synthetic strategy was previously reported by Arnold et al. [33] and was slightly modified by some of us [26,27,28]. The imidazolium salts were reacted with silver oxide (Ag_2_O), which by deprotonation of the carbon 2 gives the corresponding carbene ligands able to coordinate in situ the silver-producing [NHC-Ag]I complexes (see, Figure 2, step 3). NHC-gold(I) chloride complexes were synthesized, following the method suggested by Baker et al. [34], by transmetallation in dichloromethane (CH_2_Cl_2_) of the suitable [NHC-Ag]I complex and the gold(I)-chloro-(dimethylsulfide) [(Me_2_S)AuCl]; see Figure 2, step 4. The silver- and gold-Boc-glycinate and Boc-phenylalaninate complexes were obtained by reaction of the suitably substituted NHC-metal-halogenide with 1.2 equivalents of silver-Boc-glycinate or silver-Boc-phenylalaninate, following the slightly modified procedure reported by Hackenberg et al. [27], excluding light, at room temperature for 3 h in dry CH_2_Cl_2_. The NHC-metal-Boc-glycinates and the NHC- metal- Boc-phenylalanynates (see Figure 2, step 5) were obtained after filtration on celite of silver halogenide by evaporation of solvent in vacuo. Yields of silver amino acid complexes are around 70%, whereas those of gold complexes are about 50%.

All the synthesized products were analyzed by means of ^1^H and ^13^C NMR spectroscopy, elemental and mass analysis. NMR spectra of the metal complexes were recorded in DMSO-d_6_ at room temperature (see also Supplementary Information for the detailed data analyses). The ^1^H and ^13^C NMR spectra show the predictable signals. For all complexes, the exchange of the counterion was confirmed by the presence of the protons attributable to the *tert*-butyl group in ^1^H NMR and by the new carbon peaks in the ^13^C NMR spectra. The resonance of protons of three methyl of *tert*-butyl group was at around 1.3–1.4 ppm, whereas the carbons chemical shifts of methyls were observed at around 27–28 ppm and that of saturated quaternary carbon at about 77 ppm. As further confirmation of the exchange of the halogenides with Boc-amino acid counterions, for all the complexes a shift of the carbene carbon was detected (see also Table 1) [35]. The resonances of the carbene carbons of the amino acid derivatives are always at a higher field than those of halogenide complexes.

This is in agreement with what was assumed by Herrmann et al. [36] which suggested a shift in this direction of the resonance of carbene carbon in the ^13^C NMR spectra, due to the decrease in Lewis acidity of the metal center when the halogenide is substituted by a carboxylate. Of course, other factors determine the Lewis acidity of the metal, i.e., the properties of the NCH ligand and the oxidation state of the metal.

The results of MALDI-MS and elemental analysis are given in the experimental part. In the supporting info are shown the MALDI spectra. MALDI-MS data discloses a structure type [(NHC)_2_M]^+^, while elemental analyses of the complexes show a 1:l:1 ligand–metal–counterion relationship, so it is reasonable to assume for all complexes a structure of the type [(NHC)_2_M]^+^[MX_2_]^−^. According to these results, it is possible to hypothesize that in the solution there is an equilibrium between the ionic compound [M(NHC)_2_]^+^[MX_2_]^−^ and the neutral species M(NHC)X. The FT-IR spectra of the most active complexes are showed in the supporting information section, where also the characterizing wavenumbers of the most important functional groups are reported. No X-ray structures were performed because despite many attempts it was not possible to obtain crystals suitable for analysis X-ray.

The complexes showed also an excellent hydrolytic stability; in fact, in a 90/10 solution of DMSO-d_6_/D_2_O no degradation is observed even after 72 h. Furthermore, as shown for the complex AgM1Gly, they are thermally stable up to temperatures of around 150 °C and decompose before melting (see Figures S1–S6 in Supplementary Materials).

### 2.2. Activity on MeWo Cell Line: Viability, IC50, Apoptosis Study and DNA Lesion Impact

**Figure 3:**
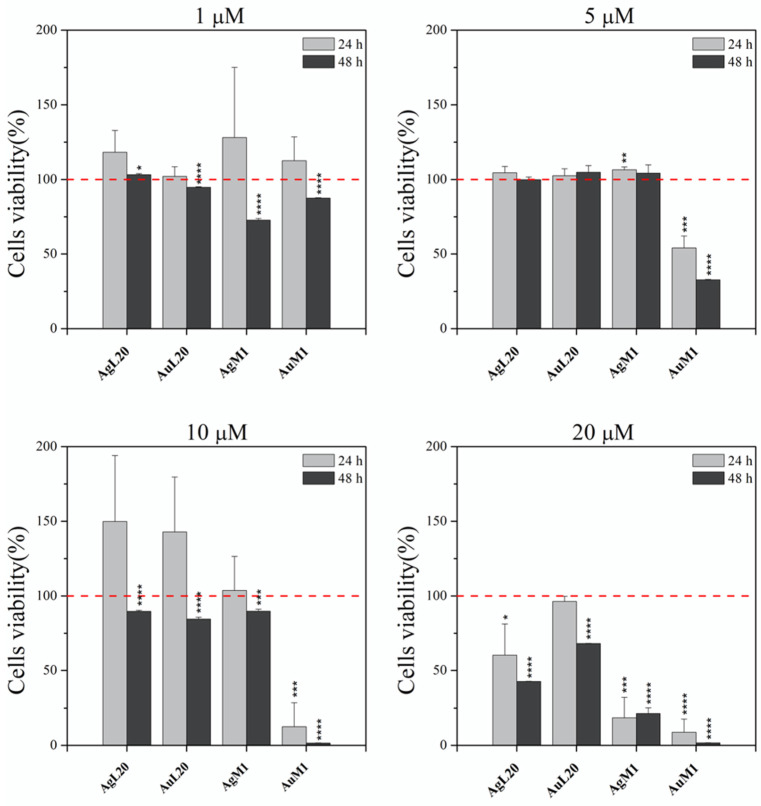
Study of the activity of different concentrations of the four main complexes on 2D MeWo culture. Cell viability was evaluated by MTT assay for the AgL20, AuL20, AgM1 and AuM1 identified as the main complexes used at different concentrations of 1 μM, 5 μM, 10 μM, 20 μM for 24 and 48 h. Statistically significant MIC of 1 μM was identified for AgM1 after 48 h. AuM1 showed a linear dose and time-dependent effect, always more evident after 48 h of treatment. Data are shown as the mean ± SD of three independent experiments. * *p* ≤ 0.05, ** *p* < 0.01, *** *p* < 0.001 and **** *p* ≤ 0.0001 vs. control = 100 (red dashed line).

To date, many metal-based anticancer drugs have been developed; however, they are mainly platinum and copper derivatives exhibiting high side effects associated with their anti-proliferative and anti-cancer effects [37]. The compounds proposed in this work are based on different metal compounds such as Ag and Au. The main four complexes (AgL20, AuL20, AgM1, and AuM1) seemed to induce an effective cell apoptosis just after few hours over treatment; therefore, to compare the results of all derivatives, we have chosen in the study a time course of 24 and 48 h. However, no different results were obtained at 72 h of treatment. In more detail, AgL20, AuL20, AgM1, and AuM1, at concentration of 20 μM, were able to inhibit MeWo viability just after 24 h. The inhibition trend in cell viability was similar also at 48 h of treatment, when AgL20, AgM1 and AuM1 showed a significant decrease in cell viability (more than 50%), while AuL20 exhibited a lower (25%) but significant reduction (Figure 3). At concentrations of 10 μM, AgL20, AuL20 and AgM1 slightly but significantly reduced cell viability of about 10% after 48 h of treatment, whereas AgL20 and AuL20 seemed to have an apparent increase in cell viability at 24 h.

AuM1 seemed the most effective complex with a reduction of 80% after 24 h and 95% after 48 h. Reducing the concentration to 5 µM, only the AuM1 complex showed an excellent behavior with a MeWo viability reduction of 70% after 48 h. The concentration of 1 µM was identified as the Minimum Inhibitory Concentration (MIC) for AgM1, that showed a more consistent reduction in cell viability (30%) at 48 h, compared to all the other complexes. AuM1 had also a clear linear dose and time-dependent effect, more evident at 48 h of treatment, suggesting it could be considered the most promising anti-proliferative compound (Figure 3). IC_50_ values were also calculated for the four main complexes at 20 µM after 24 h and 48 h of treatment and reported in Table 2. The complex that showed the lowest IC_50_ was AuM1 (see also Figure S4 in Supplementary Materials).

**Figure 4:**
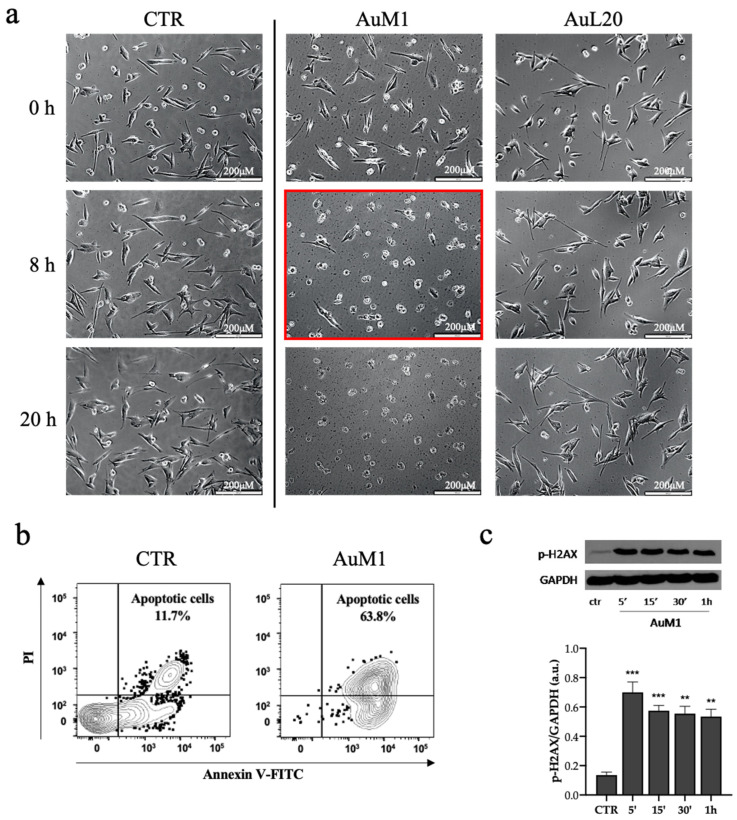
(**a**–**c**) Two-dimensional MeWo culture treated with AuM1 and AuL20. Two-dimensional MeWo culture treated with 20 μM of AuM1 and AuL20 up to 20 h and monitored by Time-Lapse Live-Cell Imaging System. In red is highlighted cell death, clearly observed after 8 h of culture with complex AuM1. Scale bar: 200 μM (**a**). Apoptosis measured by Annexin V and propidium iodide (PI) double-staining through flow cytometry in the MeWo cell line after 8 h of treatment with AuM1 (20 μM). Contour plots confirmed the time lapse data (**b**). Phosphorylation of histone H2AX measured by Western blot in the MeWo cells line after 5′-10′30′-1 h of treatment with AuM1 (20 μM) (**c**). Data are shown as the mean ± SD of three independent experiments. ** *p* < 0.01 and *** *p* < 0.001 vs. control.

Time-Lapse Live-Cell Imaging System was also adopted to monitor in detail the time necessary for the induction of MeWo cell death, when treated with 20 μM of the AuM1 compound (Figure 4a). Results confirmed that AuM1 complex was the fastest and the most active in inhibiting cell survival. Just after 8 h, MeWo underwent strong detaching, as clearly observed in the micro-cinematography video showing cells collapsing with a rapid transformation of their shape into a spherical one (see the frame highlighted in red in Figure 4a). AuL20 (at 20 μm) was less effective; indeed, living cells were found after 40 h (Figure 4a).

Based on the observed live-imaging data, to examine the apoptotic effect of AuM1, cells were treated with AuM1 for 8 h and then stained with Annexin V and propidium iodide (PI) [38]. As shown in Figure 4b, after 8 h of exposure to 20 μM of AuM1, a significant increase in the apoptosis rate of MeWo cells (63.8%) was observed compared to control (11.7%), suggesting that AuM1 possesses the ability to provoke apoptosis in human malignant and chemoresistant melanoma cells.

To provide insights into the potential mechanism of action of the Au-complex, we further investigated, by Western blot, the phosphorylation of the histone H2AX (which is known to play a key role in DNA Damage Response [39]) in cell populations treated with AuM1 at the concentration of 20 μM for 5′-15′-30′-1 h. Interestingly, we found a significant and marked increase of the histone H2AX phosphorylation already after 5 min of treatment (Figure 4c), suggesting that the apoptotic activity of AuM1 is mediated by the induction of DNA lesions-related programs and indicating its promising role as a metal-based anticancer drug.

**Figure 5:**
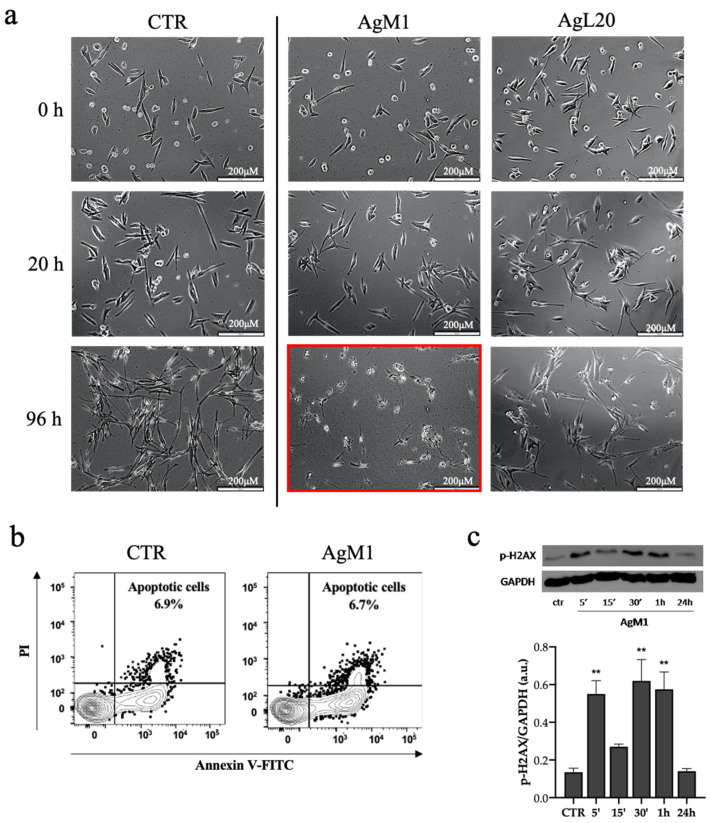
(**a**–**c**) Representative time-lapse images of 2D MeWo culture treated with AgM1 and AgL20. 2D MeWo culture treated with 20 μM of AgM1 and AgL20 up to 96 h and monitored by Time-Lapse Live-Cell Imaging System. In red is highlighted cell death, clearly observed after 96 h of culture with complex AgM1. Scale bar: 200 μM (**a**). Apoptosis measured by Annexin V and propidium iodide (PI) double-staining through flow cytometry in the MeWo cell line after 96 h of treatment with AgM1 (20 μM). Contour plots indicated a small percentage of apoptotic cells (**b**). Phosphorylation of histone H2AX measured by Western blot in the MeWo cells line after 5′-10′30′-1 h-24 h of treatment with AgM1 (20 μM) (**c**). Data are shown as the mean ± SD of three independent experiments. ** *p* < 0.01 vs. control.

For cell populations treated with 20 μm of AgM1 and AgL20, time-lapse images indicated that longer times were required to observe a change of cell morphology, which was monitored up to 96 h (see the frame highlighted in red in Figure 5a). MeWo exposed to AgM1 complex behaved differently: from the frames acquired, it was clear that the complex promoted cell death only after 96 h of treatment. AgL20, instead, seemed to not induce evident cell death even after 96 h (Figure 5a). Also in this case, apoptosis was monitored by Flow Cytometry using Annexin V/PI staining in cells exposed to AgM1 for 96 h, but data showed the presence of a small number of apoptotic cells (almost only 6.7%) after 96 h of treatment with AgM1 at 20 μM (see Figure 5b). Furthermore, the investigation of the phosphorylation of H2AX, by Western blot, at the same concentration of 20 μM, revealed that AgM1 was able to significantly increase the phosphorylation of H2AX at 5′-30′-1 h, suggesting an induction of DNA damage that cells tried to counteract by activating the H2AX enzyme (see Figure 5c).

**Figure 6:**
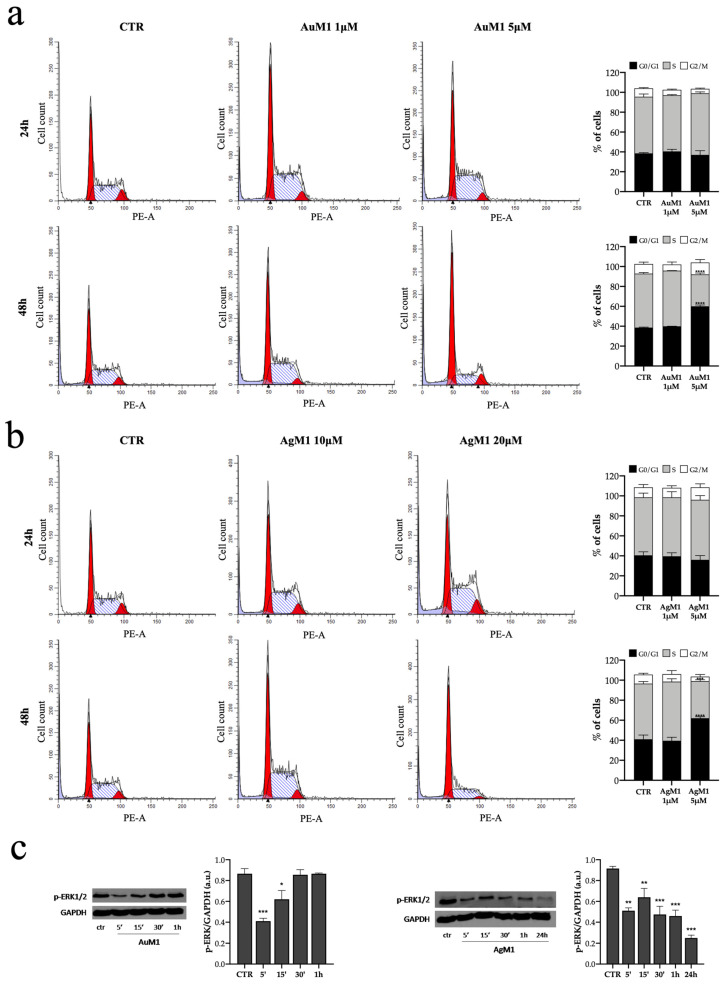
(**a**–**c**) Cell cycle progression of MeWo cells treated with 1 μΜ and 5 μΜ of AuM1 for 24 h and 48 h (**a**). Cell cycle analysis of MeWo cells treated with 10 μΜ and 20 μΜ of AgM1 for 24 h and 48 h Representative histograms of control and AuM1-treated MeWo cells are on the right (**b**). Western blot analysis of ERK phosphorylation in the MeWo cells line after treatment with 20 μM of AuM1 (5′-15′30′-1 h) and AgM1 (5′-15′30′-1 h-24 h) (**c**). Data are shown as the mean ± SD of three independent experiments. * *p* ≤ 0.05, ** *p* < 0.01, *** *p* < 0.001 and **** *p* ≤ 0.0001 vs. control.

It is well known that tumor cells originated from normal cells gain a sequence of special features, such as sustained proliferative signaling, inefficient growth suppression and replicative immortality [40], and that cell cycle regulation plays a key role in tumorigenesis as well as cancer treatment [41]. Therefore, to study cell cycle progression in cell populations exposed to AuM1 (1 μM and 5 μM) and AgM1 (10 μM and 20 μM), we performed a Flow Cytometry analysis using propidium iodide as DNA intercalative molecule [42]. In the plots reported in Figure 6a,b cell cycle progression is monitored at 24 and 48 h of treatment with the complexes. 

Interestingly, we observed that AuM1 at 5 μM and AgM1 at 20 μM induced a G0/G1 phase cell cycle arrest after 48 h of treatment, suggesting that the previously shown apoptosis (see Figure 5) occurs as a result of G0/G1 phase arrest. The molecular mechanism underlying the inhibition of cell proliferation was studied also by analyzing the phosphorylation of the extracellular-signal regulated kinase (p-ERK 1/2) [43]. According to other analyses, we found that both AuM1 and AgM1 (at the concentration of 20 μΜ) caused a pronounced and significant decrease of p-ERK (Figure 6c), suggesting that the G0/G1 phase arrest is mediated by ERK phosphorylation.

### 2.3. Activity of the Complex’s Aminoacyl Derivatives by MTT Assay

**Figure 7:**
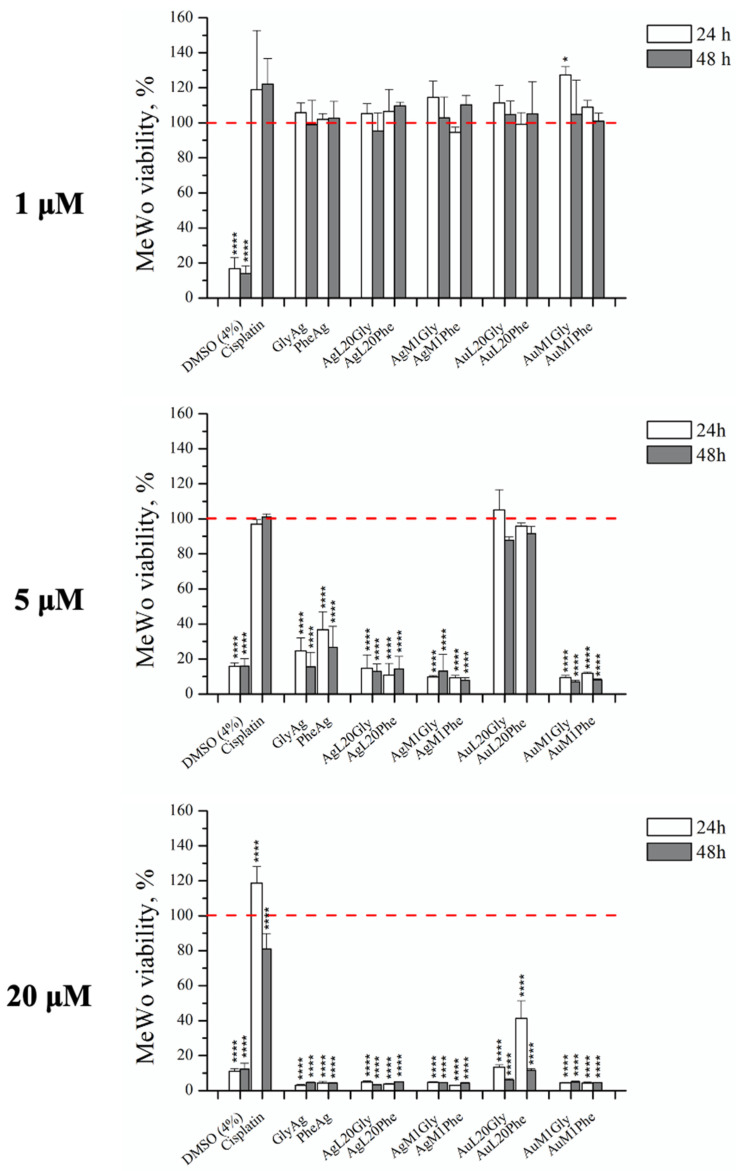
Study of the activity of different concentrations of all complex derivatives on 2D MeWo culture. Concentrations of 20 μM, 5 μM and 1 μM were screened for all the complexes. The concentration of 5 μM was able to reduce MeWo viability up to 80% for complexes derivatives such as GlyAg and PheAg, AgL20Gly, AgM1Gly, AuM1Gly, AgL20Phe, AgM1Phe and AuM1Phe. Data are shown as the mean ± SD of three independent experiments. * *p* ≤ 0.05 and **** *p* ≤ 0.0001 vs. control = 100 (red dashed line).

Following the data acquired for the four main complexes, a screening of their derivatives was performed adopting concentrations of 1, 5 and 20 μM and by using MTT assay, in order to compare their behavior with the main complexes [44,45,46]. All data are summarized in Figure 7.

In the best cases, 5 μM was able to ensure a MeWo viability reduction up to 80%. It has also to be underlined that MeWo cultures were not inhibited by cisplatin, used as negative control, even at concentration of 20 μM, whereas their growth was prevented by DMSO at 4%, selected as positive control (Figure 7). At 5 μM, the most powerful complexes seemed to be: GlyAg; AgL20Gly; AgM1Gly; AuM1Gly; PheAg; AgL20Phe; AgM1Phe; AuM1Phe (see Figure 7).

**Figure 8:**
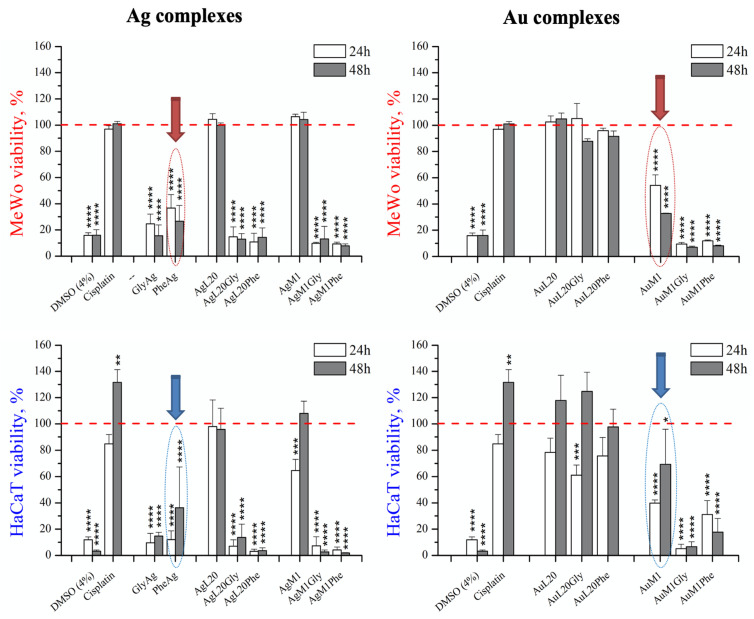
Complex selectivity study on Dermal Melanocyte (HaCaT) at concentration of 5 μM. The AuM1 and PheAg complex resulted as the most selective; indeed, 5 μM of AuM1 and PheAg at 48 h of culture reduced MeWo viability down to 30% (red arrows) but allowed HaCaT viability at 70 and 40%, respectively (blue arrows). AuM1Phe also showed slight selectivity at 48 h of culture. Data are shown as the mean ± SD of three independent experiments. * *p* ≤ 0.05, ** *p* < 0.01, *** *p* < 0.001 and **** *p* ≤ 0.0001 vs. control = 100 (red dashed line).

The same complexes were also tested for a selectivity study on primary Dermal Melanocyte (HaCaT) at a concentration of 5 μM and, in this case, PheAg and AuM1 complexes performed as the most selective, as indicated by HaCaT viability of 40 and 70%, respectively. All complexes showed a greater effect than cisplatin (Figure 8).

The complexes with amino acids Gly and Phe were found to inhibit also HaCaT viability. On the contrary, PheAg and AuM1 complexes at 48 h seemed more effective on MeWo than on HaCaT, suggesting that they act on specific targets of cancer cells. However, further studies are necessary to proper investigate specific complex uptakes and their proper mechanism of actions.

### 2.4. Activity of AuM1, PheAg and AuM1Phe on 3D MeWo Culture

AuM1, PheAg and AuM1Phe were identified as complexes with a good performance and selectivity against MeWo, inhibiting HaCaT cell survival to a marked lesser extent. Therefore, these complexes were also selected to be investigated on MeWo 3D culture, to monitor spheroid viability. Based on the previously described results, the concentration of 5 μM was adopted for all complexes studied. Indeed, efficacy of these complexes on MeWO 3D culture may provide an indirect data on complex diffusion through cellular aggregates. Indeed, despite complexes high water solubility and stability (at effective concentration values) may suggest a high bioavailability; often, the diffusion into cellular aggregates (i.e., tumor 3D structure) is extremely complex [47]. One of the first challenges is to cross the tumor interstitium or extra-cellular matrix (ECM), consisting of cross-linked network of collagen and elastin fibers, proteoglycans and hyaluronic acid. This structure not only provides structural integrity, but also helps to transport important nutrients as well as oxygen to support cell growth. A highly developed matrix may result in significant resistance to the diffusion of therapeutic particles through the interstitium causing the drug-cargo to be released too far from the tumor space to have its intended effect [47].

**Figure 9:**
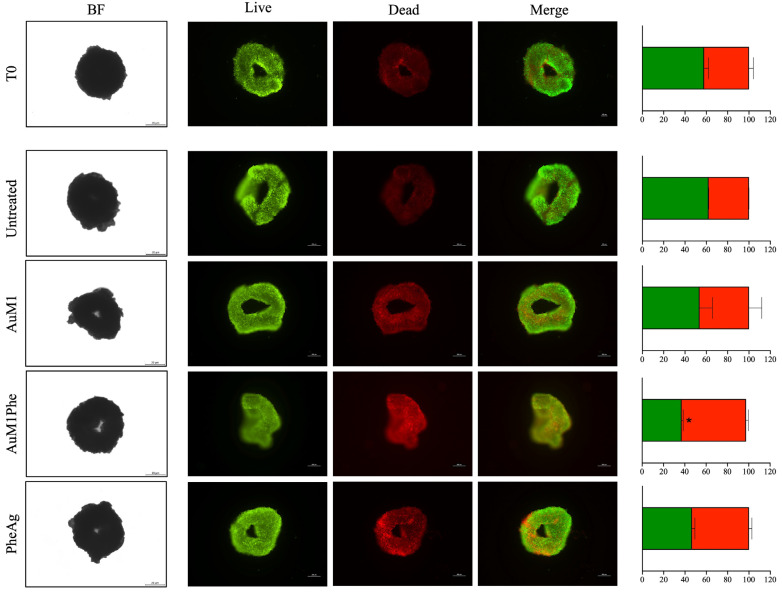
Live/Dead assay on 3D MeWo cultures treated with AuM1, AuM1Phe and PheAg for 24 h. Activity of AuM1, AuM1Phe and PheAg was investigated on 3D MeWo cultures adopting the concentration of 5 μM. Brightfield images of spheroids (Scale bar: 20 μm) and Live/Dead images (viable cells appear green, non-viable cells in red; scale bar: 200 μm) were obtained along the culture time. Data are shown as the mean ± SD of three independent experiments. * *p* ≤ 0.05.

Representative brightfield images of MeWo 3D culture assembled are illustrated in Figure 9 (left side, T0). After 24 h of treatment, 3D cultures underwent Live and Dead assay, highlighting live cells in green and dead ones in red. From the images, it is clear that all complexes partially induced 3D culture death after 24 h. Histograms, indicating a semi-quantitative analysis of live and dead cells, are also illustrated in Figure 9 (right side) for all tested complexes in 3D culture. In addition, 45%, 60% and 50% of red signal was detected for AuM1, AuM1Phe and PheAg, respectively, after 24 h of treatment. However, only the variations in cell death observed for AuM1Phe seemed to be statistically significant.

**Figure 10:**
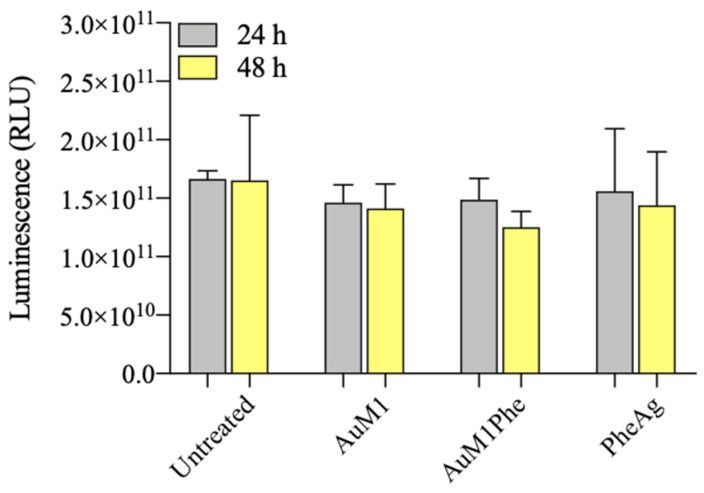
ATP assay on 3D MeWo cultures treated with AuM1, AuM1Phe and PheAg for 24 h and 48 h. Three-dimensional MeWo cultures were treated with a medium supplemented with AuM1, AuM1Phe and PheAg at 5 μM and their ATP content was measured after treatment. The histogram reports the mean of relative luminescence units compared to control (untreated cells). Data are shown as the mean ± SD of three independent experiments.

Three-dimensional culture viability was also determined by an adenosine-5′-triphosphate (ATP) quantitative assay and illustrated in Figure 10. In this case, ATP is quantified by measuring the light produced through its reaction with the naturally occurring firefly enzyme luciferase using a luminometer. The amount of light produced is directly proportional to the amount of biological energy present in the sample. Compared to the quantitative analysis of Live and Dead assay, the recorded values of Relative Luminescence Units (RLU) seemed to follow a similar trend for the three complexes tested, more evident at 48 h. However, the data obtained lack of statistical significance.

The proposed data suggested that: despite the fact that complexes were soluble in water and, therefore, an enhanced diffusion in biologicals fluids should be expected, often biological barriers occur and a reduced mass transfer into 3D cells aggregates may happen [48]. This fact may prevent proper complex diffusion into 3D culture, reducing their effectiveness and activity. This event opens future perspectives for the requirement of a proper formulation with specific lipid carriers to improve complexes targeting into a 3D environment. In this sense, enhanced and new metallic complexes coupled with lipid-based or polymeric nanoparticles can help a more apt delivery, entering the era of precision medicine [49].

## 3. Materials and Methods

### 3.1. Chemical Synthesis Protocols

All reactions were performed under nitrogen atmosphere using standard Schlenk technique. The synthesis involving silver salts were carried out with exclusion of light. All chemicals were purchased from Sigma-Aldrich Darmstadt, Germany and TCI Europe Zwijndrecht Belgium and used without further purifications. Solvents were dried and distilled before use. Deuterated solvents were degassed under a N_2_ flow and stored over activated 4 Å molecular sieves. NMR spectra were recorded on a Bruker (Milano, Italy) AM 300 spectrometer (300 MHz for ^1^H; 75 MHz for ^13^C) and Bruker (Milano, Italy) AVANCE 400 spectrometer (400 MHz for ^1^H; 100 MHz for ^13^C). NMR samples were prepared by dissolving about 15 mg of compounds in 0.6 mL of deuterated solvent. In addition, 1H and 13C chemical shifts are listed in parts per million (ppm) using the residual protio impurities of the deuterated solvents as internal standard (DMSO-d_6_ ^1^H *δ*_H_ = 2.50, ^13^C *δ*_C_ = 39.52). Multiplicities are abbreviated as follows: singlet (s), doublet (d), triplet (t), multiplet (m), broad (br). All reactions were performed under nitrogen using standard Schlenk. The synthesis involving silver salts were carried out with exclusion of light. All chemicals were purchased from Sigma-Aldrich and TCI and used without further purifications. Solvents were dried and distilled before use. Deuterated solvents were degassed under a N_2_ flow and stored over activated 4 Å molecular sieves. NMR spectra were recorded on a Bruker AM 300 spectrometer (300 MHz for ^1^H; 75 MHz for ^13^C) and Bruker AVANCE 400 spectrometer (400 MHz for ^1^H; 100 MHz for ^13^C). NMR samples were prepared by dissolving about 15 mg of compounds in 0.6 mL of deuterated solvent. ^1^H and ^13^C chemical shifts are listed in parts per million (ppm) using the residual protio impurities of the deuterated solvents as internal standard (DMSO-d_6_ ^1^H *δ*_H_ = 2.50, ^13^C *δ*_C_ = 39.52). Multiplicities are abbreviated as follows: singlet (s), doublet (d), triplet (t), multiplet (m), broad (br).

The elemental analysis for C, H, N was conducted with a Thermo-Finnigan Flash EA 112 in according to analytic procedures. Chloride was determined by the precipitation reaction with AgNO_3_, which was dissolved in Na_2_S_2_O_3_. The amount of silver was determined by flame atomic absorption spectroscopy (FAAS), while the quantity of halogen was calculated using the content of silver.

MALDI-MS were recorded using Brucker (Milano, Italy) Solarix XR Fourier transformation ion cyclotron resonance mass spectrometer (Brucker Daltonic GmbH, Bremen, Germany) fit out with a 7T cooled actively shielded superconducting magnet (Bruker Biospin, Wissembourg, France). The ionization of the samples was performed in positive ion mode using appropriate MALDI ion source (Brucker Daltonic GmbH, Bremen, Germany). The range of the spectra was set to *m/z* 200–3000. The laser power was 28% and 20 lased shots were used for each sample. The spectra were calibrated using linear calibration using a mix a peptide cluster in MALDI positive ionization mode. The accuracy of the measurements was improved by internal recalibration through matrix ionization (2,5-dihydrobenzoic acid).

### 3.2. General Procedure of Synthesis AgNHC with Boc-Protected Amino Acids

Boc-protected amino silver salts (Boc-Glycine silver salt and Boc-L-Phenylalanine silver salt) were synthesized in according to the literature procedure [50]. The formation of the product was verified by NMR analysis (see Supplementary Materials).

The synthesis of silver NHC complexes (AgL20 and AgM1) and gold NHC complexes (AuL20 and AuM1) were performed by following the literature procedure [26,27,28].

The Boc-amino acid silver(I) NHC complexes (AgL20Gly, AgL20Phe, AgM1Gly and AgM1Phe) were obtained by counterion exchange by silver NHC complexes and Boc-amino acid silver salt, following a slightly modified synthetic strategy present in literature [51,52]. NHC silver(I) iodide complex (AgL20 or AgM1) (0.20 mmol) and Boc-Glycine or Boc-L-Phenylalanine silver salt (0.22 mmol) were dissolved in 30 mL of CH_2_Cl_2_ and stirred for 3 h, with exclusion of light, at room temperature. The mixture was filtered to remove AgI byproduct. The silver NHC Boc-glycinate or Boc-phenylalaninate complex was obtained by removing of solvent at reduced pressure.

[N-methyl, N′-(2-hydroxy-2-phenyl) ethyl)-imidazole-2-ylidine silver(I)] Boc-glycinate (AgL20Gly)

Yield: 70%. ^1^H-NMR (400 MHz, DMSO-d_6_, ppm): δ 7.35–7.33 (m, 7H, C_6_**H_5_**CH(OH)CH_2_NC**H**C**H**N); 6.37 (t, J*_vic_* = 5.0 Hz, 1H, Gly: N**H**); 5.85 (d, 1H, C_6_H_5_CH(O**H**)CH_2_N), 5.01 (m, J*_vic_* = 10.3, 8.4 Hz, 1H, C_6_H_5_C**H**(OH)CH_2_N); 4.41 (m, J*_gem_* = 15.0 Hz, J*_vic_* = 10.3, 8.4 Hz, 2H, C_6_H_5_CH(OH)C**H_2_**N); 3.90 (s, 3H, NC**H_3_**); 3.51 (d, J*_gem_* = 11.0 Hz, J*_vic_* = 5.0 Hz, 2H, Gly: NHC**H_2_**); 1.29 (s, 9H, Gly: C(C**H_3_**)_3_).

^13^C-NMR (100 MHz, DMSO-d_6_, ppm): δ 178.4 (N**C**N); 175.4 (**C**OOAg); 154.9 (NH**C**O); 142.2, 139.2, 128.2, 127.5, 125.8 (**Ph ring**); 122.9, 122.2 (N**C**H**C**HN); 77.4 (**C**(CH_3_)_3_); 72.6 (Ph**C**HOH); 58.2 (N**C**H_2_); 45.2 (NH**C**H_2_); 38.1 (N**C**H_3_); 28.2 (C(**C**H_3_)_3_).

MALDI-MS (*m*/*z*): 513.12995 attributable to bis-carbene structure [C_24_H_28_AgN_4_O_2_]^+^ and 478.74477 attributable to [C_22_H_22_AgN_4_O_2_]^+^

Elemental Analysis: theorical = C: 47.12, H 5.41, Ag 22.27, N 8.68, O 16.52; experimental = C 47.08, H 5.45, Ag 22.17, N 8.78, O 16.52.

[N-methyl, N′-(2-hydroxy-2-phenyl) ethyl)-imidazole-2-ylidine silver(I)] Boc-phenylalaninate (AgL20Phe)

Yield: 67%. ^1^H-NMR (400 MHz, DMSO-d_6_, ppm): δ 7.40–7.17 (m, 12 H, C_6_**H_5_**CH(OH)CH_2_NC**H**C**H**N + Phe:C_6_**H_5_**); 6.20 (t, J*_vic_* = 8.0 Hz, 1H, Phe: N**H**); 5.77 (d, 1H, C_6_H_5_CH(O**H**)CH_2_N); 4.95 (m, J*_vic_* = 10.5, 8.7 Hz, 1H, C_6_H_5_C**H**(OH)CH_2_N); 4.26, 4.16 (dd, J*_gem_* = 15.6 Hz, J*_vic_* = 10.5, 8.7 Hz, 2H, C_6_H_5_CH(OH)C**H_2_**N); 4.01 (dd, J*_vic_* = 9.0, 8.5 Hz 1H, Phe: C**H**CH_2_Ph); 3.74 (s, 3H, NC**H_3_**); 3.09, 2.88 (dd, J*_gem_* = 14.0 Hz, J*_vic_* = 9.0, 8.5 Hz, 2H, Phe: CHC**H**_2_Ph); 1.39 (s, 9H, Phe: C(C**H_3_**)_3_).

^13^C-NMR (100 MHz, DMSO-d_6_, ppm): δ 178.4 (N**C**N); 175.4 (**C**OOAg); 154.9 (NH**C**O); 142.2; 139.2, 129.3, 128.2, 127.8, 127.5, 126.1, 125.8 (**Ph** rings); 124.2, 122.9 (N**C**H**C**HN); 77.4 (**C**(CH_3_)**_3_**); 72.5 (Ph**C**HOH); 58.2 (N**C**H_2_); 56.4 (NH**C**H); 38.1 (N**C**H_3_); 37.9 (CH**C**H_2_Ph); 28.3 (C(**C**H_3_)_3_).

MALDI-MS (*m*/*z*): 511.52195 attributable to bis-carbene structure [C_24_H_28_AgN_4_O_2_]^+^

Elemental Analysis: theorical = C: 54.36, H 5.62, Ag 18.78, N 7.32, O 13.92; experimental = C 54.30, H 5.68, Ag 18.75, N 7.35, O 13.92.

4,5-dichloro [N-methyl, N′-(2-hydroxy-2-phenyl) ethyl)-imidazole-2-ylidine silver(I)] Boc-glycinate (AgM1Gly)

Yield: 74%. ^1^H-NMR (400 MHz, DMSO-d_6_, ppm): δ 7.37–7.35 (m, 5H, C_6_**H_5_**CH(OH)CH_2_N); 6.34 (t, J*_vic_* = 5.6 Hz, 1H, Gly: N**H**); 5.97 (s, 1H, C_6_H_5_CH(O**H**)CH_2_N); 4.97 (m, J*_vic_* = 10.7, 8.6 Hz, 1H, C_6_H_5_C**H**(OH)CH_2_N); 4.25 (m, J*_gem_* = 13.8 Hz, J*_vic_* = 10.7, 8.6 Hz, 2H, C_6_H_5_CH(OH)C**H_2_**N); 3.81 (s, 3H, NC**H_3_**); 3.25 (s, 2H, Gly: NHC**H**_2_); 1.36 (s, 9H, Gly: C(C**H_3_**)_3_).

^13^C-NMR (100 MHz, DMSO-d_6_, ppm): δ: 180.7 (N**C**N); 173.7 (**C**OOAg); 155.4 (NH**C**O); 141.5, 128.8, 127.7, 126.1 (**Ph** ring); 117.4, 116.5 (N**C**Cl**C**ClN); 77.4 (**C**(CH_3_)_3_); 72.0 (C**H**OH); 56.9 (N**C**H_2_); 43.7 (NH**C**H_2_); 37.6 (N**C**H_3_); 28.2 (C(**C**H_3_)_3_).

MALDI-MS (*m*/*z*): 669.11051 attributable to bis-carbene structure [C_24_H_23_AgCl_4_N_4_NaO_2_]^+^ and 525.12643 attributable to [C_23_H_21_AgClN_4_O_2_]^+^

Elemental Analysis: theorical = C: 41.25, H 4.37, Ag 19.50, Cl 12.82, N 7.60, O 14.46; experimental = C 41.22, H 4.31, Ag 19.49, Cl 12.82, N 7.70, O 14.46.

4,5-dichloro [N-methyl, N′-(2-hydroxy-2-phenyl) ethyl)-imidazole-2-ylidine silver(I)] Boc-phenylalaninate (AgM1Phe)

Yield: 68%. ^1^H-NMR (400 MHz, DMSO-d_6_, ppm): δ 7.43–7.20 (m, 10H, C_6_**H_5_**CH(OH)CH_2_N + Phe:C_6_**H_5_**); 6.30 (m, J*_vic_* = 8.6 Hz, 1H, Phe: N**H**); 5.84 (d, 1H, C_6_H_5_CH(O**H**)CH_2_N); 4.99 (m, J*_vic_* = 10.8, 8.6 Hz, 1H, C_6_H_5_C**H**(OH)CH_2_N); 4.27 (m, 2H, C_6_H_5_CH(OH)C**H_2_**N); 4.04 (m, 1H, Phe: C**H**CH_2_Ph); 3.80 (s, 3H, NC**H_3_**); 3.11–2.87 (dd, 2H, Phe: CHC**H_2_**Ph); 1.31 (s, 9H, Phe: C(C**H**_3_)_3_).

^13^C-NMR (100 MHz, DMSO-d_6_, ppm): δ 180.7 (N**C**N); 175.3 (**C**OOAg); 155.0 (NH**C**O); 141.5, 139.0, 129.2, 128.8, 128.3, 127.9, 126.1, 125.9 (**Ph** rings); 117.4, 116.6 (N**C**Cl**C**ClN); 77.4 (**C**(CH_3_)_3_); 72.8 (**C**HOH); 57.0 (N**C**H_2_); 56.2 (**C**HNH); 37.7 (N**C**H_3_); 37.0 (**C**H_2_Ph); 28.1 (C(**C**H_3_)_3_).

MALDI-MS (*m*/*z*): 646.11051 attributable to bis-carbene structure [C_24_H_24_AgCl_4_N_4_O_2_]^+^

Elemental Analysis: theorical = C: 48.54, H 4.70, Ag 16.77, Cl 11.02, N 6.53, O 12.43; experimental = C 48.55, H 4.80, Ag 16.71, Cl 11.02, N 6.55, O 12.37.

### 3.3. General Procedure of Synthesis AuNHC with Boc-Protected Amino Acids

The gold complexes were synthesized following the procedure published in literature [52]. A solution of AuNHC complex (AuL20 or AuM1) (0.10 mmol) in CH_2_Cl_2_ (25 mL) is added at once to Boc-protected amino acid silver salt (0.11 mmol) at the dark and room temperature. After this time, the mixture was filtered to remove AgCl byproduct, and the gold complexes were obtained by removing of the solvent at reduced pressure.

Synthesis of [N-methyl, N′-(2-hydroxy-2-phenyl) ethyl)-imidazole-2-ylidine gold(I)] Boc-glycinate (AuL20Gly)

Yield: 45% ^1^H-NMR (400 MHz, DMSO-d_6_, ppm): δ 7.35–7.33, (m, 7H, C_6_**H_5_**CH(OH)CH_2_NC**H**C**H**N); 6.87 (br, 1H, Gly: N**H**); 5.85 (br, 1H, C_6_H_5_CH(O**H**)CH_2_N), 5.01 (m, 1H, C_6_H_5_C**H**(OH)CH_2_N); 4.41 (m 2H, C_6_H_5_CH(OH)C**H_2_**N); 3.90 (s, 3H, NC**H_3_**); 3.51 (d, J*_gem_* = 11.0 Hz, J*_vic_* = 5.0 Hz, 2H, Gly: NHC**H_2_**); 1.29 (s, 9H, Gly: C(C**H_3_**)_3_).

^13^C-NMR (100 MHz, DMSO-d_6_, ppm): δ 175.4 (**C**OOAu); 164.5 (N**C**N); 155.3 (NH**C**O);, 142.5; 129.9, 127.6, 126.5 (**Ph** ring); 123.1, 122.5 (N**C**H**C**HN); 77.4 (**C**(CH_3_)_3_); 72.7 (**C**HOH); 56.3 (N**C**H_2_); 43.6 (NH**C**H_2_); 37.2 (N**C**H_3_); 28.5 (C(**C**H_3_)_3_).

MALDI-MS (*m*/*z*): 601.19069 attributable to bis-carbene structure [C_24_H_28_AuN_4_O_2_]^+^

Elemental Analysis: theorical = C: 39.80, H 4.57, Au 34.35, N 7.33, O 13.95; experimental = C 39.75, H 4.82, N 7.30.

Synthesis of [N-methyl, N′-(2-hydroxy-2-phenyl) ethyl)-imidazole-2-ylidine gold(I)] Boc-phenylalaninate (AuL20Phe)

Yield: 53%. ^1^H-NMR (300 MHz, DMSO-d_6_, ppm): δ 7.40–7.17 (m, 12 H, C_6_**H_5_**CH(OH)CH_2_NC**H**C**H**N + Phe:C_6_**H_5_**); 6.20 (br, 1H, Phe: N**H**); 5.77 (d, 1H, C_6_H_5_CH(O**H**)CH_2_N); 5.10 (br, 1H, C_6_H_5_C**H**(OH)CH_2_N); 4.26–4.16 (dd, J*_gem_* = 14.6 Hz, J*_vic_* = 10.6 Hz, 8.9 Hz, 2H, C_6_H_5_CH(OH)C**H_2_**N); 4.01 (br, 1H, Phe: C**H**CH_2_Ph); 3.74 (s, 3H, NC**H_3_**); 3.09–2.88 (dd, J*_gem_* = 14.0 Hz, J*_vic_* = 9.3 Hz, 8.7 Hz, 2H, Phe: CHC**H**_2_Ph); 1.39 (s, 9H, Phe: C(C**H_3_**)_3_).

^13^C-NMR (100 MHz, DMSO-d_6_, ppm): δ 175.4 (**C**OOAu); 164.4 (N**C**N); 154.9 (NH**C**O); 142.2; 139.2, 129.3, 128.2, 127.8, 127.5, 126.1, 125.8 (**Ph** rings); 124.2, 122.9 (N**C**H**C**HN); 77.4 (**C**(CH_3_)_3_); 72.5 (**C**HOH); 58.2 (N**C**H_2_); 56.4 (NH**C**H); 38.1 (N**C**H_3_); 37.9 (Ph**C**H_2_); 28.3 (C(**C**H_3_)_3_).

MALDI-MS (*m/z*): 601.18965 attributable to bis-carbene structure [C_24_H_28_AuN_4_O_2_]^+^

Elemental Analysis: theorical = C: 47.06, H 4.86, Au 29.68, N 6.33, O 12.07; experimental = C 47.05, H 4.86, N 6.40.

Synthesis of 4,5-dichloro [N-methyl, N′-(2-hydroxy-2-phenyl) ethyl)-imidazole-2-ylidine gold(I)] Boc-glycinate (AuM1Gly)

Yield: 48%. ^1^H-NMR (300 MHz, DMSO-d_6_, ppm): δ 7.37–7.35 (m, 5H, C_6_**H_5_**CH(OH)CH_2_N); 6.84 (br, 1H, Gly: N**H**); 5.97 (br, 1H; C_6_H_5_CH(O**H**)CH_2_N); 5.20 (m, J*_vic_* = 10.7 Hz, 8.6 Hz, 1H, C_6_H_5_C**H**(OH)CH_2_N); 4.25 (m, J*_gem_* = 13.9 Hz, J*_vic_* = 10.7 Hz, 8. 6Hz, 2H, C_6_H_5_CH(OH)C**H_2_**N); 3.81 (s, 3H, NC**H_3_**); 3.25 (s, 2H, Gly: NHC**H**_2_); 1.36 (s, 9H, Gly: C(C**H_3_**)_3_).

^13^C-NMR (75 MHz, DMSO-d_6_, ppm): δ 173.7 (**C**OOAu); 163.2 (N**C**N); 155.5 (NH**C**O); 141.2, 128.8, 127.7, 125.1 (**Ph** rings); 117.4, 116.5 (N**C**Cl**C**ClN); 77.5 (**C**(CH_3_)_3_); 72.1 (**C**HOH); 56.6 (N**C**H_2_); 44.1 (NH**C**H_2_); 37.2 (N**C**H_3_); 28.3 (C(**C**H_3_)_3_).

MALDI-MS (*m*/*z*): 739.03137 attributable to bis-carbene structure [C_24_H_24_AuCl_4_N_4_O_2_]^+^

Elemental Analysis: theorical = C: 35.53, H 3.77, Au 30.67, Cl 11.04, N 6.54, O 12.45; experimental = C 35.57, H 3.80, Cl 11.10, N 6.58.

Synthesis of 4,5-dichloro [N-methyl, N′-(2-hydroxy-2-phenyl) ethyl)-imidazole-2-ylidine gold(I)] Boc-phenylalaninate (AuM1Phe)

Yield: 50%. ^1^H-NMR (400 MHz, DMSO-d_6_, ppm): δ 7.43–7.20 (m, 10H, C_6_**H_5_**CH(OH)CH_2_N + Phe:C_6_**H_5_**); 6.30 (br, 1H, Phe: N**H**); 5.84 (br, 1H, C_6_H_5_CH(O**H**)CH_2_N); 4.27 (m, 2H, C_6_H_5_CH(OH)C**H_2_**N); 4.04 (m, J*_vic_* = 8.6 Hz, 7.8 Hz, 6.8 Hz, 1H, Phe: C**H**CH_2_Ph); 3.80 (s, 3H, NC**H_3_**); 3.11–2.87 (dd, J*_gem_* = 14.8 Hz, J*_vic_* = 8.6 Hz, 7.8 Hz, 1H, Phe: C**H**CH_2_Ph); 1.31 (s, 9H, Phe: C(C**H_3_**)_3_).

^13^C-NMR (75 MHz, DMSO-d_6_, ppm): δ 175.0 (**C**OOAu); 163.3 (N**C**N); 155.1 (NH**C**O); 141.3, 138.8, 129.2, 128.2, 127.9, 125.9 (**Ph** rings); 117.5, 116.5 (N**C**Cl**C**ClN); 77.6 (**C**(CH_3_)_3_); 72.1 (**C**HOH); 56.6 (N**C**H_2_); 52.0 (**C**HNH); 37.2 (N**C**H_3_); 37.0 (**C**H_2_Ph); 28.2 (C(**C**H_3_)_3_).

MALDI-MS (*m/z*): 739.03225 attributable to bis-carbene structure [C_24_H_24_AuCl_4_N_4_O_2_]^+^

Elemental Analysis: theorical = C: 41.86, H 3.79, Au 27.46, Cl 9.88, N 5.86, O 11.15; experimental = C 41.90, H 3.80, Cl 9.87, N 5.90.

### 3.4. Complex Activity on 2D Cell Culture

Human melanoma cells (MeWo; ATCC^®^, HTB-65^TM^) and human keratinocyte cells (HaCaT; hTERT-immortalized Dermal Melanocyte ATCC^®^ CRL-4059™ Homo S., Thermo Fisher Scientific, Waltham, MA, USA), were seeded in 96-well plates at a density of 100.000 cells/mL. Cells were cultured in DMEM high glucose (Gibco^TM^, Walthan, MA, USA) supplemented with 10% Fetal Bovine Serum (Gibco^TM^, Walthan, MA, USA), 1% Penicillin/Streptomycin (Corning, Manassas, VA, USA) and 1% Glutagro^TM^ (Corning, Manassas, VA, USA) at 37 °C in a 5% CO_2_ atmosphere. Cells were left to adhere overnight and then treated with different concentrations of each metallic complex (1, 5, 10, 20 μM) for 24 h and 48 h. IC_50_ values for MeWo cells were calculated with GraphPad Prism software (6.0 for Windows, LLC, San Diego, CA, USA).

### 3.5. Cytotoxicity Assay

After the treatment, 3-(4,5-Dimethylthiazol-2-yl)-2,5-diphenyl-tetrazolium bromide (MTT) solution was added to each well at a final concentration of 0.5 mg/mL, and incubated at 37 °C for additional 4 h, protecting the plate from light. Then, the supernatant was removed and 100 μL of dimethyl sulfoxide (DMSO) was added to solubilize formazan crystals. The absorbance was measured at 490 nm using a microplate reader (Infinite F200 PRO, Tecan Group Ltd., Männedorf, Switzerland) Cell metabolic activity was calculated as percentage compared to the control group (considered as 100%), according to Equation (1):

(1) $$\%\;\mathrm{Cell}\;\mathrm{metabolic}\;\mathrm{activity}=\frac{Abs\;of\;sample-Abs\;blank}{Abs\;of\;control-Abs\;blank}\times 100$$

### 3.6. Time-Lapse Live-Cell Imaging System Assay

MeWo culture was performed using Time-Lapse Live-Cell Imaging System formed by a Bold Line Top Stage Incubator for 35 mm Petri dishes (H301-T UNIT BL; Okolab S.r.l., Pozzuoli, Italy), that allows the acquisition of the same field along the culture time in fixed culture point mapped by the fully automated stage. The incubator has independent control of gas (CO_2_/O_2_), humidity and temperature, and ensured an environment with 37 °C of temperature and 5% of CO_2_ atmosphere. The system allows acquisition in brightfield and fluorescence. All images of different fields within the culture chamber were achieved automatically, using Olympus IX83 time-lapse microscope by motorized stage and CCD monochrome camera (mod. XM10, Olympus, Segrate, Italy), and with all operations under the control of the X-Excellence advanced live-cell imaging software (version 2.0, Olympus Inc., Segrate, Italy). In our case, cell images were captured in brightfield using a 10× objective every 4 h intervals. The related videos reported in Supplementary Materials were generated with windows movie maker software (version 2.0, Microsoft) starting from the acquired frames. 

### 3.7. Apoptosis Assay

Annexin V FITC Apoptosis Detection Kit (Cat. AD10, Dojindo Laboratories, Rockville, MD, USA) was used to evaluate MeWo cell apoptosis after 24 h and 48 h of treatment with AgL20, AuL20, AgM1 and AuM1 (20 μM). Then, 5 μL of Annexin V FITC Conjugate and 5 μL of propidium Iodide PE Solution were added to 100 μL of the cell suspension (1 × 10^6^ cells/mL). After incubation for 15 min at room temperature with light protection, 400 μL of Annexin V Binding Solution was added for flow cytometry analysis.

### 3.8. Cell Cycle Analysis by DNA Content (Propidium Iodide)

Human melanoma cells (MeWo, HTB-65^TM^; ATCC^®^, Manassas, VA, USA) were grown in 24-well plates at a density of 1 × 10^4^ cells/cm^2^ and treated with AuM1 (at 1 μM and 5 μM) and AgM1 (at 10 μM and 20 μM) for 24 h and 48 h. After treatments, cells were transferred in 15 mL polypropylene V-bottomed tubes, rinsed twice in ice cold PBS, and stained in freshly prepared PBS buffer containing 1 mg/mL propidium iodide (PI) (Merck Life Science S.r.l., Milan, Italy), 110,000 U/mL RNase A (Merck) and 0.1% Triton X-100 (Merck Life Science S.r.l., Milan, Italy). Cells were incubated 30 min at 4 °C in the dark and analysed with a BD FACSVerse flow cytometer (Becton Dickinson, BD, Franklin Lakes, NJ, USA) counting 1 × 10^4^ events per sample. G1, S and G2/M cell cycle phase percentages were analysed by ModFit LT software (version 3.0). 

### 3.9. Western Blot

Human melanoma cells (MeWo; ATCC^®^, HTB-65^TM^) were grown in 100 mm Petri Dishes at a density of 1.5 × 10^4^ cells/cm^2^ and treated with 20 μM of AuM1 (5′-15′-30′-1 h) and AgM1 (5′-15′-30′-1 h-24 h). After treatments, cells were harvested and lysed in RIPA buffer (NaCl 150 mM, 1% triton X-100 pH 8.0, 0.5% sodium deoxycholate, 0.1% SDS, 50 mM Tris, pH 8.0), supplemented with protease inhibitors cocktail and phosphatase inhibitors (Merck), 1 h on ice. Lysates were then centrifuged for 30 min at 15,000× *g* and supernatant were transferred to a new 1.5 mL tube. Protein amount was determined by Bradford assay (Bio-Rad, Hercules, CA, USA) and 15 μg of total protein extracts were separated by SDS–PAGE gels and transferred onto nitrocellulose membranes. Nitrocellulose blots were blocked with 10% nonfat dry milk in TBS-T buffer (20 mM Tris-HCl, pH 7.4, 500 mM NaCl and 0.1% Tween-20), and incubated in TBS-T containing 5% nonfat dry milk overnight at 4 °C with the following primary antibodies: anti-phospho ERK42/44 (sc43765) and anti-GAPDH (sc365062), provided from Santa Cruz Biotechnology, and anti-phospho H2AX (ab26350 Abcam). Immunoreactivity was detected by sequential incubation with appropriate horseradish peroxidase-conjugated secondary antibodies (Merck) for 1 h at room temperature and Pierce ECL detection reagents (Thermo Scientific, Rockford, IL, USA) on an X-ray film. Densitometry of bands was performed with ImageJ software (NIH, Bethesda, MD, USA; version 2.0.0-rc-54/1.51 h). The area under the curves, each relative to a band, was determined and the background was subtracted from the calculated values.

### 3.10. Complexes Activity on High Density 3D Culture

High density 3D cultures were obtained using 96-well ultra-low attachment microplates (Corning, Manassas, VA, USA). Briefly, MeWo cells were resuspended at a density of 500.000 cells/mL. Then, 100 μL of cell suspension were dispensed in each well and, after 3 days, the resulting cellular aggregates were treated with different metallic complexes at 5 μm for 24 h and 48 h. For the cell viability assay, the average value of 10 3D-systems (*n* = 3) were considered.

### 3.11. Optical Microscope Analyses

Brightfield images of spheroids were captured at different time points at 5× magnification using a Leica DMIL LED microscope (Leica Microsystems S.r.l., Buccinasco, Italy) and acquired by Leica DFC425 C Camera (Leica Microsystems S.r.l., Buccinasco, Italy).

### 3.12. Cell Viability Assay

Cell viability of 3D cultures was detected by fluorescence Live/Dead assay after cellular aggregate formation. Calcein AM solution (Cat. no C1359, Sigma-Aldrich) was used to stain live cells, while cell membrane-impermeable Ethidium homodimer I solution (Cat. no E1903, Sigma-Aldrich) for nuclei of dead cells. Spheroids were incubated for 1 h at 37 °C, then washed in 1X PBS, and imaged in a fluorescence microscope (Eclipse Ti Nikon Corporation, Tokyo, Japan).

Cell viability of 3D cultures was also determined by an adenosine-5′-triphosphate (ATP) assay with the Promega CellTiter-Glo^®^ 3D Cell Viability kit (Promega Italia S.r.l., Milan, Italy). Relative Luminescence units (RLUs) were recorded with a LuMate^®^ luminometer (Awareness Technology Inc., Palm City, FL, USA) and the resulting data were analyzed through LuMate Manager software (version 2.0).

### 3.13. Statistical Analysis

Results from multiple experiments are presented as mean  ±  standard deviation (SD). Statistical analysis was performed using ordinary one-way analysis of variance (ANOVA) test for independent groups, after checking data normality and homogeneity. *p* values less than 0.05 were accepted as significant [53,54,55,56]. All statistical analysis was conducted using GraphPad Prism software (6.0 for Windows, LLC, San Diego, CA, USA).

## 4. Conclusions

New complexes were investigated for their activity against MeWo proliferation and growth. AuM1 and PheAg complexes resulted as the most effective in inhibiting the viability of cancer cells and quite selective on normal cells. AuM1, AuM1Phe, and PheAg complexes tested on 3D MeWo culture induced significant cell disaggregation after 24 h of treatment. In general, metal complexes are reported to act through inducing oxidative stress and DNA lesions leading to cell apoptosis and cell death [57], and this concept has been confirmed also in the case of Ag and Au–based complexes here tested. In addition, few studies in vitro and in silico suggested that these complexes were able to inhibit the activity of the human topoisomerases I and II and the actin polymerization reaction. Moreover, a downregulation of vimentin expression and a reduced translocation of NF-kB into the nucleus was observed [58]. However, after the proposed screening on the most promising complex derivatives, further studies will be required to better investigate the biochemical pathways of molecular toxicity in both in vitro and in vivo models. From our data, it also emerged that these complexes have a selectivity for cancer cells compared to normal cells, even if the uptake mechanism is largely unknown. Furthermore, when a MeWo 3D culture has been adopted, the complex availability through diffusion may be prevented by mass transport resistance provided by cells aggregation; in this sense, an optimized drug delivery system may improve the complex uptake by the 3D aggregates.

However, the study elucidated the considerable potential of such chemical complexes as novel agents able to specifically target cisplatin-resistant melanoma and opens interesting perspectives for specific studies on the AuM1, AuM1Phe, and PheAg complex activity.

## Figures and Tables

**Table 1:** Resonances of carbene carbon of NHC-metal complexes.

M-NHC	*δ* _C-M_ DMSO (ppm)
AgL20	181.2
AgL20-Gly	178.4
AgL20 Phe	178.4
AgM1	181.6
AgM1-Gly	180.7
AgM1-Phe	180.7
AuL20	172.0
AuL20-Gly	164.5
AuL20-Phe	164.4
AuM1	170.7
AuM1-Gly	163.2
AuM1-Phe	163.3

**Table 2:** IC_50_ values for silver and gold complexes tested in this work.

M-NHC	IC_50_ 24 h (μM)	IC_50_ 48 h (μM)
AgL20	~19.45	18.34
AuL20	~24.63	31.14
AgM1	15.40	14.85
AuM1	5.48	3.12

## Data Availability

Not applicable.

## Supplementary Materials

The following supporting information can be downloaded at: https://www.mdpi.com/article/10.3390/molecules28124851/s1. The NMR data set and information.

## Sample Availability

Synthesized molecules are available.
