# Supplementary material for: Activity and Selectivity of Novel Chemical Metallic Complexes with Potential Anticancer Effects on Melanoma Cells
Source: Molecules. 2023 Jun 19;28(12):4851. doi: 10.3390/molecules28124851 (PMC10303371; doi:10.3390/molecules28124851)
Supplement: Supplementary file 1 [file molecules-28-04851-s001.zip › molecules-2372574-supplementary.pdf]

Activity and Selectivity of Novel Chemical Metallic Complexes with Potential Anticancer Effects on Melanoma Cells

Maria Camilla Ciardulli [1,†], Annaluisa Mariconda [2,†], Marco Sirignano [3], Erwin Pavel Lamparelli [1], Raffaele Longo [4], Pasqualina Scala [1], Raffaella D'Auria [1], Antonietta Santoro [1,5], Liberata Guadagno [4], Giovanna Della Porta [1,5,*] and Pasquale Longo [3]

[1] Department of Medicine, Surgery and Dentistry, University of Salerno, Via S. Allende, 84081 Baronissi, Italy; mciardulli@unisa.it (M.C.C.); elamparelli@unisa.it (E.P.L.); pscala@unisa.it (P.S.); radauria@unisa.it (R.D.); ansantoro@unisa.it (A.S.)
[2] Department of Science, University of Basilicata, Viale dell'Ateneo Lucano 10, 85100 Potenza, Italy;
annaluisa.mariconda@unibas.it
[3] Department of Chemistry and Biology "Adolfo Zambelli", University of Salerno, Via Giovanni Paolo II, 132 84084 Fisciano, Italy; msirignano@unisa.it (M.S.); plongo@unisa.it (P.L.)
[4] Department of Industrial Engineering, University of Salerno, Via Giovanni Paolo II, 132 84084 Fisciano, Italy; rlongo@unisa.it (R.L.); lguadagno@unisa.it (L.G.)
[5] Interdepartment Centre BIONAM, University of Salerno, Via Giovanni Paolo II, 84084 Fisciano, Italy
* Correspondence: gdellaporta@unisa.it; Tel.: +39-089965234.
† These authors contributed equally to this work.

List of contents

^{1}H-NMR GlyAg S1

^{13}C-NMR GlyAg S2

MALDI-MS GlyAg S3

^{1}H-NMR PheAg S4

^{13}C-NMR PheAg S5

MALDI PheAg S6

^{1}H-NMR AgL20Gly S7

^{13}C-NMR AgL20Gly S8

MALDI AgL20Gly S9

^{1}H-NMR AgL20Phe S10

^{13}C-NMR AgL20Phe S11

MALDI AgL20Phe S12

^{1}H-NMR AgM1Gly S13

^{13}C-NMR AgM1Gly S14

MALDI AgM1Gly S15

^{1}H-NMR AgM1Phe S16

^{13}C-NMR AgM1Phe S17

MALDI AgM1Phe S18

^{1}H-NMR AuL20Gly S19

^{13}C-NMR AuL20Gly S20

MALDI AuL20Gly S21

^{1}H-NMR AuL20Phe S22

^{13}C-NMR AuL20Phe S23

MALDI AuL20Phe S24

^{1}H-NMR AuM1Gly S25

^{13}C-NMR AuM1Gly S26

MALDI AuM1Gly S27

^{1}H-NMR AuM1Phe S28

^{13}C-NMR AuM1Phe S29

MALDI AuM1Phe S30

Thermal stability of AgM1Gly S34

Hydrolytic Stability of AgM1Gly, AgPhe, AuM1 and AuM1Phe S35

FT-IR Characterization........ S31

IC_{50} values S38

¹H-NMR GlyAg

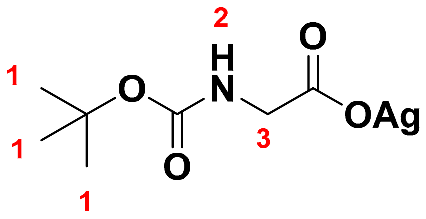

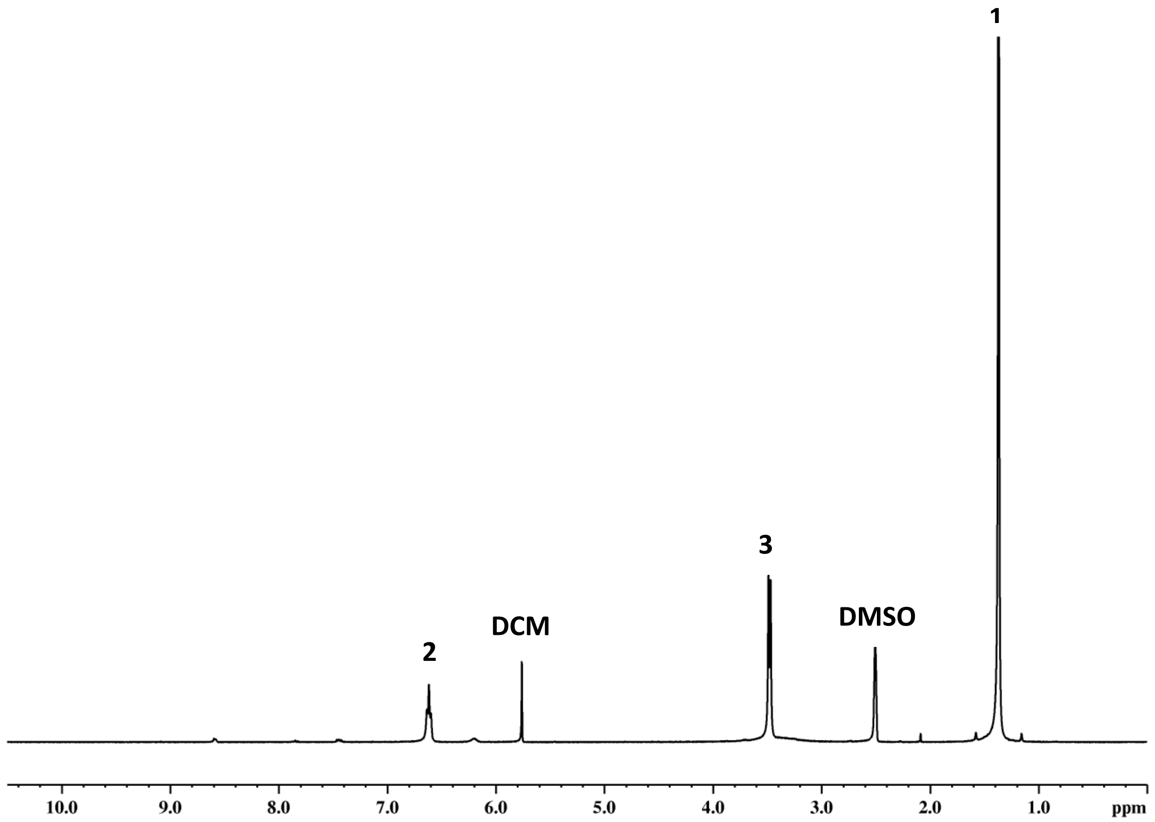

¹H-NMR (300 MHz, DMSO-d_6, ppm): δ 6.34 (t, J_{vic}=5.2 Hz, 1H, N**H**); 3.41 (d, J_{vic}=5.2 Hz, 2H, NHC**H**$_2$); 1.36 (s, 9H, C(C**H**$_3$)$_3$).

^{13}C-NMR GlyAg

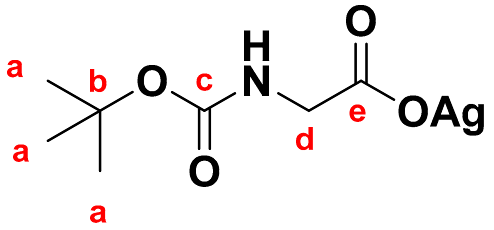

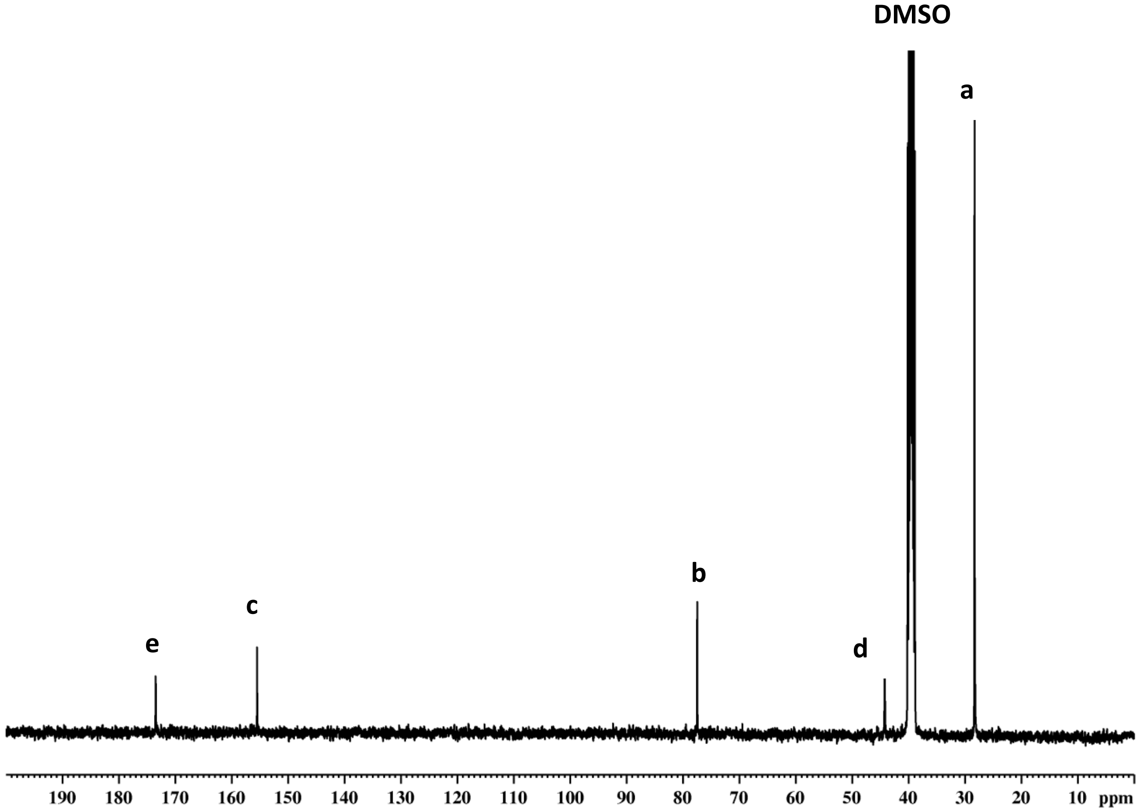

^{13}C-NMR (75 MHz, DMSO-d_6 ppm): δ 173.4 (**C**OOAg); 155.4 (NH**C**O); 77.4 (**C**(CH_3)$_3$); 44.2 (NH**C**H_2); 28.2 (C(**C**H_3)$_{3}$,).

MALDI-MS GlyAg

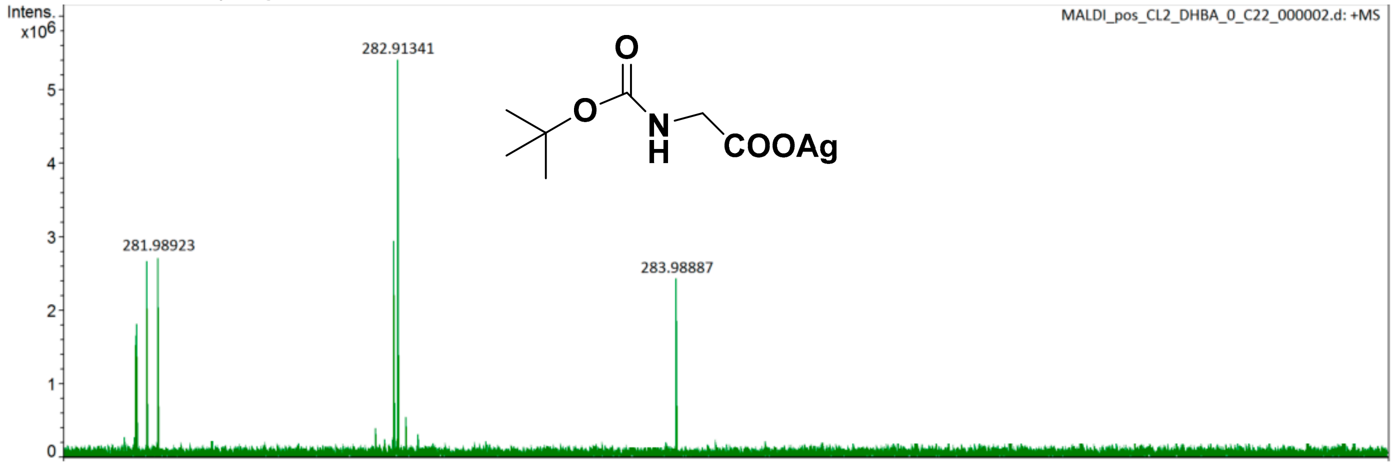

MALDI-MS (m/z): 282.911341 attributable to $[C_7H_{12}AgNO_4]^+$

Elemental Analysis: theorical = C: 29.81, H 4.29, Ag 38.25, N 4.97, O 22.69; experimental= C 29.75, H 4.30, Ag 38.30, N 5.00, O 22.66

^{1}H-NMR PheAg

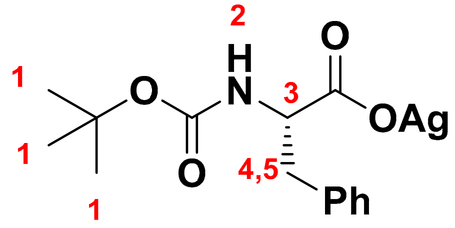

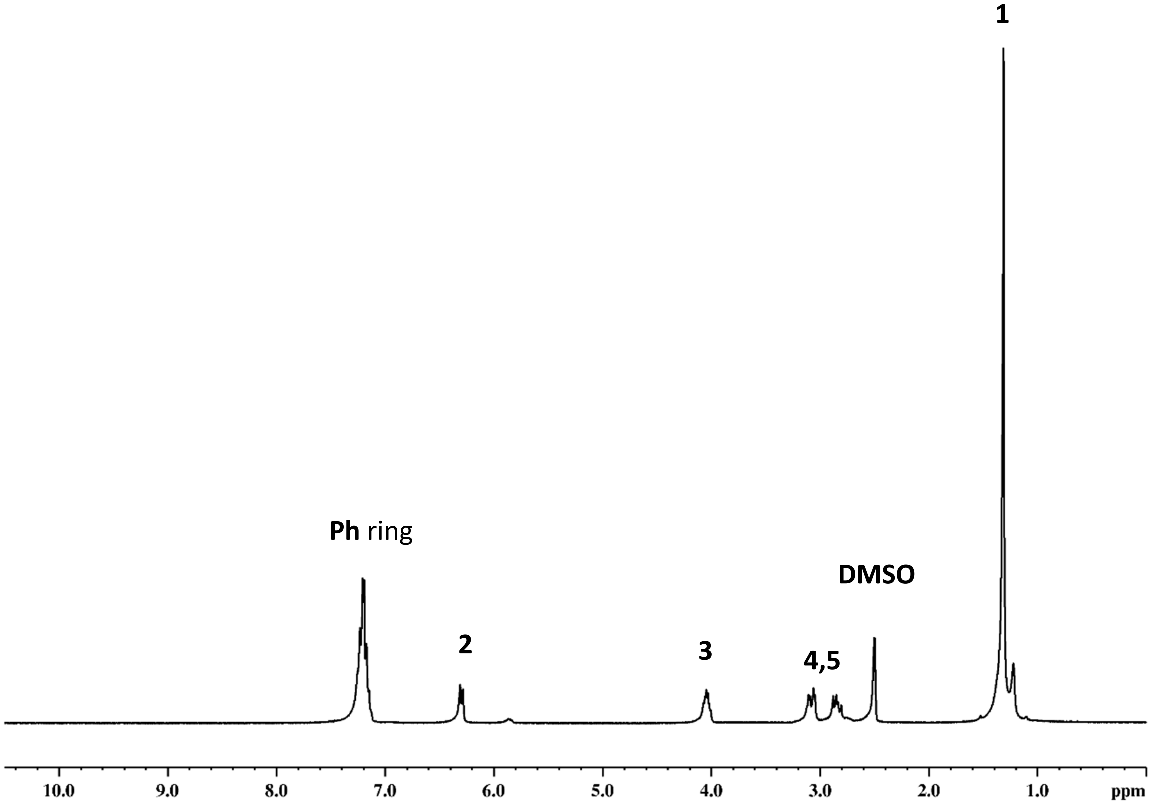

^{1}H-NMR (300 MHz DMSO-d_6, ppm): δ 7.20 (m, 5H, **Ph** ring); 6.32 (d, J_{vic}=8.0 Hz, 1H, N**H**); 4.05 (m, J_{vic}=8.5, 8.1, 4.5 Hz, 1H, C**H**); 3.10(dd, J_{gem}=14.0 Hz, J_{vic}= 8.5Hz, 1H, C**H**$_2$Ph); 2.86 (dd, J_{gem}=14.0, J_{vic}= 8.1 Hz 1H, C**H**$_2$Ph); 1.31 (s, 9H, C(C**H**$_3$)$_3$).

^{13}C-NMR PheAg

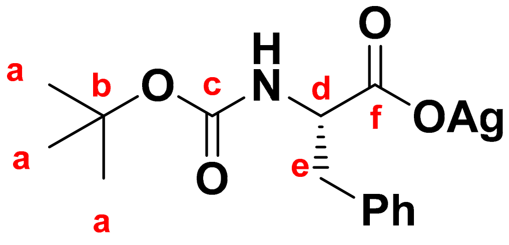

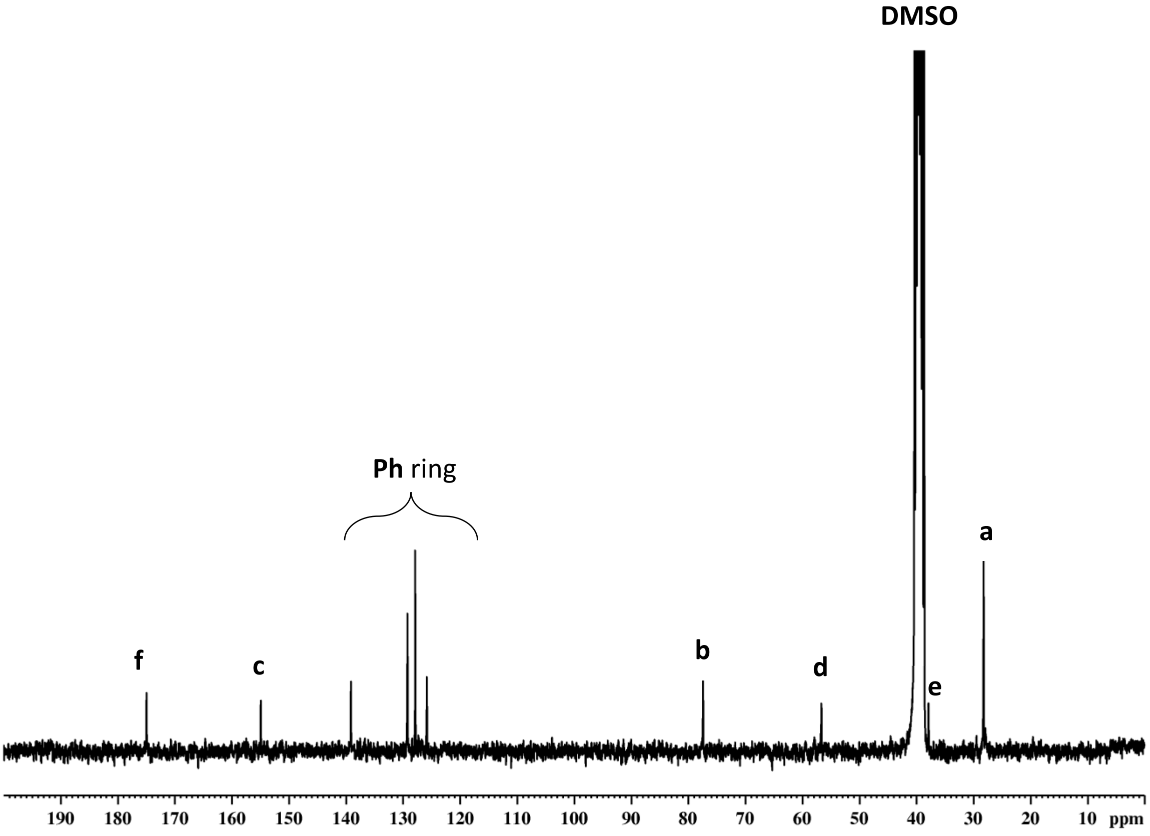

^{13}C-NMR (100 MHz DMSO-d$_6$, ppm): δ 174.9 (**C**OOAg); 154.9 (NH**C**O); 139.1, 129.2, 127.8, 125.8 (**Ph** ring); 77.4 (**C**(CH$_3$)$_3$); 56.6 (NH**C**H); 36.4 (Ph**C**H$_2$); 28.2 (C(**C**H$_3$)$_3$).

MALDI PheAg

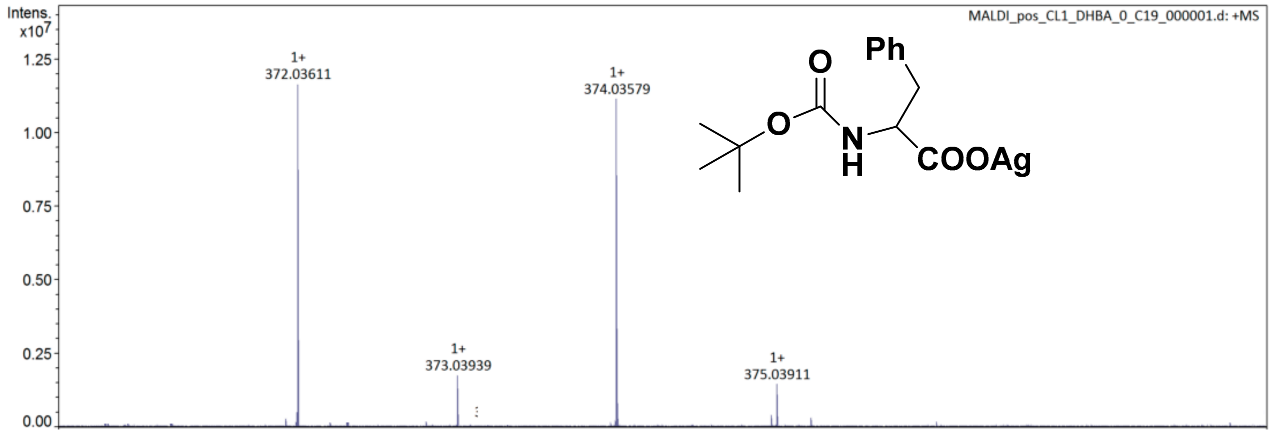

MALDI-MS **(m/z):** 372.03611 attributable to $[C_{14}H_{18}AgNO_4]^+$

Elemental Analysis: theorical = C: 45.15, H 4.83, Ag 29.06, N 3.76, O 17.20; experimental= C 45.10, H 4.88, Ag 29.10, N 3.80, O 17.12

^{1}H-NMR AgL20Gly

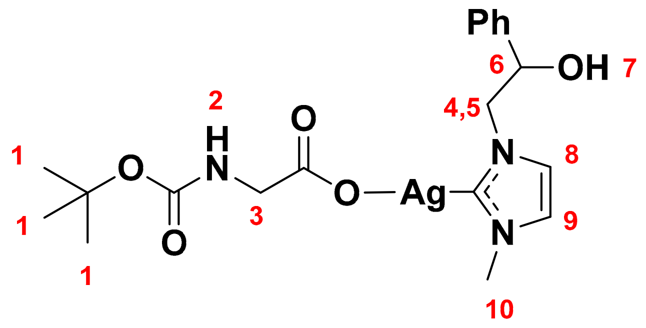

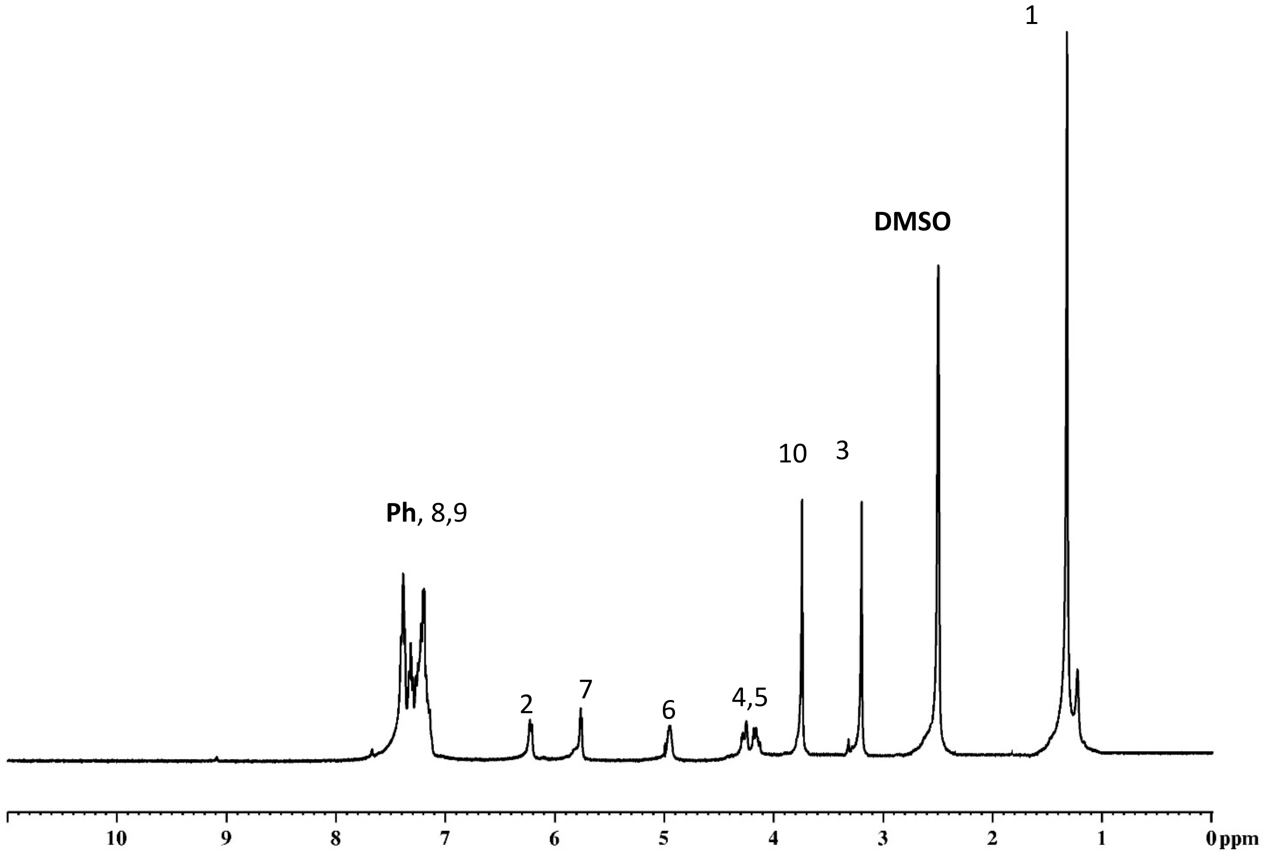

^{1}H-NMR (400 MHz, DMSO-d$_6$, ppm): δ 7.35-7.33 (m, 7H, C_6**H_5**CH(OH)CH_2NC**H**C**H**N); 6.37 (t, J_{vic}=5.0 Hz, 1H, Gly: N**H**); 5.85 (d, 1H, C_6H_5CH(**OH**)CH_2N), 5.01 (m, J_{vic}=10.3, 8.4 Hz, 1H, C_6H_5C**H**(OH)CH_2N); 4.41 (m, J_{gem}=15.0 Hz, J_{vic}=10.3, 8.4 Hz, 2H, C_6H_5CH(OH)C**H_2**N); 3.90 (s, 3H, NC**H_3**); 3.51 (d, J_{gem}=11.0 Hz, J_{vic}=5.0 Hz, 2H, Gly: NHC**H_2**); 1.29 (s, 9H, Gly: C(C**H_3**)$_3$).

^{13}C-NMR AgL20Gly

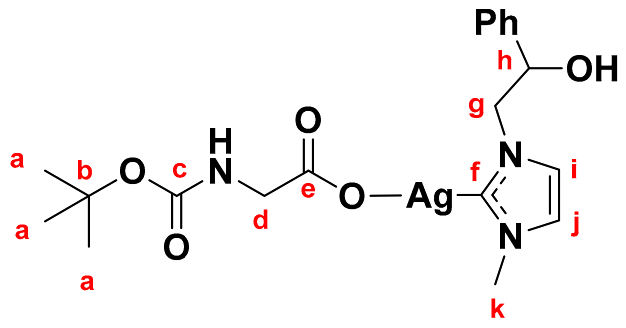

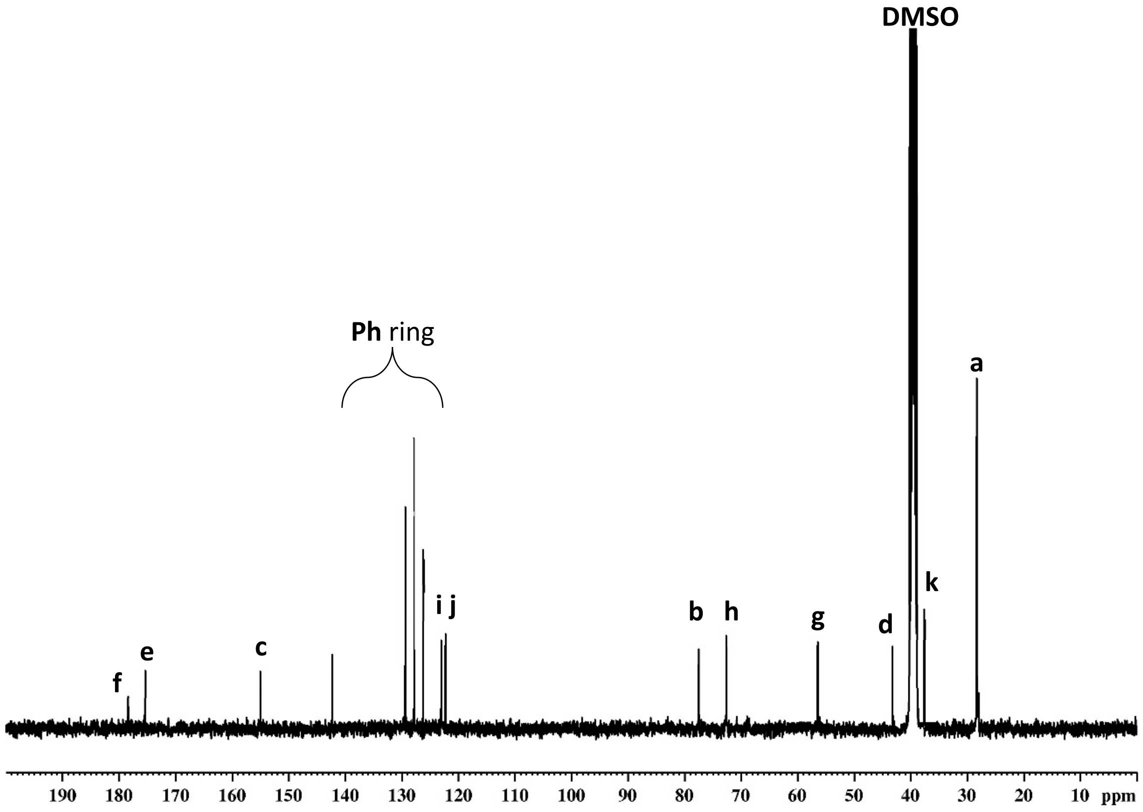

^{13}C-NMR (100 MHz, DMSO-d_6, ppm): δ 178.4 (N**C**N); 175.4 (**C**OOAg); 154.9 (NH**C**O); 142.2, 139.2, 128.2, 127.5, 125.8 (**Ph ring**); 122.9, 122.2 (N**C**H**C**HN); 77.4 (**C**(CH_3)$_3$); 72.6 (Ph**C**HOH); 58.2 (N**C**H_2); 45.2 (NH**C**H_2); 38.1 (N**C**H_3); 28.2 (C(**C**H_3)$_3$).

MALDI AgL20Gly

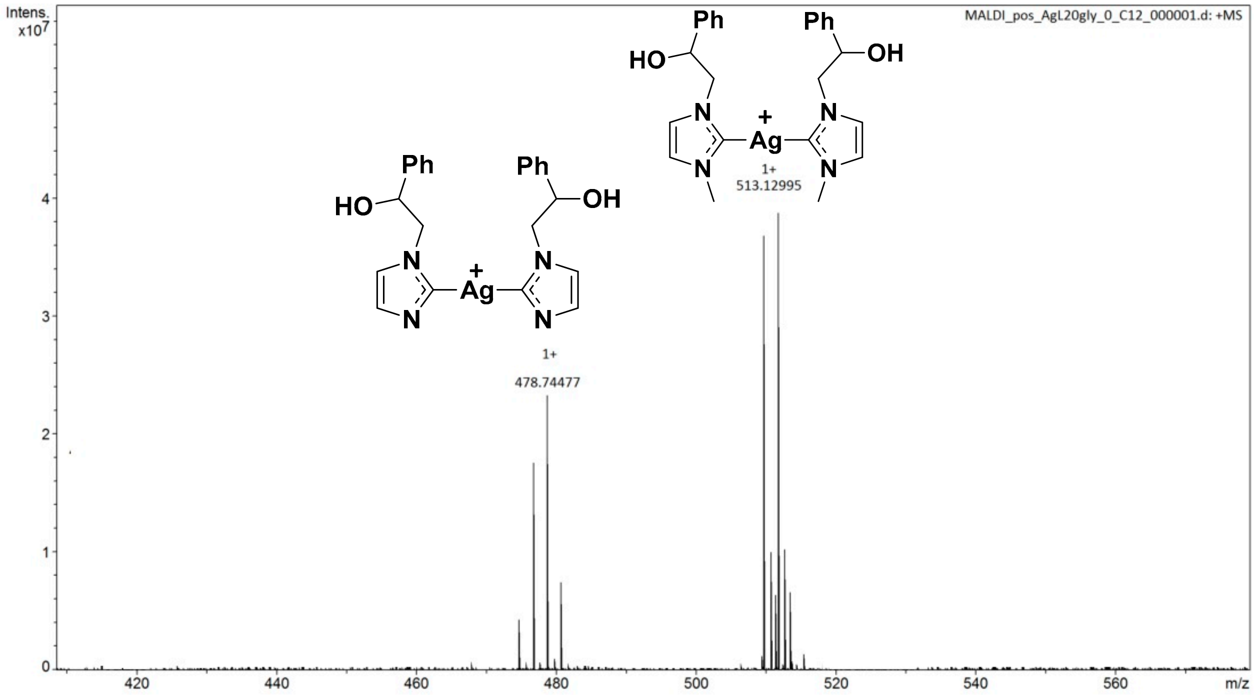

MALDI-MS (m/z): 513.12995 attributable to bis-carbene structure $[C_{24}H_{28}AgN_4O_2]^+$ and 478.74477 attributable to $[C_{22}H_{22}AgN_4O_2]^+$

Elemental Analysis: theorical = C: 47.12, H 5.41, Ag 22.27, N 8.68, O 16.52; experimental= C 47.08, H 5.45, Ag 22.17, N 8.78, O 16.52

^{1}H-NMR AgL20Phe

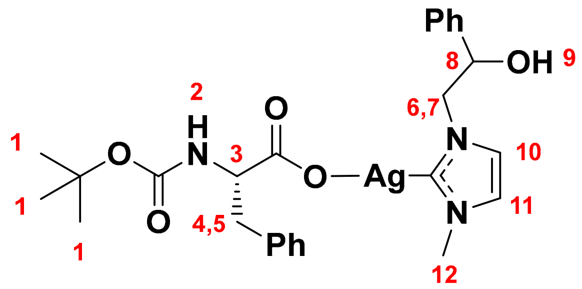

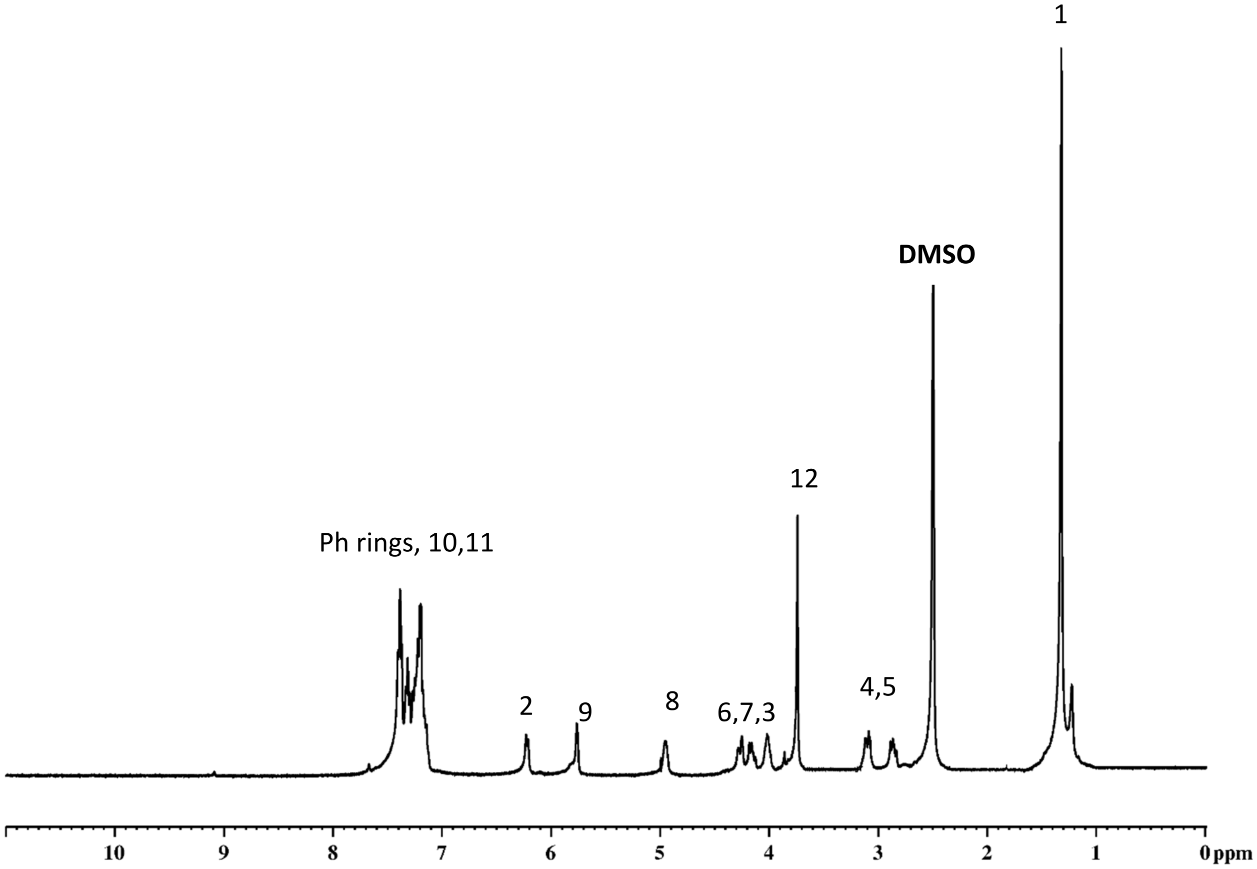

^{1}H-NMR (400 MHz, DMSO-d_6, ppm): δ 7.40-7.17 (m, 12 H, $C_6\mathbf{H}_5$CH(OH)CH_2NC**H**C**H**N+Phe:$C_6\mathbf{H}_5$); 6.20 (t, J_{vic}=8.0 Hz, 1H, Phe: N**H**); 5.77 (d, 1H, C_6H_5CH(O**H**)CH_2N); 4.95 (m, J_{vic}=10.5 , 8.7 Hz, 1H, C_6H_5C**H**(OH)CH_2N); 4.26, 4.16 (dd, J_{gem}= 15.6 Hz, J_{vic}= 10.5, 8.7 Hz, 2H, C_6H_5CH(OH)C$\mathbf{H}_2$N); 4.01 (dd, J_{vic}=9.0, 8.5 Hz 1H, Phe: C**H**CH_2Ph); 3.74 (s, 3H, NC$\mathbf{H}_3$); 3.09, 2.88 (dd, J_{gem}= 14.0 Hz, J_{vic}=9.0, 8.5 Hz, 2H, Phe: CHC$\mathbf{H}_2$Ph); 1.39 (s, 9H, Phe: C(C$\mathbf{H}_3$)$_3$).

^{13}C-NMR AgL20Phe

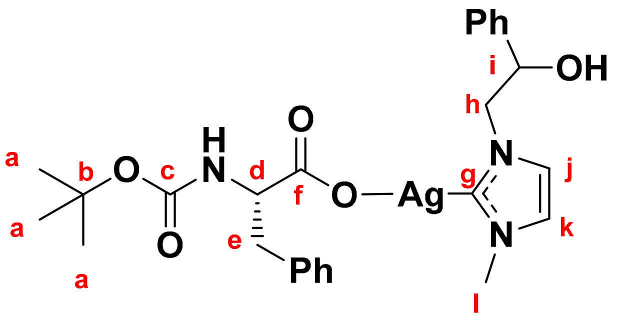

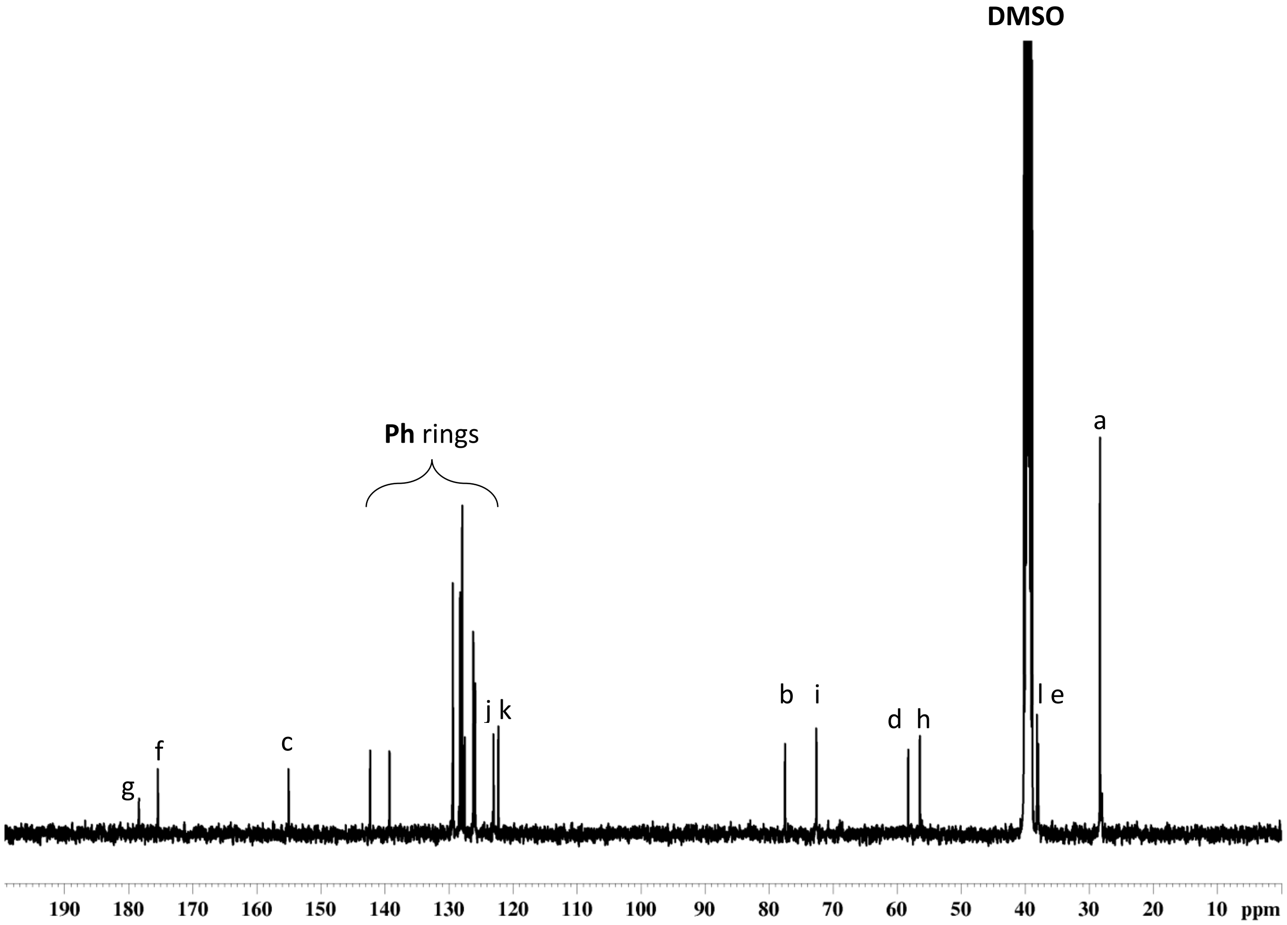

^{13}C-NMR (100 MHz, DMSO-d$_6$, ppm): δ 178.4 (N**C**N); 175.4 (**C**OOAg); 154.9 (NH**C**O); 142.2; 139.2, 129.3, 128.2, 127.8, 127.5, 126.1, 125.8 (**Ph** rings); 124.2, 122.9 (N**C**H**C**HN); 77.4 (**C**(CH$_3$)$_3$); 72.5 (Ph**C**HOH); 58.2 (N**C**H$_2$); 56.4 (NH**C**H); 38.1 (N**C**H$_3$); 37.9 (CH**C**H$_2$Ph); 28.3(C(**C**H$_3$)$_3$).

MALDI AgL20Phe

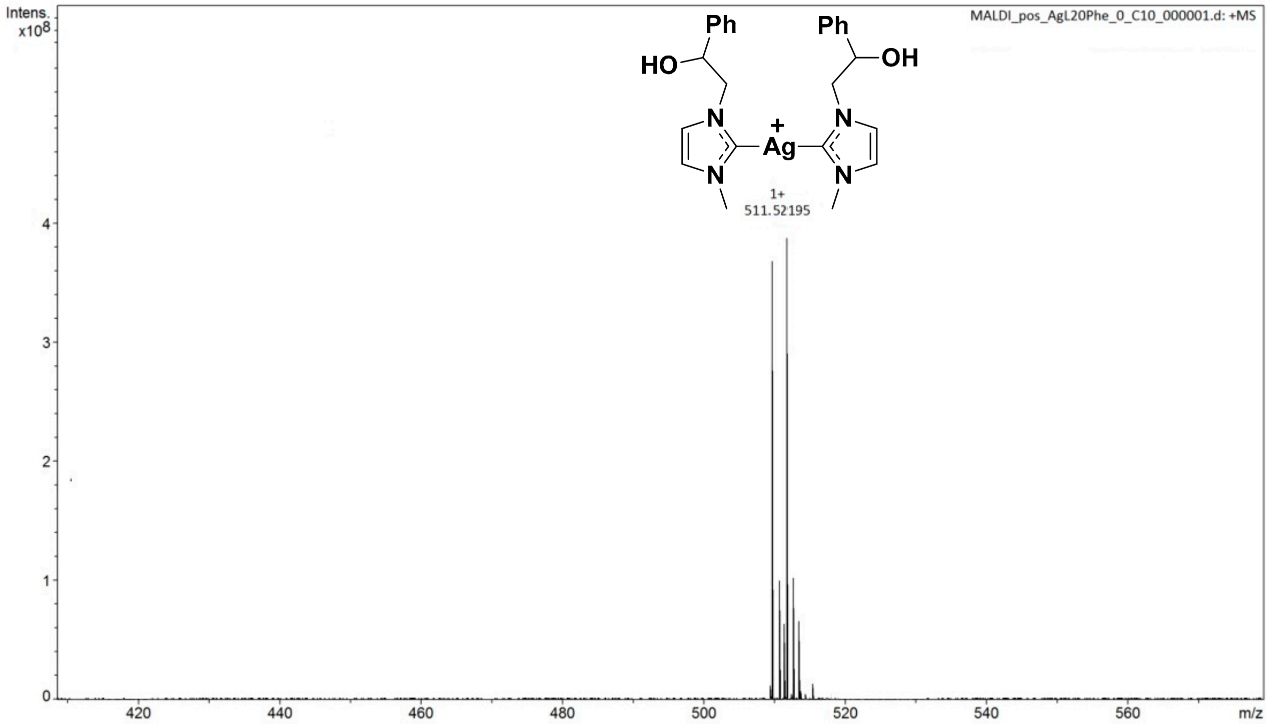

MALDI-MS (m/z): 511.52195 attributable to bis-carbene structure $[C_{24}H_{28}AgN_4O_2]^+$

Elemental Analysis: theorical = C: 54.36, H 5.62, Ag 18.78, N 7.32, O 13.92; experimental = C 54.30, H 5.68, Ag 18.75, N 7.35, O 13.92

^{1}H-NMR AgM1Gly

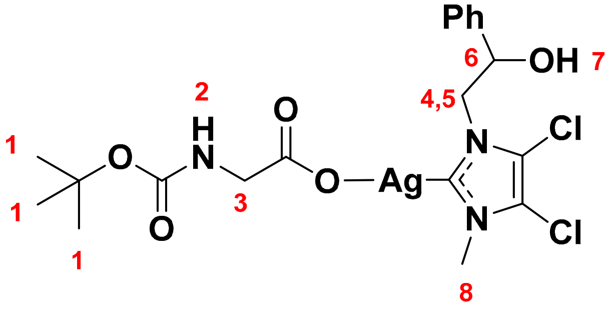

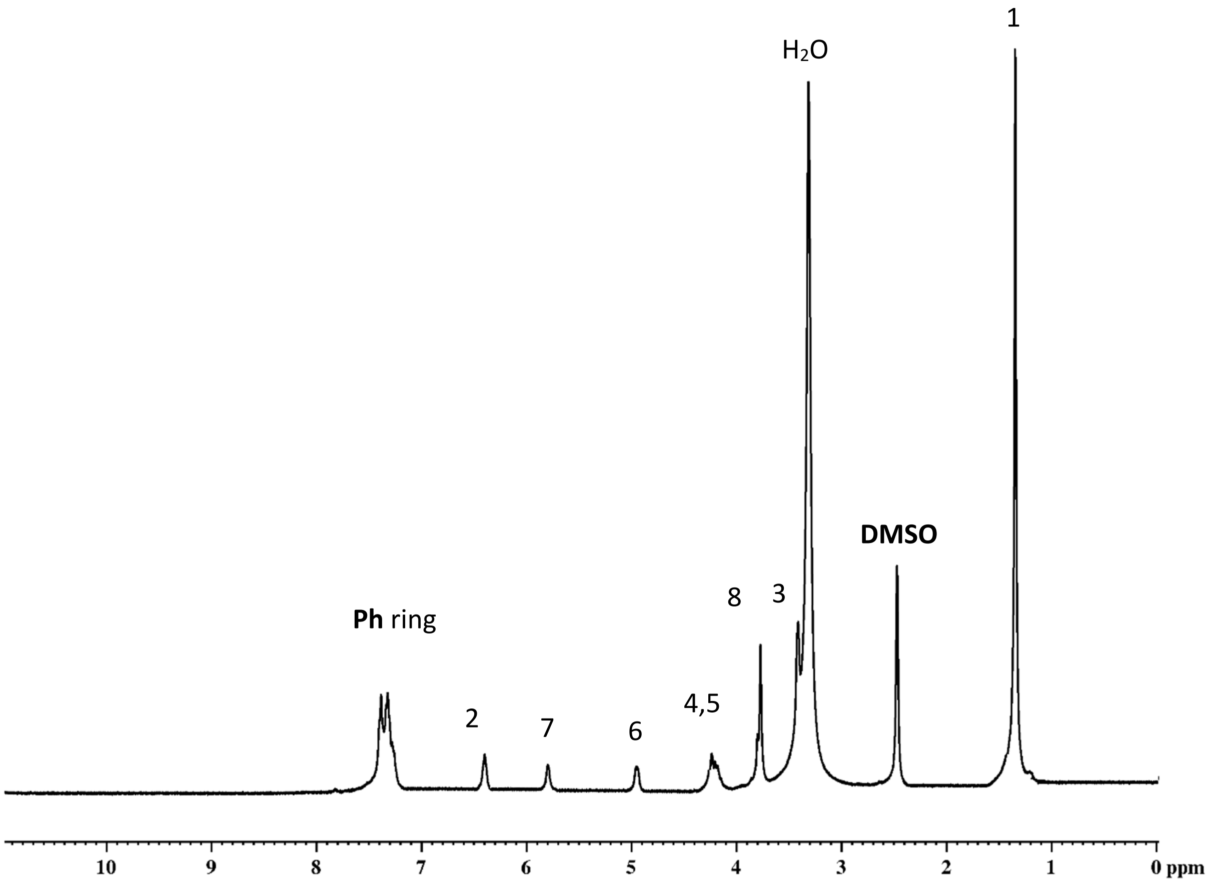

^{1}H-NMR (400 MHz, DMSO-d$_6$, ppm): δ 7.37-7.35 (m, 5H, $C_6\mathbf{H}_5CH(OH)CH_2N$); 6.34 (t, J_{vic}=5.6 Hz, 1H, Gly: N**H**); 5.97 (s, 1H, $C_6H_5CH(O\mathbf{H})CH_2N$); 4.97 (m, J_{vic}= 10.7, 8.6 Hz, 1H, $C_6H_5C\mathbf{H}(OH)CH_2N$); 4.25 (m, J_{gem}=13.8 Hz, J_{vic}=10.7, 8.6 Hz, 2H, $C_6H_5CH(OH)C\mathbf{H}_2N$); 3.81 (s, 3H, $NC\mathbf{H}_3$); 3.25 (s, 2H, Gly: $NHC\mathbf{H}_2$); 1.36 (s, 9H, Gly: $C(C\mathbf{H}_3)_3$).

^{13}C-NMR AgM1Gly

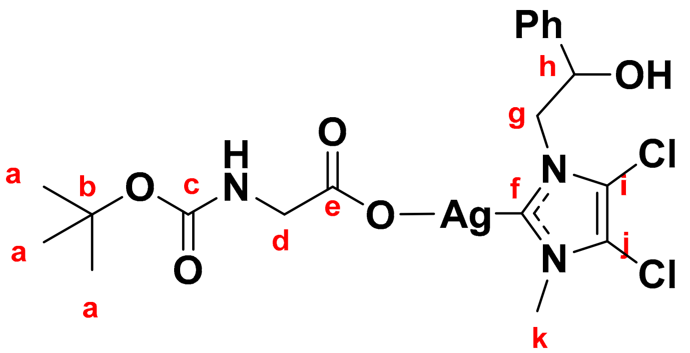

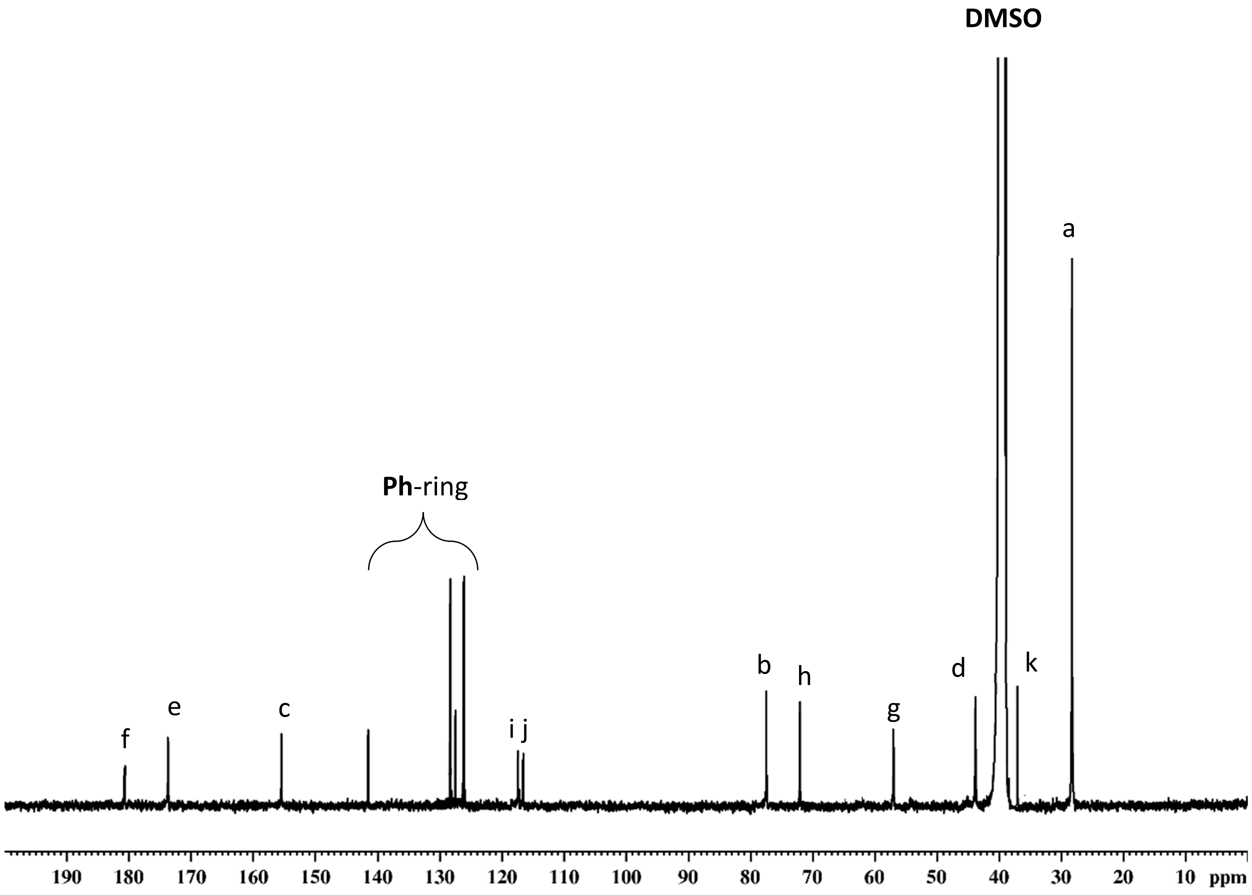

^{13}C-NMR (100 MHz, DMSO-d$_6$, ppm): δ: 180.7 (N**C**N); 173.7 (**C**OOAg); 155.4 (NH**C**O); 141.5, 128.8, 127.7, 126.1 (**Ph** ring); 117.4, 116.5 (N**C**Cl**C**ClN); 77.4 (**C**(CH$_3$)$_3$); 72.0 (C**H**OH); 56.9 (N**C**H$_2$); 43.7 (NH**C**H$_2$); 37.6 (N**C**H$_3$); 28.2 (C(**C**H$_3$)$_3$).

MALDI AgM1Gly

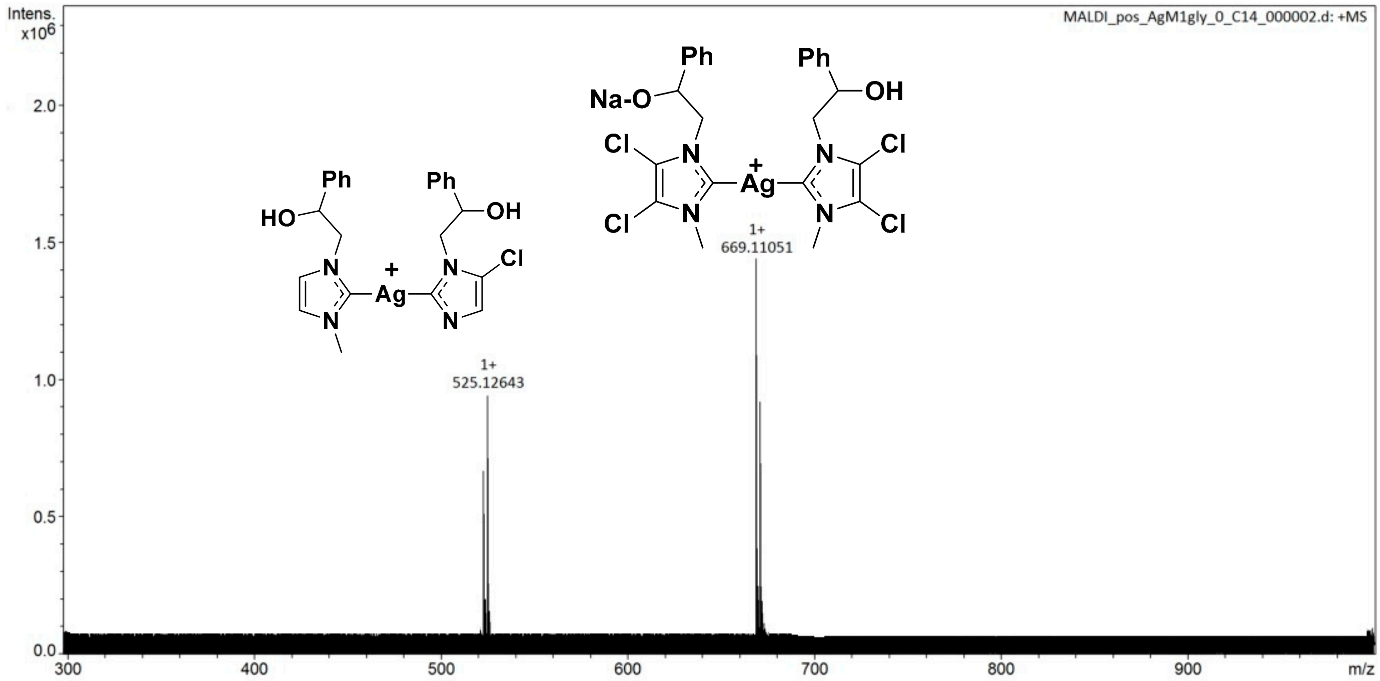

MALDI-MS (m/z): 669.11051 attributable to bis-carbene structure $[C_{24}H_{23}AgCl_4N_4NaO_2]^+$ and 525.12643 attributable to $[C_{23}H_{21}AgClN_4O_2]^+$

Elemental Analysis: theorical = C: 41.25, H 4.37, Ag 19.50, Cl 12.82, N 7.60, O 14.46; experimental= C 41.22, H 4.31, Ag 19.49, Cl 12.82, N 7.70, O 14.46

^{1}H-NMR AgM1Phe

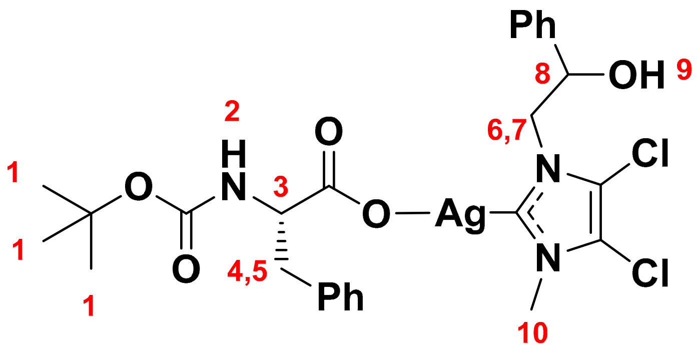

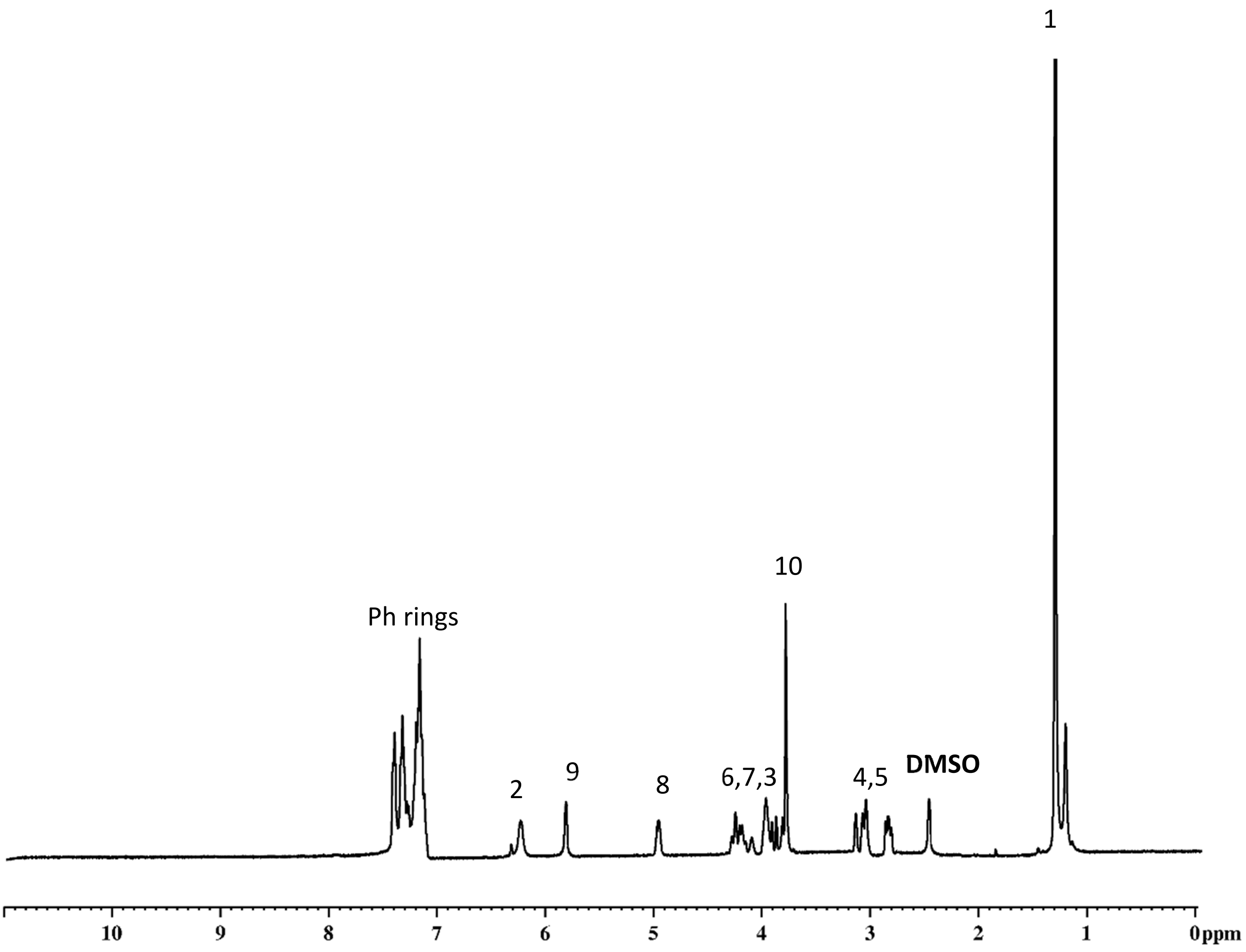

^{1}H-NMR (400 MHz, DMSO-d_6, ppm): δ 7.43-7.20 (m, 10H, $C_6\mathbf{H}_5CH(OH)CH_2N$+Phe:$C_6\mathbf{H}_5$); 6.30 (m, J_{vic}=8.6 Hz, 1H, Phe: N**H**); 5.84 (d, 1H, $C_6H_5CH(O\mathbf{H})CH_2N$); 4.99 (m, J_{vic}=10.8, 8.6 Hz, 1H, $C_6H_5C\mathbf{H}(OH)CH_2N$); 4.27 (m, 2H, $C_6H_5CH(OH)C\mathbf{H}_2N$); 4.04 (m, 1H, Phe: C**H**CH_2Ph); 3.80 (s, 3H, $NC\mathbf{H}_3$); 3.11-2.87 (dd, 2H, Phe: $CHC\mathbf{H}_2$Ph); 1.31 (s, 9H, Phe: $C(C\mathbf{H}_3)_3$).

^{13}C-NMR AgM1Phe

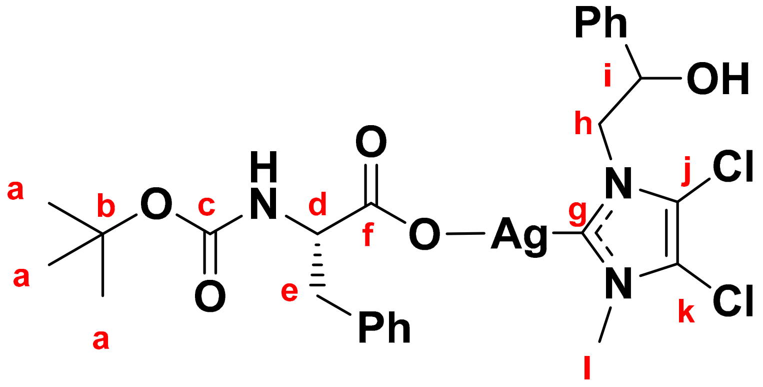

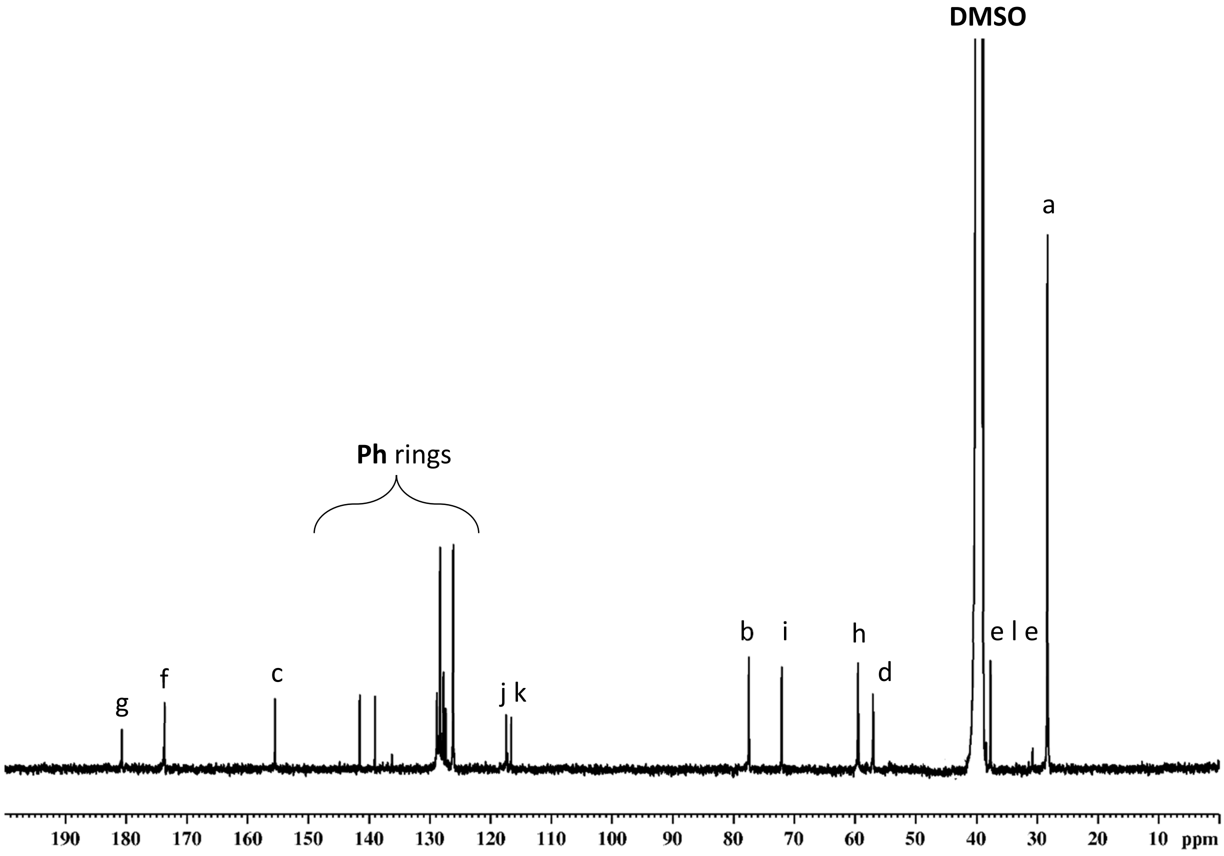

^{13}C-NMR (100 MHz, DMSO-d_6, ppm): δ 180.7 (N**C**N); 175.3 (**C**OOAg); 155.0 (NH**C**O); 141.5, 139.0, 129.2, 128.8, 128.3, 127.9, 126.1, 125.9 (**Ph** rings); 117.4, 116.6 (N**C**Cl**C**ClN); 77.4 (**C**(CH_3)$_3$); 72.8 (**C**HOH); 57.0 (N**C**H_2); 56.2 (**C**HNH); 37.7 (N**C**H_3); 37.0 (**C**H_2Ph); 28.1 (C(**C**H_3)$_3$).

MALDI AgM1Phe

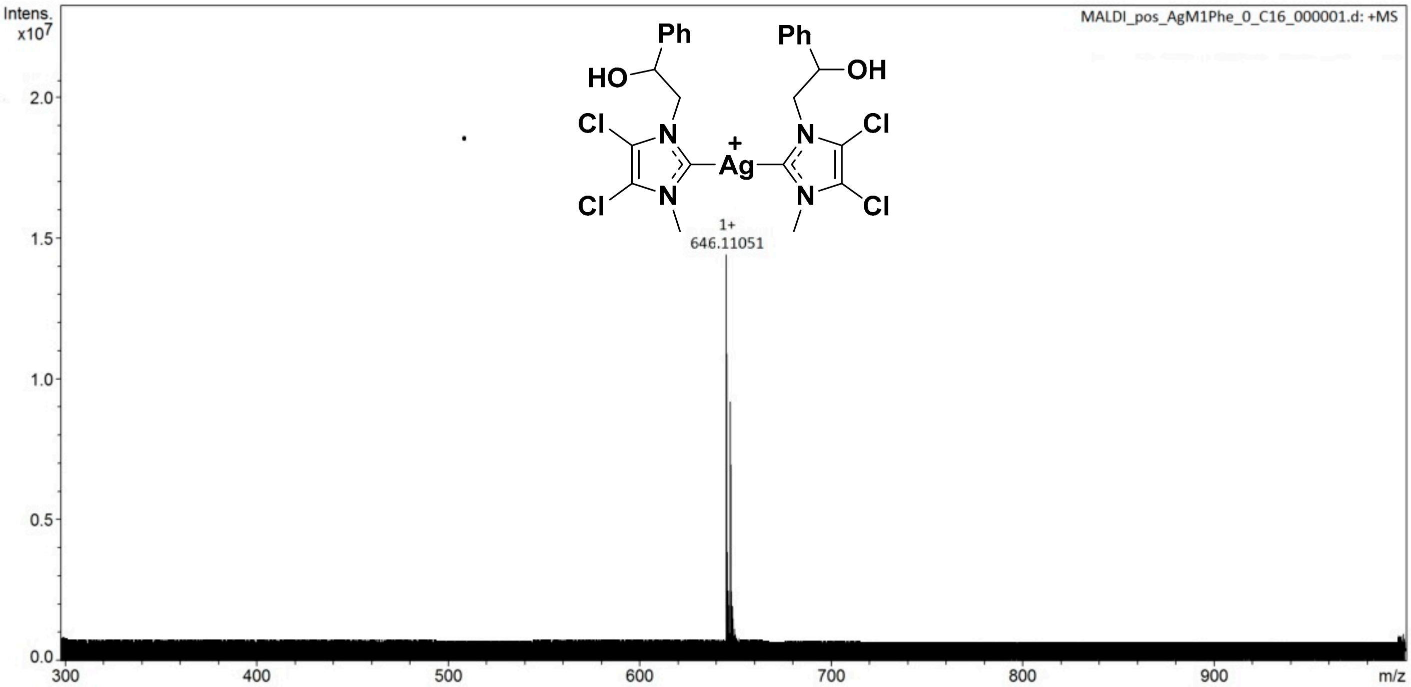

MALDI-MS (m/z): 646.11051 attributable to bis-carbene structure $[C_{24}H_{24}AgCl_4N_4O_2]^+$

Elemental Analysis: theorical = C: 48.54, H 4.70, Ag 16.77, Cl 11.02, N 6.53, O 12.43; experimental= C 48.55, H 4.80, Ag 16.71, Cl 11.02, N 6.55, O 12.37

$[\alpha]_D^{25}(C = 0.37)$: -99.86

^{1}H-NMR AuL20Gly

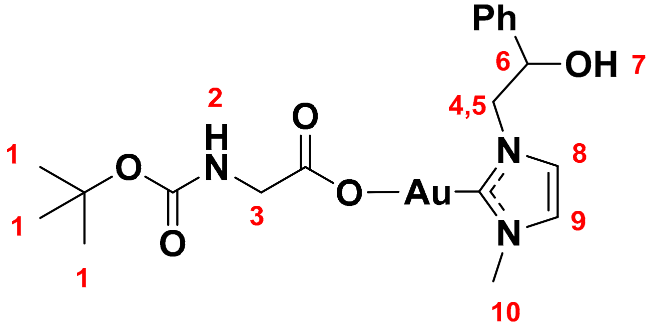

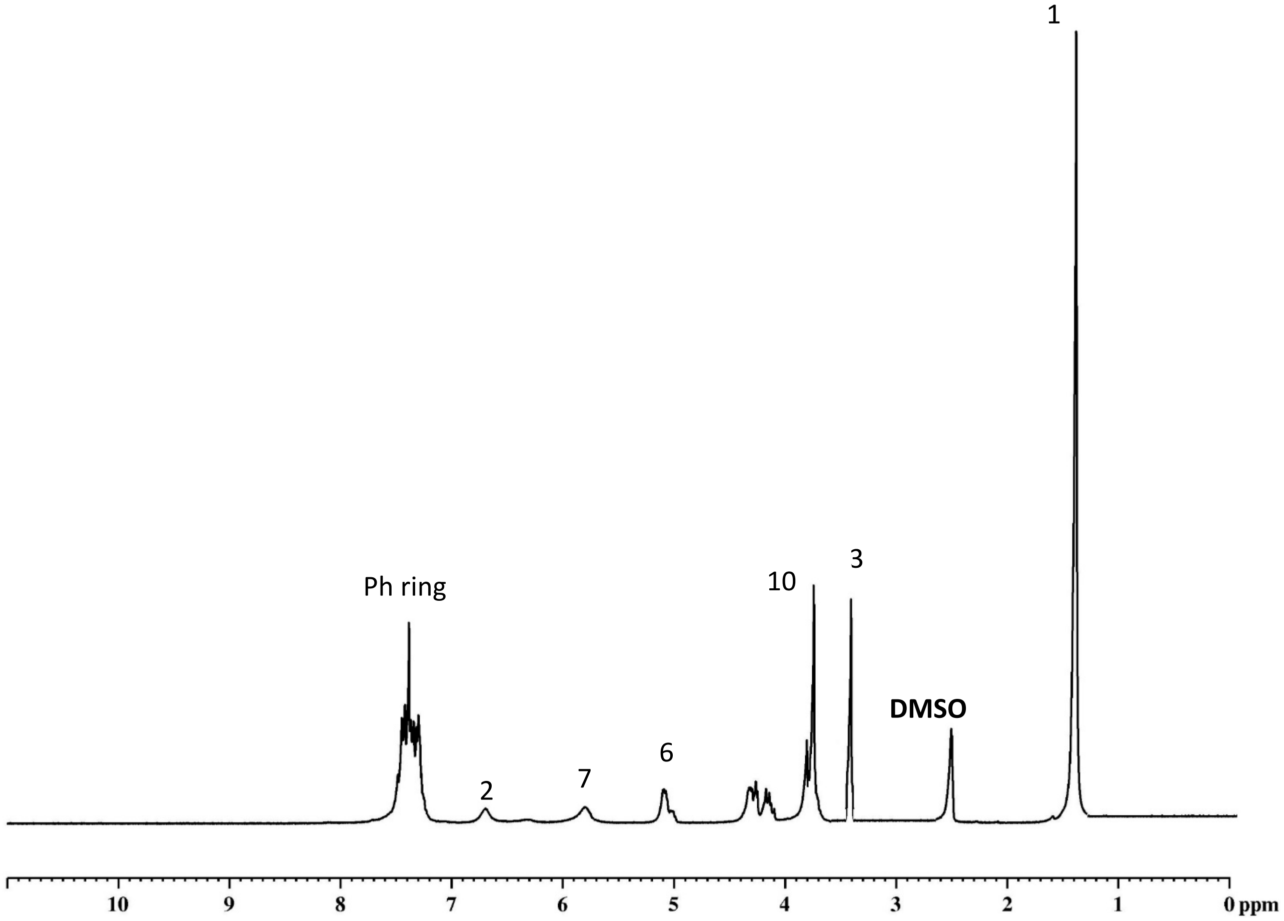

^{1}H-NMR (400 MHz, DMSO-d$_6$, ppm): δ 7.35-7.33, (m, 7H, $C_6\mathbf{H}_5CH(OH)CH_2NC\mathbf{H}C\mathbf{H}N$); 6.87 (br, 1H, Gly: N**H**); 5.85 (br, 1H, $C_6H_5CH(O\mathbf{H})CH_2N$), 5.01 (m, 1H, $C_6H_5C\mathbf{H}(OH)CH_2N$); 4.41 (m 2H, $C_6H_5CH(OH)C\mathbf{H}_2N$); 3.90 (s, 3H, $NC\mathbf{H}_3$); 3.51 (d, J_{gem}=11.0 Hz, J_{vic}=5.0 Hz, 2H, Gly: $NHC\mathbf{H}_2$); 1.29 (s, 9H, Gly: $C(C\mathbf{H}_3)_3$).

^{13}C-NMR AuL20Gly

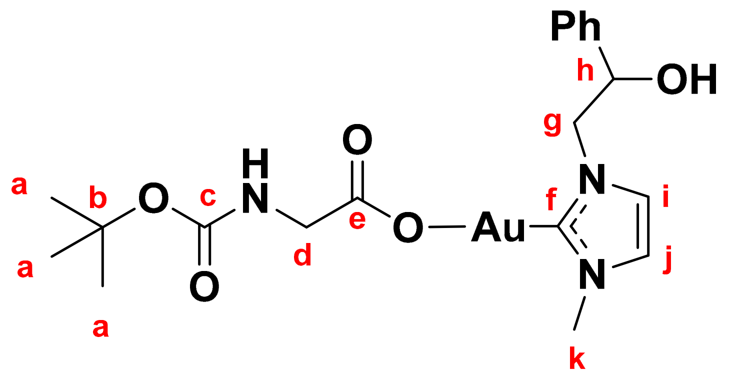

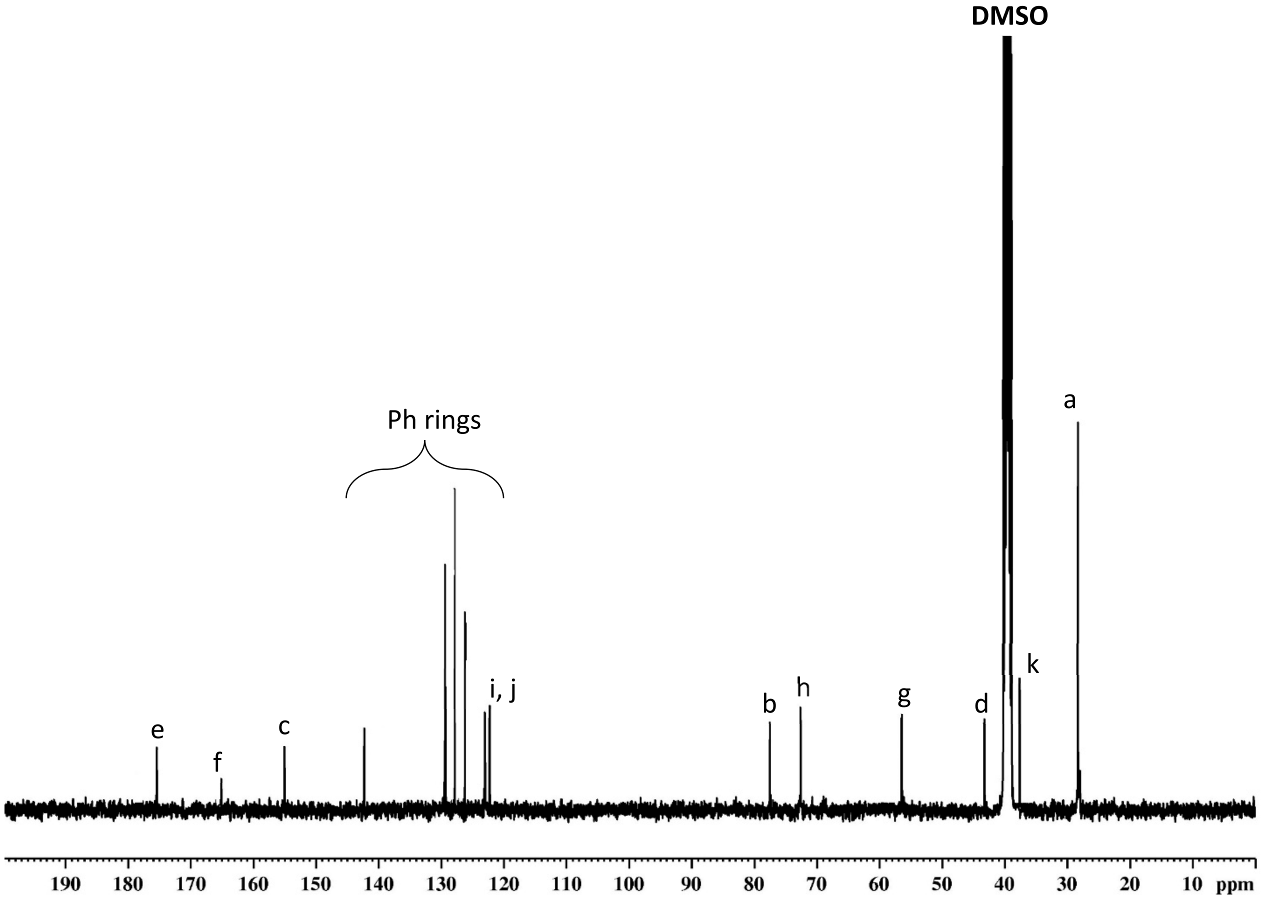

^{13}C-NMR (100 MHz, DMSO-d$_6$, ppm): δ 175.4 (**C**OOAu); 164.5 (N**C**N); 155.3 (NH**C**O);, 142.5; 129.9, 127.6,126.5 (**Ph** ring); 123.1,122.5 (N**C**H**C**HN); 77.4 (**C**(CH$_3$)$_3$); 72.7 (**C**HOH); 56.3 (N**C**H$_2$); 43.6 (NH**C**H$_2$); 37.2 (N**C**H$_3$); 28.5 (C(**C**H$_3$)$_3$).

MALDI AuL20Gly

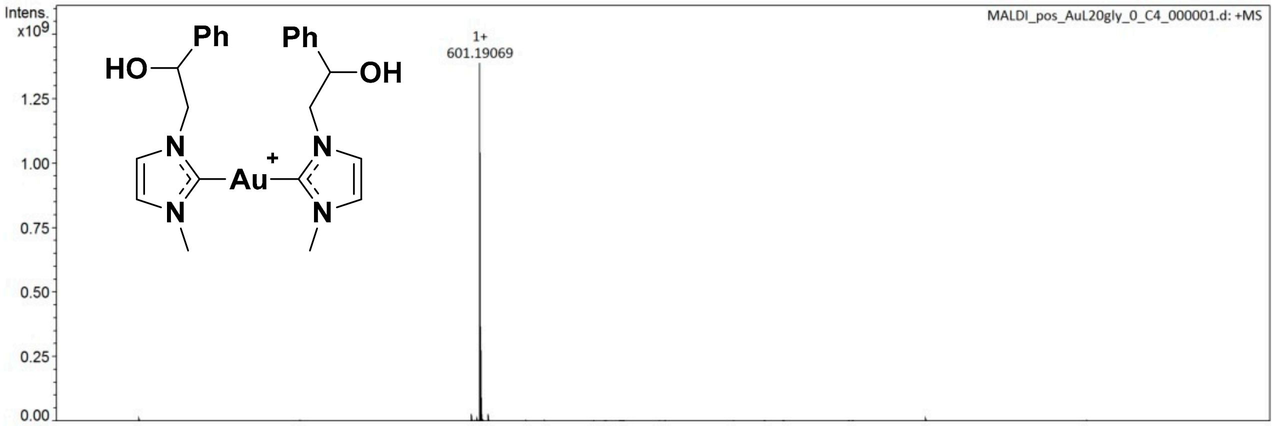

MALDI-MS (m/z): 601.19069 attributable to bis-carbene structure $[C_{24}H_{28}AuN_4O_2]^+$

Elemental Analysis: theorical = C: 39.80, H 4.57, Au 34.35, N 7.33, O 13.95; experimental= C 39.75, H 4.82, N 7.30

^{1}H-NMR AuL20Phe

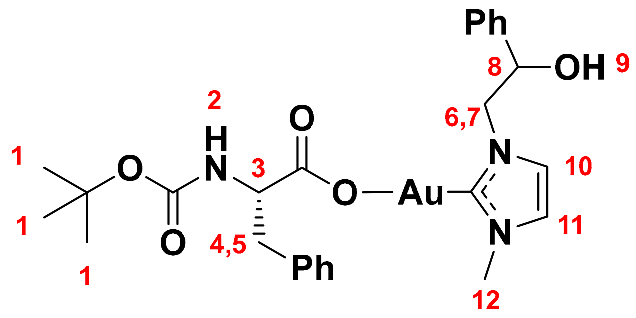

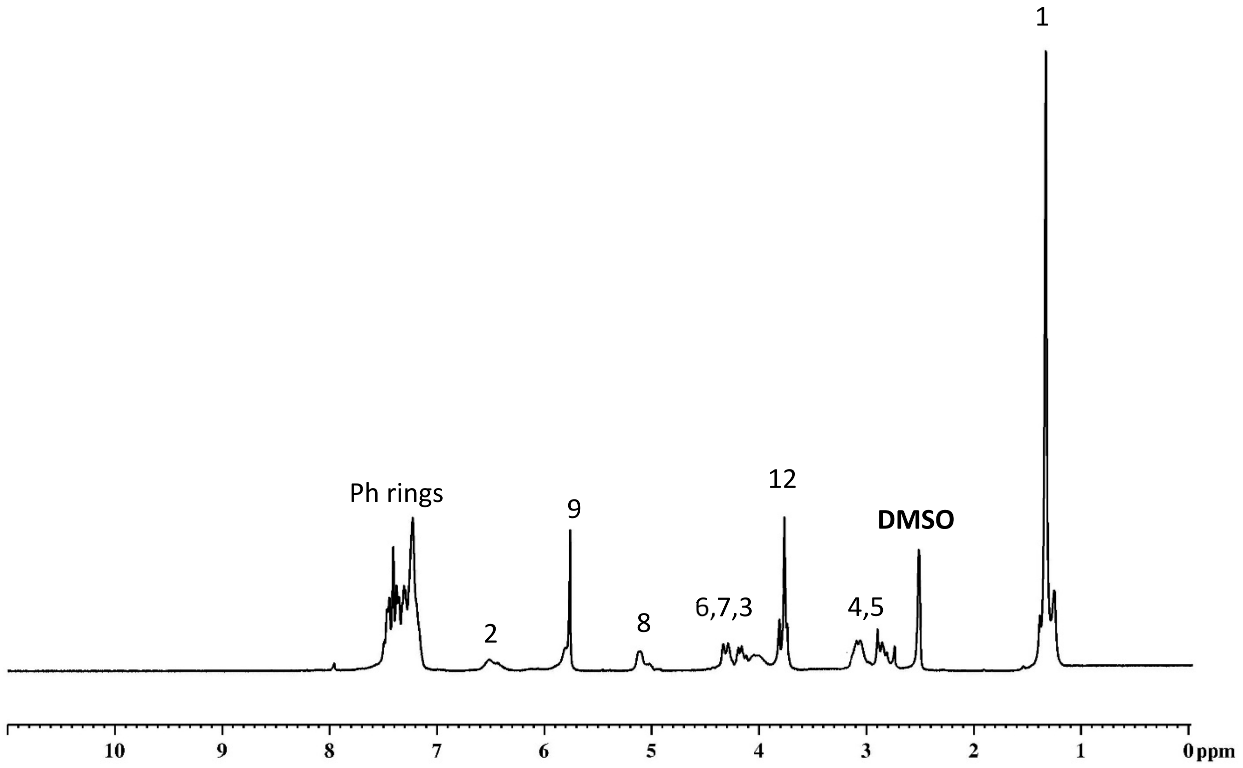

^{1}H-NMR (300 MHz, DMSO-d_6, ppm): δ 7.40 - 7.17 (m, 12 H, $C_6\mathbf{H}_5CH(OH)CH_2NC\mathbf{H}C\mathbf{H}N$+Phe:$C_6\mathbf{H}_5$); 6.20 (br, 1H, Phe: N**H**); 5.77 (d, 1H, $C_6H_5CH(O\mathbf{H})CH_2N$); 5.10 (br, 1H, $C_6H_5C\mathbf{H}(OH)CH_2N$); 4.26-4.16 (dd, J_{gem}=14.6 Hz, J_{vic}=10.6 Hz, 8.9 Hz, 2H, $C_6H_5CH(OH)C\mathbf{H}_2N$); 4.01 (br, 1H, Phe: $C\mathbf{H}CH_2Ph$); 3.74 (s, 3H, $NC\mathbf{H}_3$); 3.09-2.88 (dd, J_{gem}=14.0 Hz, J_{vic}=9.3 Hz, 8.7 Hz, 2H, Phe: $CHC\mathbf{H}_2Ph$); 1.39 (s, 9H, Phe: $C(C\mathbf{H}_3)_3$).

^{13}C-NMR AuL20Phe

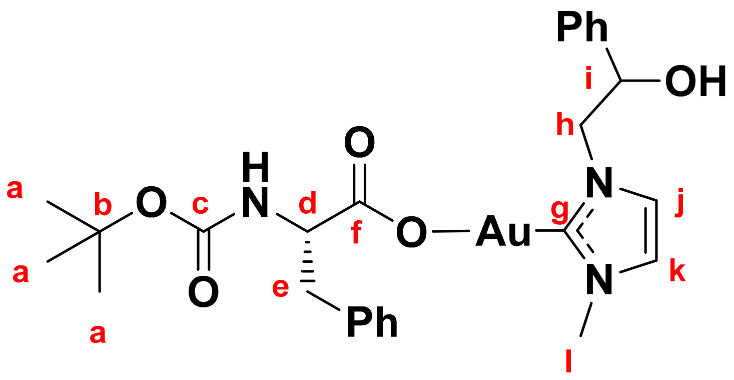

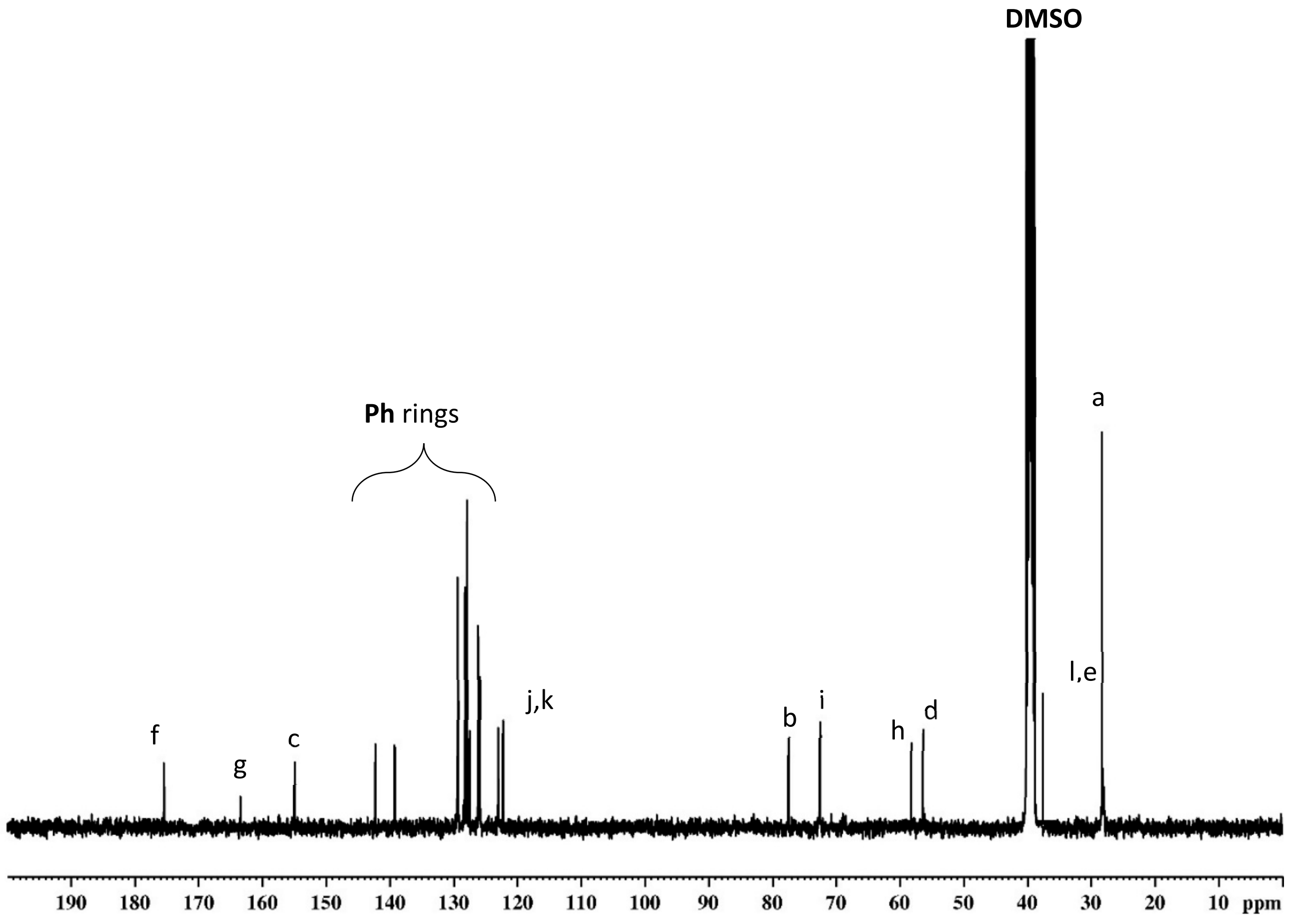

^{13}C-NMR (100 MHz, DMSO-d$_6$, ppm): δ 175.4 (**C**OOAu); 164.4 (N**C**N); 154.9 (NH**C**O); 142.2; 139.2, 129.3, 128.2, 127.8, 127.5, 126.1, 125.8 (**Ph** rings); 124.2, 122.9 (N**C**H**C**HN); 77.4 (**C**(CH$_3$)$_3$); 72.5 (**C**HOH); 58.2 (N**C**H$_2$); 56.4 (NH**C**H); 38.1 (N**C**H$_3$); 37.9 (Ph**C**H$_2$); 28.3(C(**C**H$_3$)$_3$).

MALDI AuL20Phe

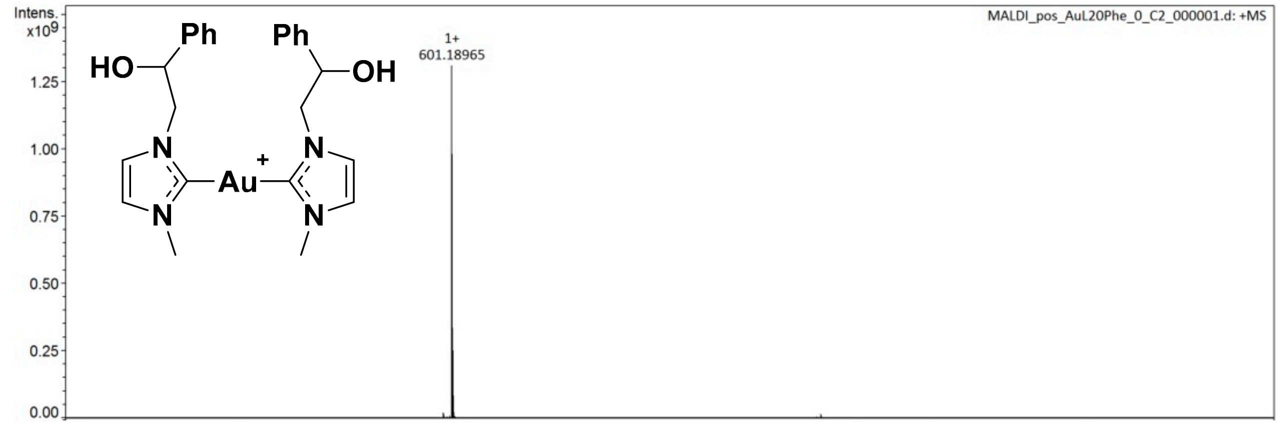

MALDI-MS (m/z): 601.18965 attributable to a bis-carbene structure $[C_{24}H_{28}AuN_4O_2]^+$

Elemental Analysis: theorical = C: 47.06, H 4.86, Au 29.68, N 6.33, O 12.07; experimental= C 47.05, H 4.86, N 6.40

^{1}H-NMR AuM1Gly

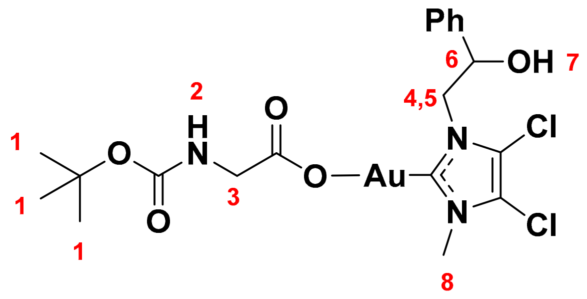

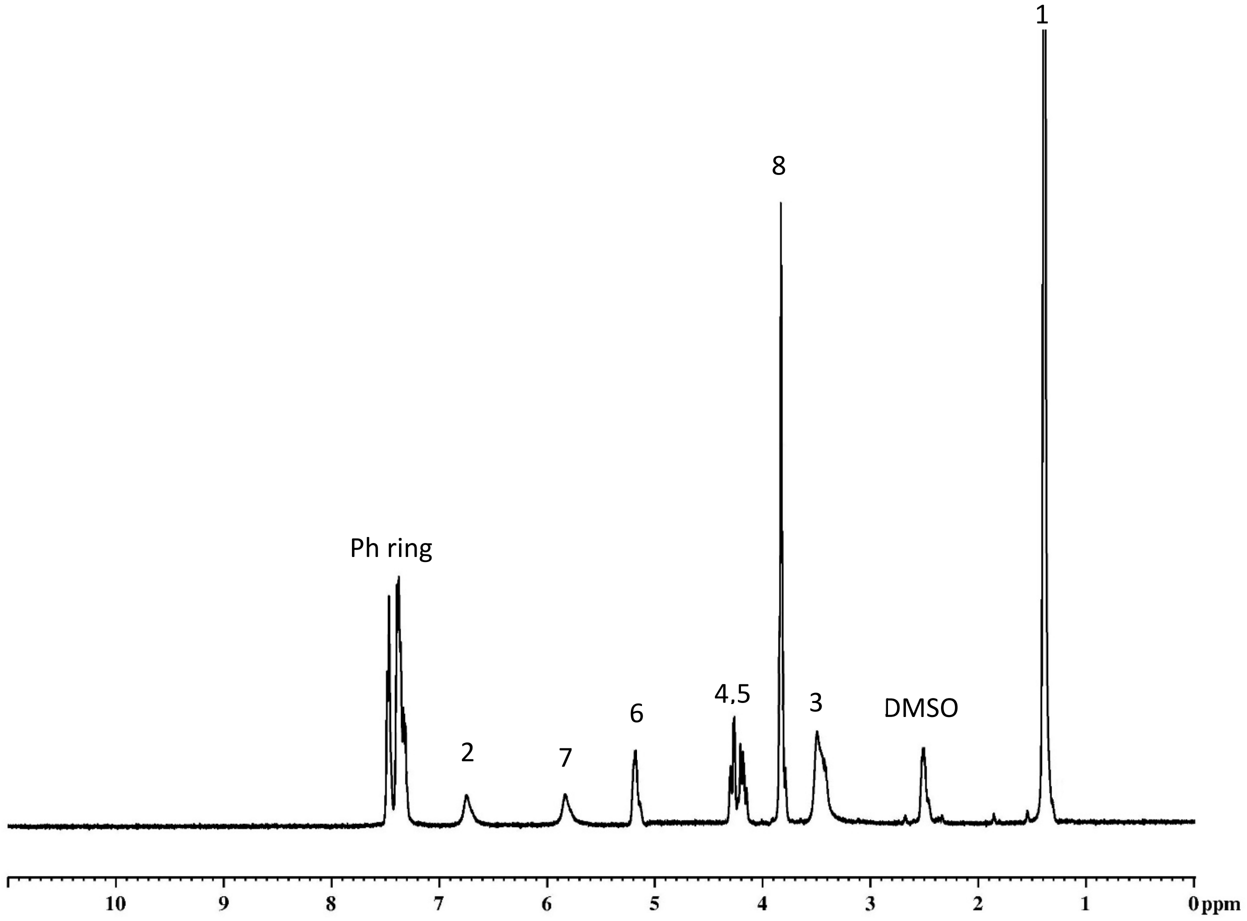

^{1}H-NMR (300 MHz, DMSO-d_6, ppm): δ 7.37-7.35 (m, 5H, $C_6\mathbf{H}_5CH(OH)CH_2N$); 6.84 (br, 1H, Gly: N**H**); 5.97 (br, 1H; $C_6H_5CH(O\mathbf{H})CH_2N$); 5.20 (m, J_{vic}= 10.7 Hz, 8.6 Hz, 1H, $C_6H_5C\mathbf{H}(OH)CH_2N$); 4.25 (m, J_{gem}=13.9 Hz, J_{vic}= 10.7 Hz, 8. 6Hz, 2H, $C_6H_5CH(OH)C\mathbf{H}_2N$); 3.81 (s, 3H, $NC\mathbf{H}_3$); 3.25 (s, 2H, Gly: $NHC\mathbf{H}_2$); 1.36 (s, 9H, Gly: $C(C\mathbf{H}_3)_3$).

^{13}C-NMR AuM1Gly

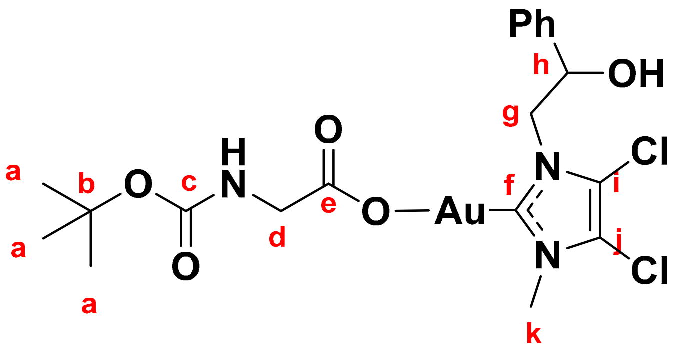

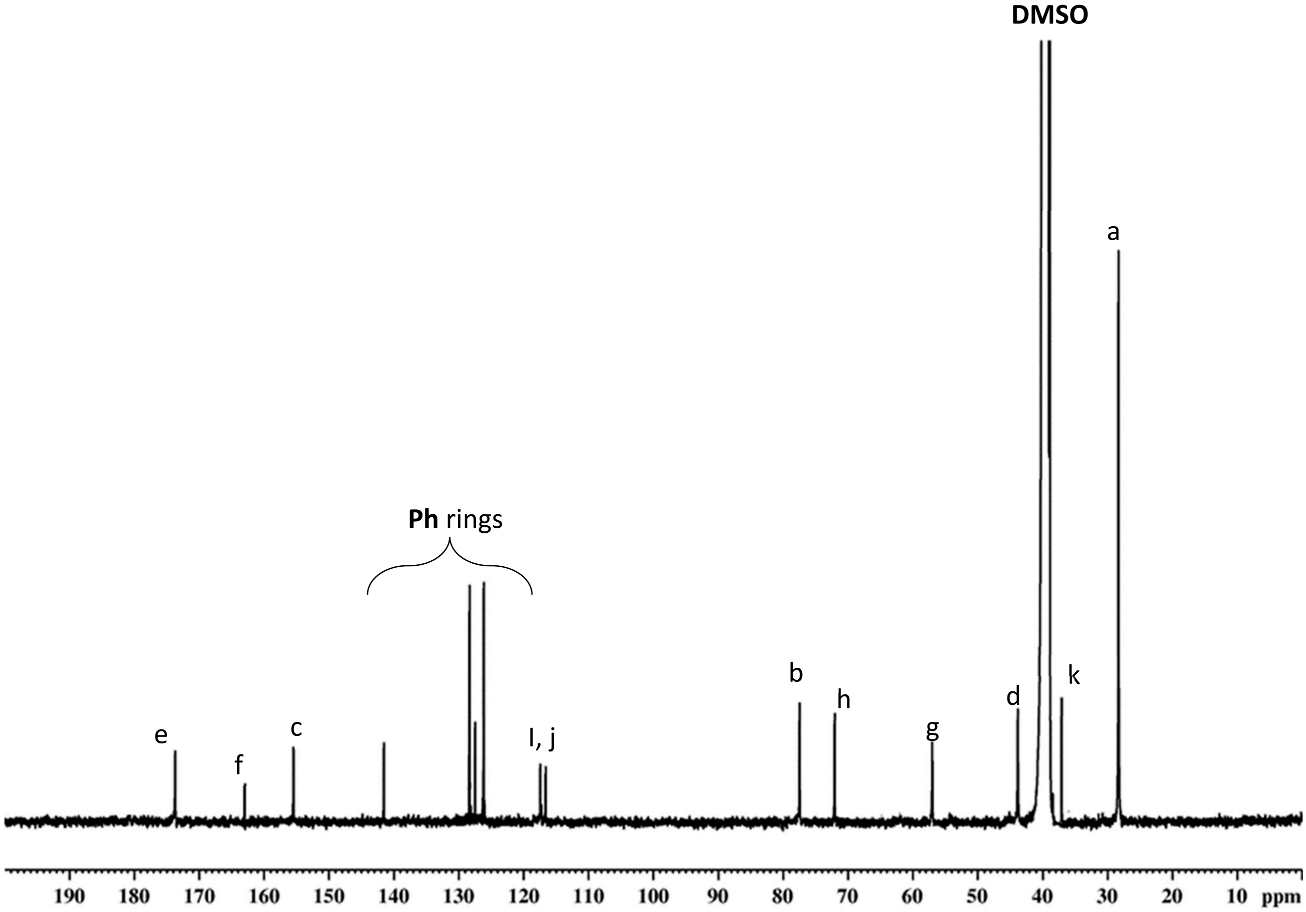

^{13}C-NMR (75 MHz, DMSO-d$_6$, ppm): δ 173.7 (**C**OOAu); 163.2 (N**C**N); 155.5 (NH**C**O); 141.2, 128.8,127.7, 125.1 (**Ph** rings); 117.4, 116.5 (N**C**Cl**C**ClN); 77.5 (**C**(CH$_3$)$_3$); 72.1 (**C**HOH); 56.6 (N**C**H$_2$); 44.1 (NH**C**H$_2$); 37.2 (N**C**H$_3$); 28.3 (C(**C**H$_3$)$_3$).

MALDI AuM1Gly

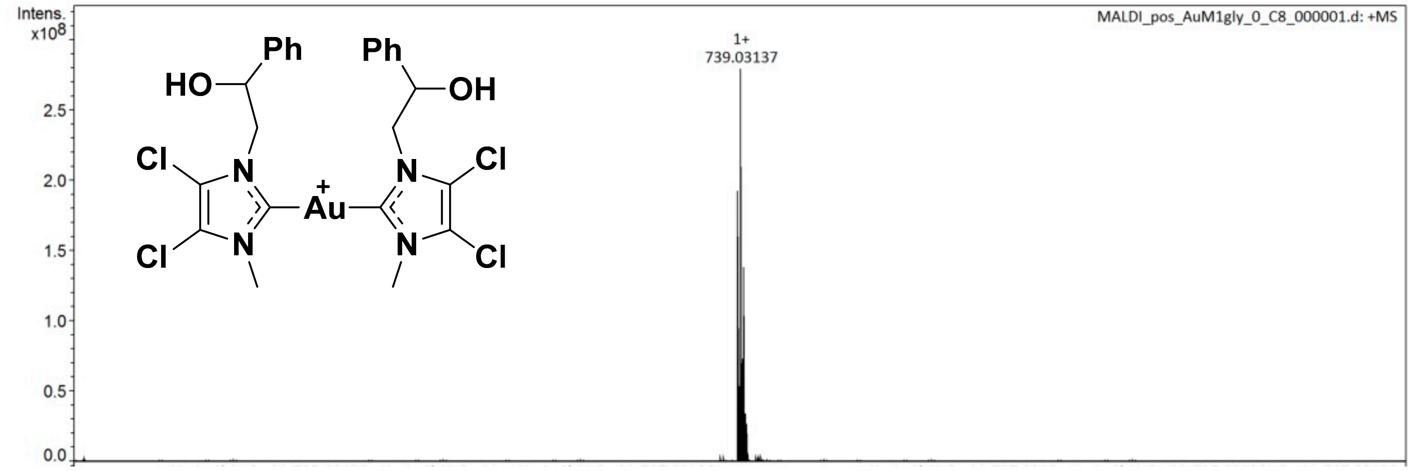

MALDI-MS (m/z): 739.03137 attributable to bis-carbene structure $[C_{24}H_{24}AuCl_4N_4O_2]^+$

Elemental Analysis: theorical = C: 35.53, H 3.77, Au 30.67, Cl 11.04, N 6.54, O 12.45; experimental= C 35.57, H 3.80, Cl 11.10, N 6.58.

^{1}H-NMR AuM1Phe

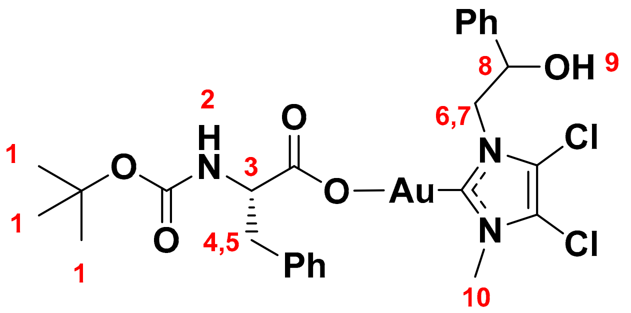

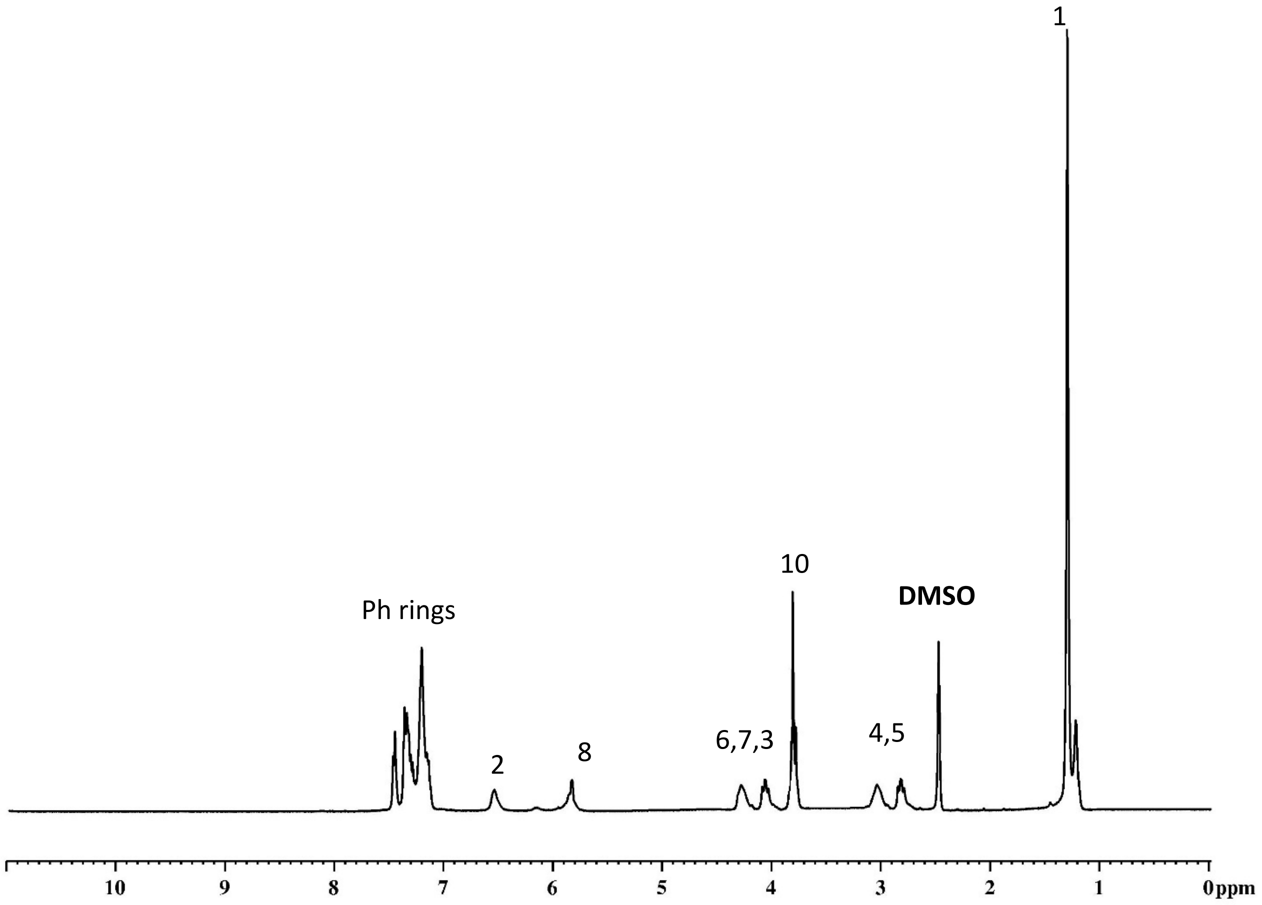

^{1}H-NMR (400 MHz, DMSO-d_6, ppm): δ 7.43-7.20 (m, 10H, $C_6\mathbf{H}_5CH(OH)CH_2N$+Phe:$C_6\mathbf{H}_5$); 6.30 (br, 1H, Phe: N**H**); 5.84 (br, 1H, $C_6H_5CH(O\mathbf{H})CH_2N$); 4.27 (m, 2H, $C_6H_5CH(OH)C\mathbf{H}_2N$); 4.04 (m, J_{vic}= 8.6 Hz, 7.8 Hz, 6.8 Hz, 1H, Phe: C**H**CH_2Ph); 3.80 (s, 3H, $NC\mathbf{H}_3$); 3.11-2.87 (dd, J_{gem}=14.8 Hz, J_{vic}= 8.6 Hz, 7.8 Hz, 1H, Phe: C**H**CH_2Ph); 1.31 (s, 9H, Phe: $C(C\mathbf{H}_3)_3$).

^{13}C-NMR AuM1Phe

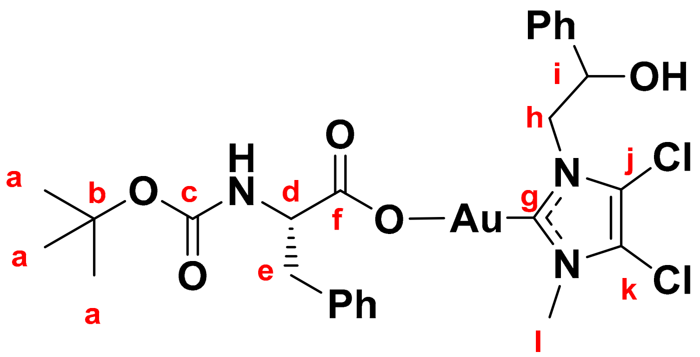

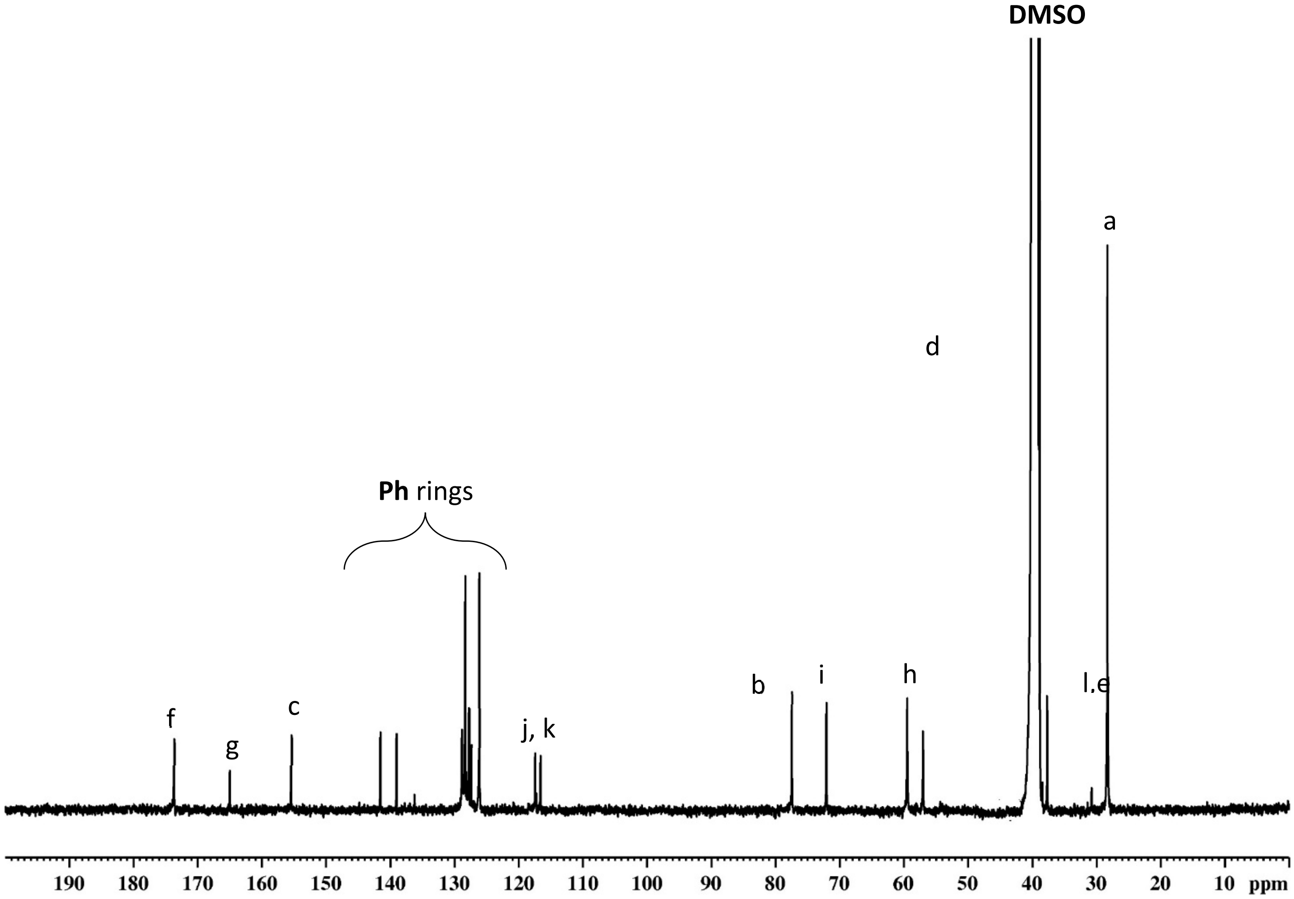

^{13}C-NMR (75 MHz, DMSO-d$_6$, ppm): δ 175.0 (**C**OOAu); 163.3 (N**C**N); 155.1 (NH**C**O); 141.3, 138.8, 129.2,128.2, 127.9, 125.9 (**Ph** rings); 117.5, 116.5 (N**C**Cl**C**ClN); 77.6 (**C**(CH$_3$)$_3$);; 72.1 (**C**HOH); 56.6 (N**C**H$_2$); 52.0 (**C**HNH); 37.2 (N**C**H$_3$); 37.0 (**C**H$_2$Ph); 28.2 (C(**C**H$_3$)$_3$).

MALDI AuM1Phe

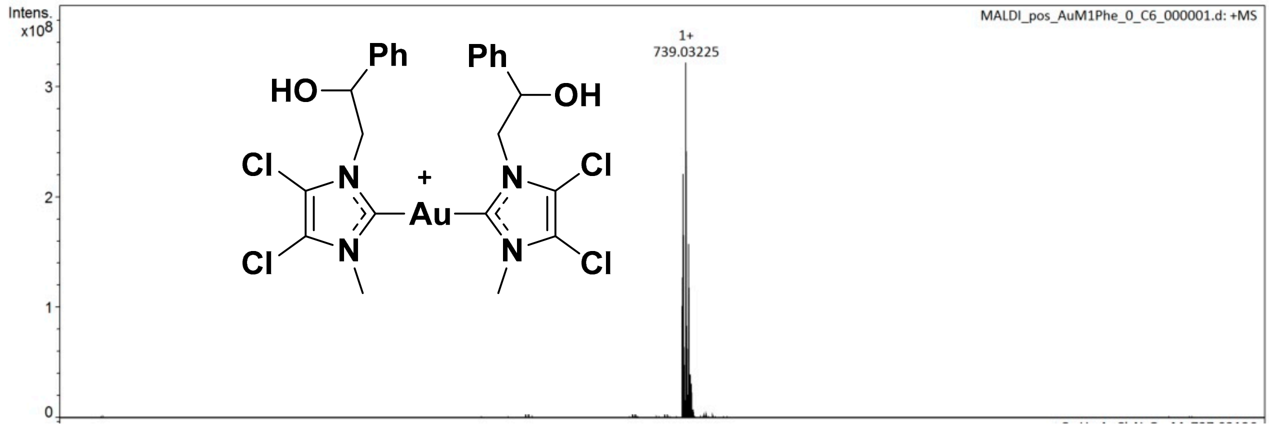

MALDI-MS (m/z): 739.03225 attributable to bis-carbene structure $[C_{24}H_{24}AuCl_4N_4O_2]^+$

Elemental Analysis: theorical = C: 41.86, H 3.79, Au 27.46, Cl 9.88, N 5.86, O 11.15; experimental= C 41.90, H 3.80, Cl 9.87, N 5.90.

FT-IR Characterization

Fourier transform infrared (FT-IR) spectra were obtained at a resolution of 2.0 cm^{-1} with a Bruker-Vector 22 FT-IR spectrometer equipped with a deuterated triglycine sulfate (DTGS) detector and Ge/KBr beam splitter. The frequency scale was internally calibrated to 0.01 cm^{-1} using a HeNe reference laser. Thirty-two scans were signal averaged to reduce spectral noise. The spectra were performed in KBr disks in a frequency range between 4000 and 450 cm^{-1}.

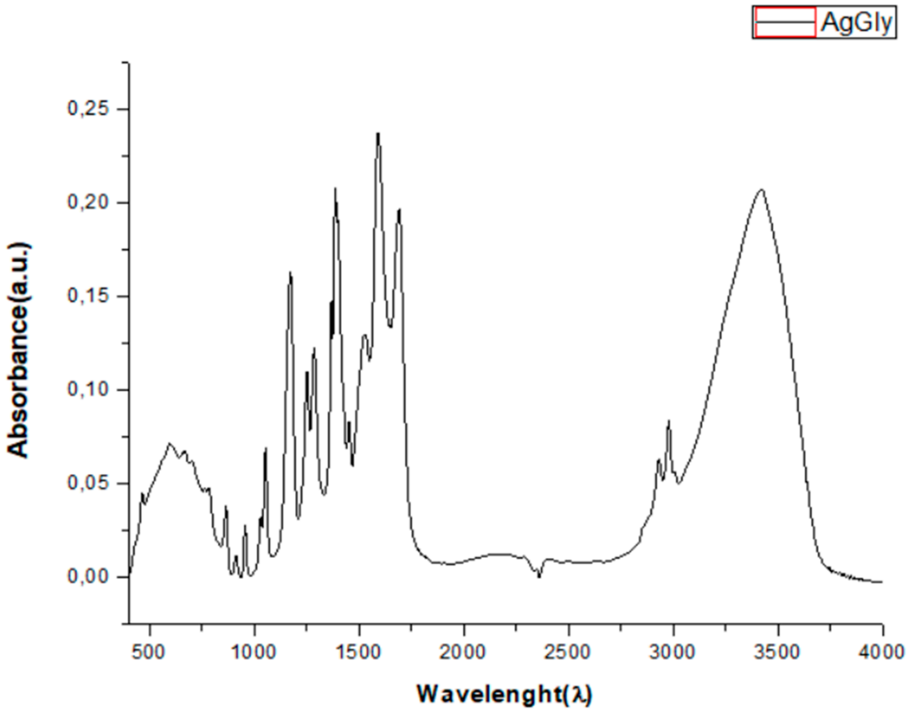

IR (KBr, disk, cm^{-1}): ν 1691 (C=O); 1591 (C=O amide).

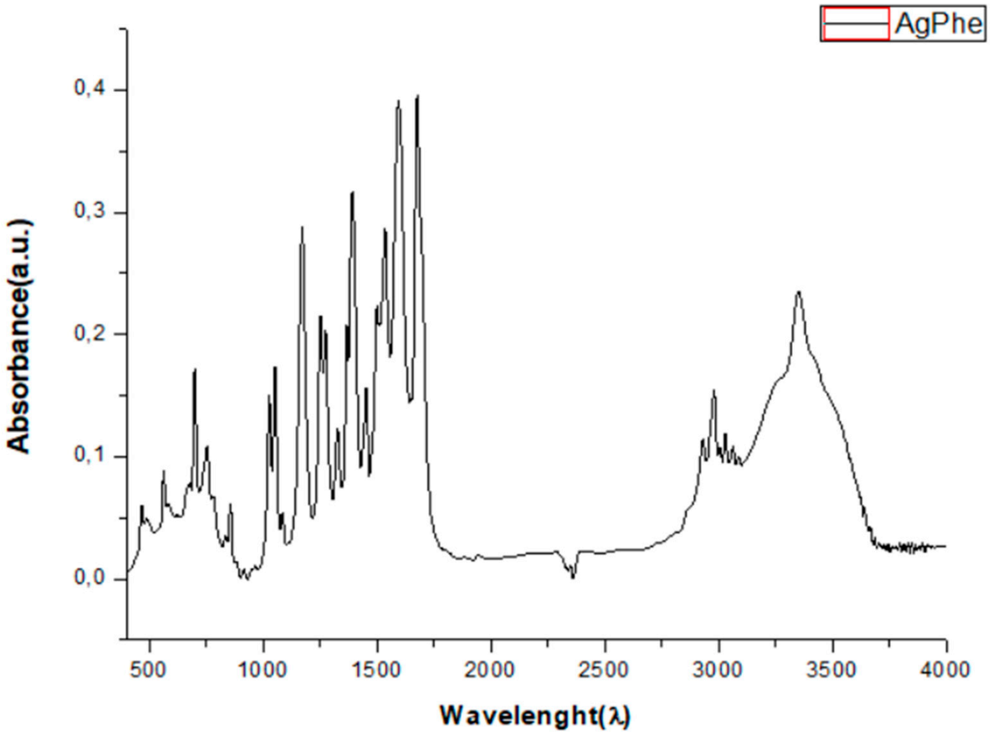

IR (KBr, disk, cm⁻¹): ν 1676 (C=O); 1594 (C=O $_{amide}$); 1535 (C=C $_{Ph\ ring}$).

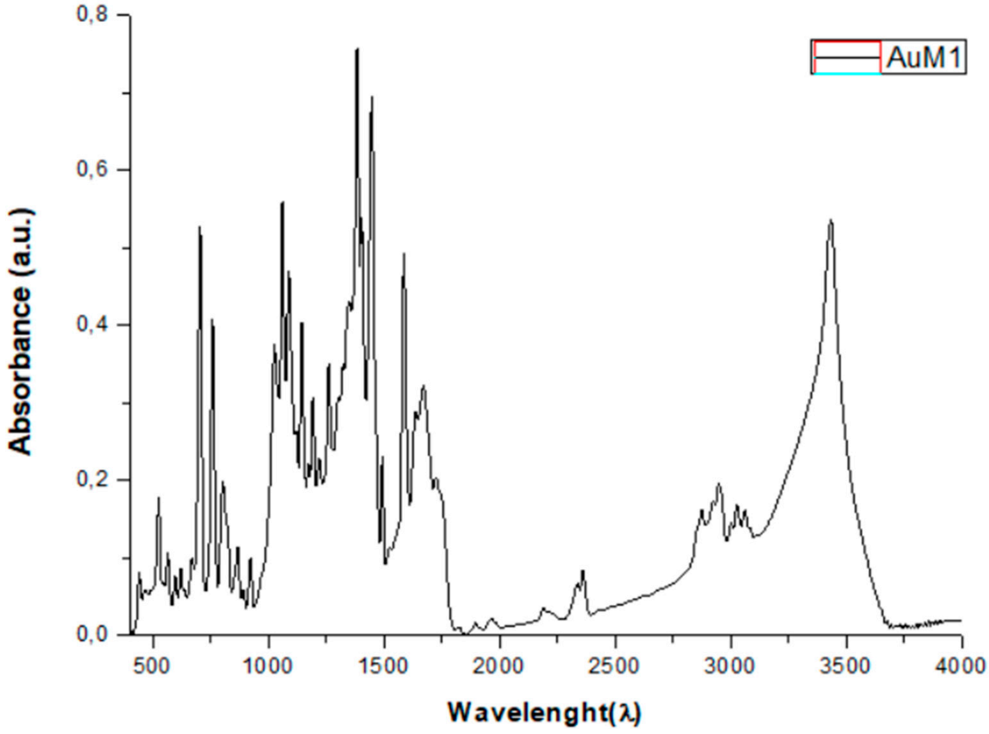

IR (KBr, disk, cm⁻¹): ν 1586 (C=C $_{Ph\ ring}$).

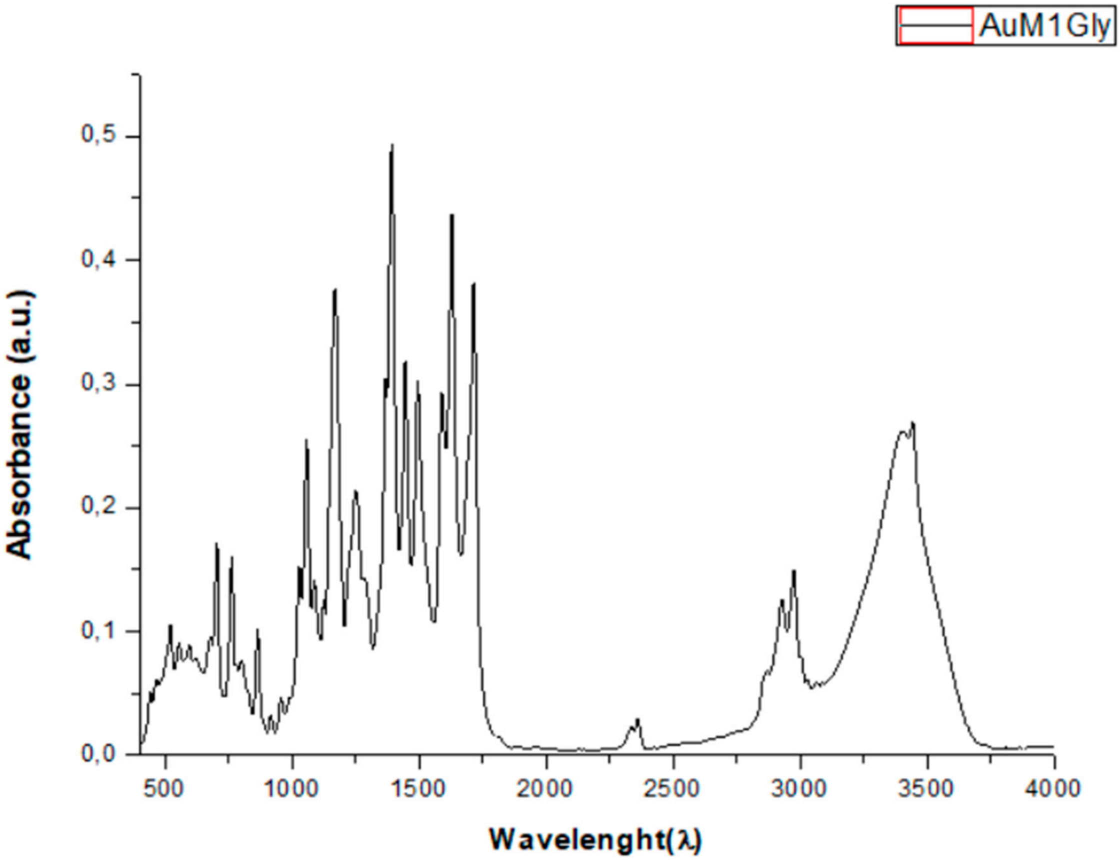

IR (KBr, disk, cm^{-1}): ν 1715 (C=O); 1629 (C=O $_{amide}$); 1589 (C=C $_{Ph\ ring}$).

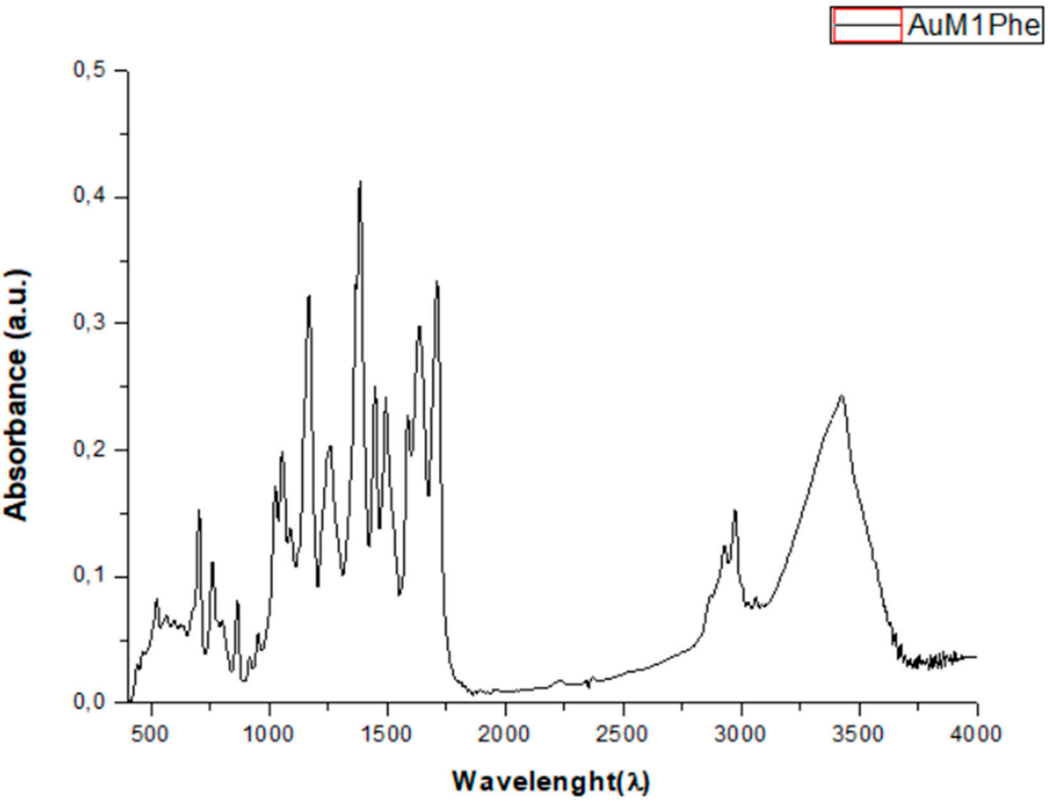

IR (KBr, disk, cm^{-1}): ν 1712 (C=O); 1636 (C=O $_{amide}$); 1588 (C=C $_{Ph\ ring}$).

Thermal stability of AgM1Gly

Thermal stability of AgM1Gly was determined. Thermogravimetric analysis (TGA) of this complex, reported in **Figure S1,** shows that its decomposition temperature is around 150 °C. This finding was further confirmed by differential scanning calorimetry (DSC) analysis of the sample, see **Figure S2**. Indeed, the thermogram gave an endothermic peak of around 150 °C, probably due to the heat required for the decomposition of the sample. This peak is not observed in a second heating run of the same sample.

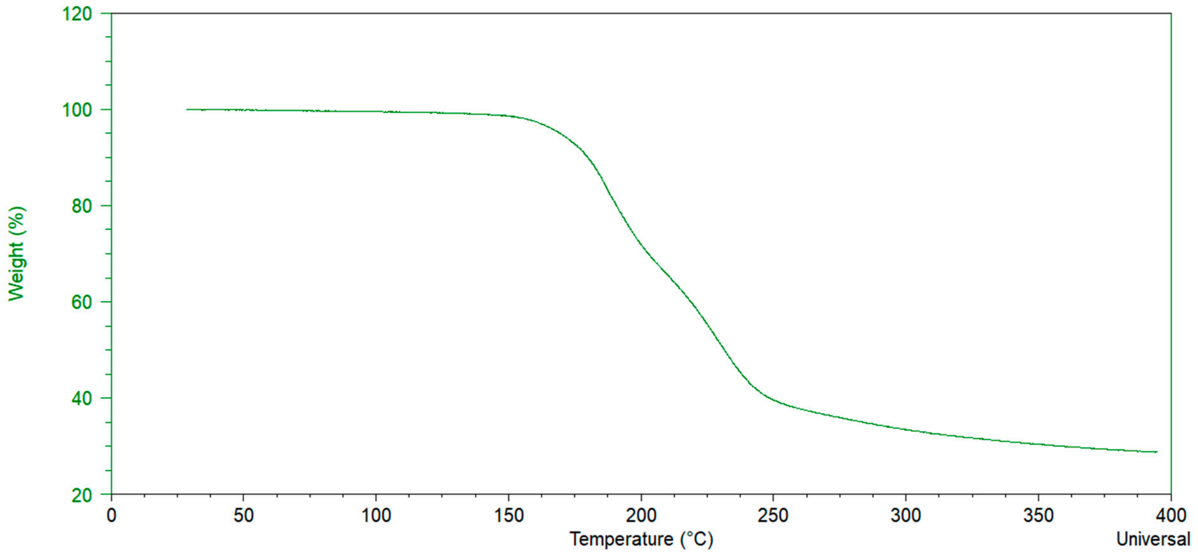

Figure S1. Thermogravimetric analysis of AgM1Gly.

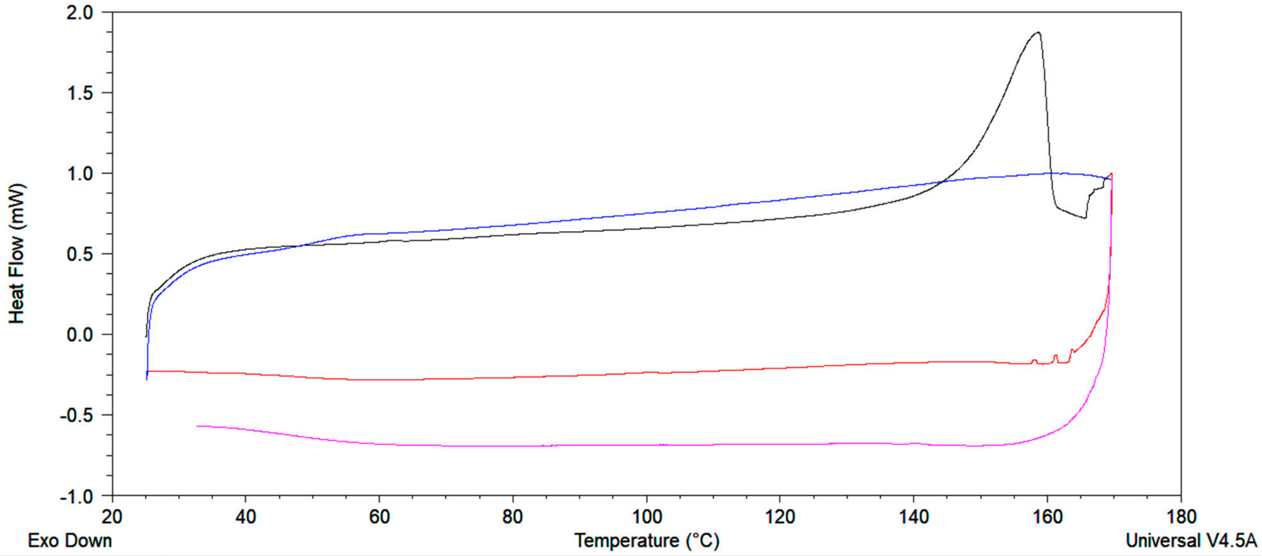

Figure S2. Differential Scanning Analysis of AgM1Gly

Hydrolytic Stability of AgM1Gly, AgPhe, AuM1 and AuM1Phe

The hydrolytic stability of AgM1Gly, AgPhe, AuM1 and AuM1Phe was determined in D_2O-10% of DMSO-d_6, following the procedure in ref. [23]. **Figures S3–S6** show the ^{1}H-NMR spectra of the complexes at time = 0h, 2h, 4h, 24h, 48h and 72h, no changes are observed in time. These preliminary studies can be reasonably extended to all synthesized complexes because they have a similar structure. Thus, it is possible to affirm that these complexes are hydrolytically stable.

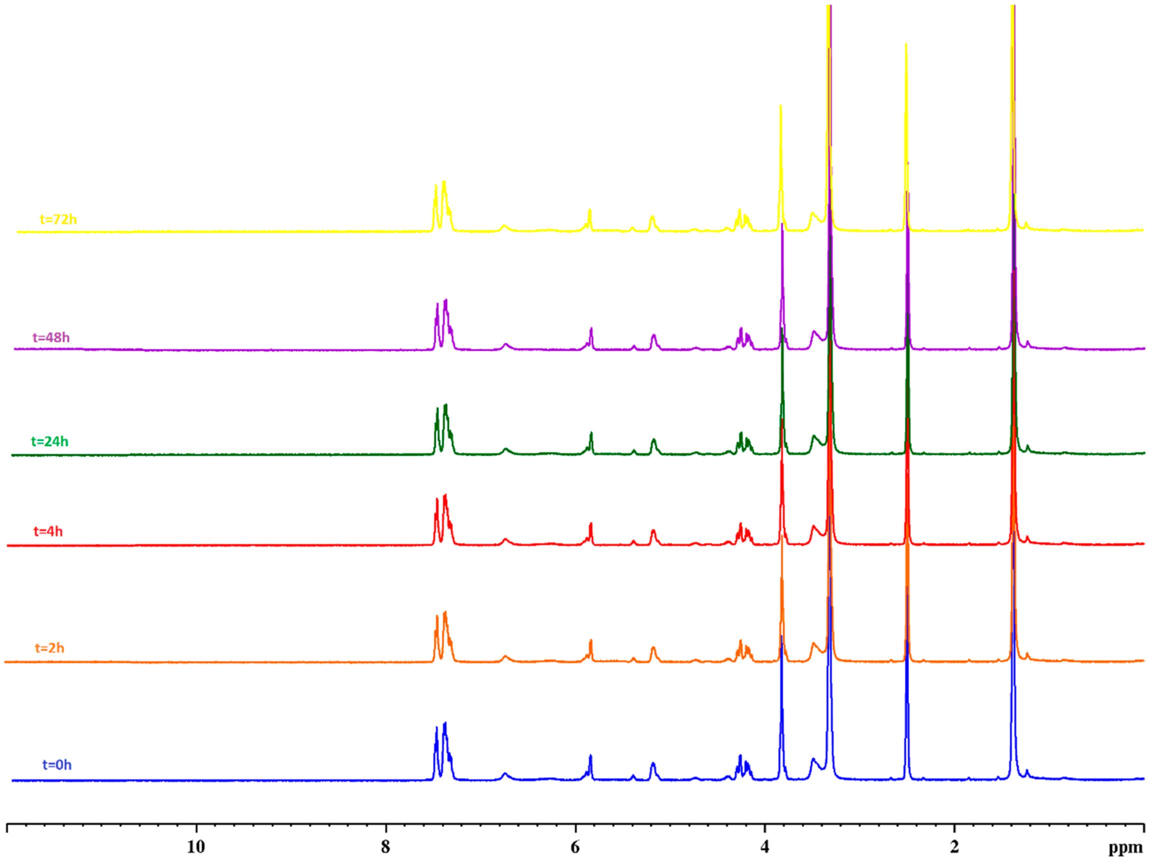

Figure S3. Hydrolytic Stability of the complex AgM1Gly.

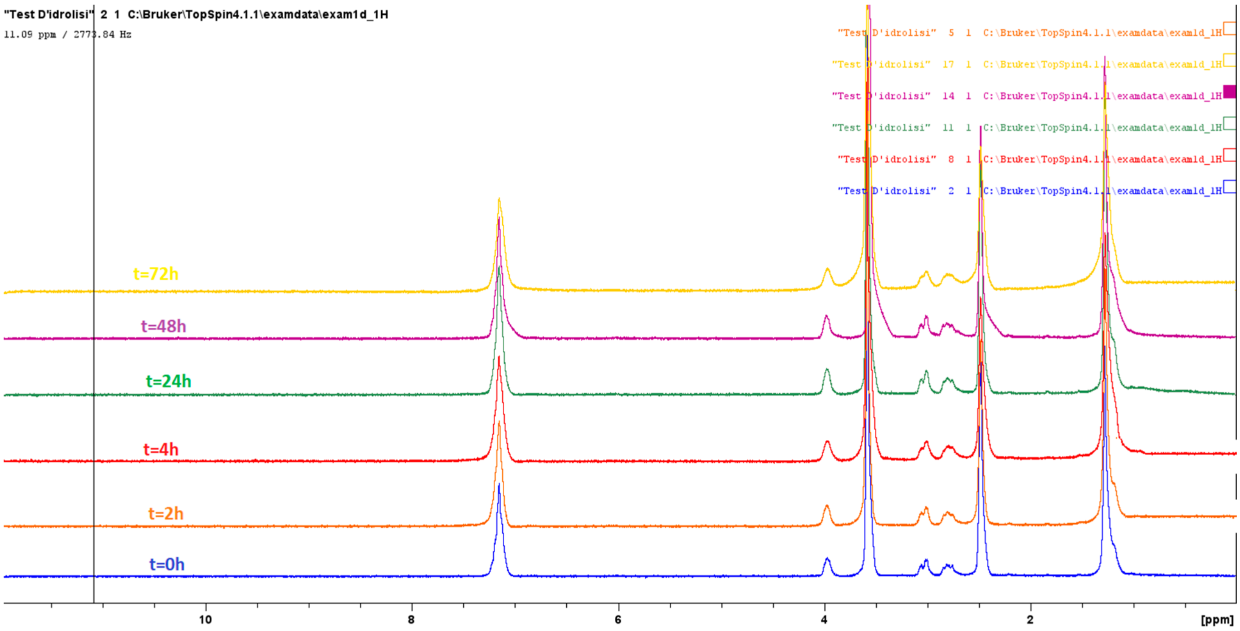

Figure S4 Hydrolytic Stability of the complex AgPhe.

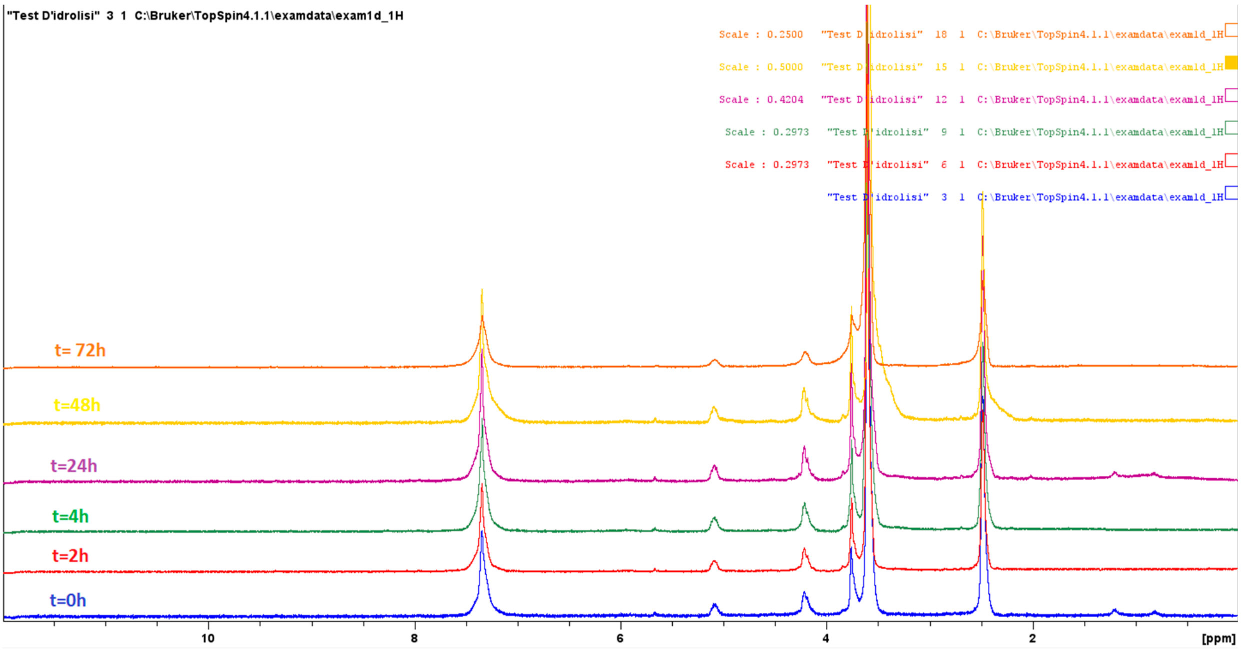

Figure S5 Hydrolytic Stability of the complex AuM1

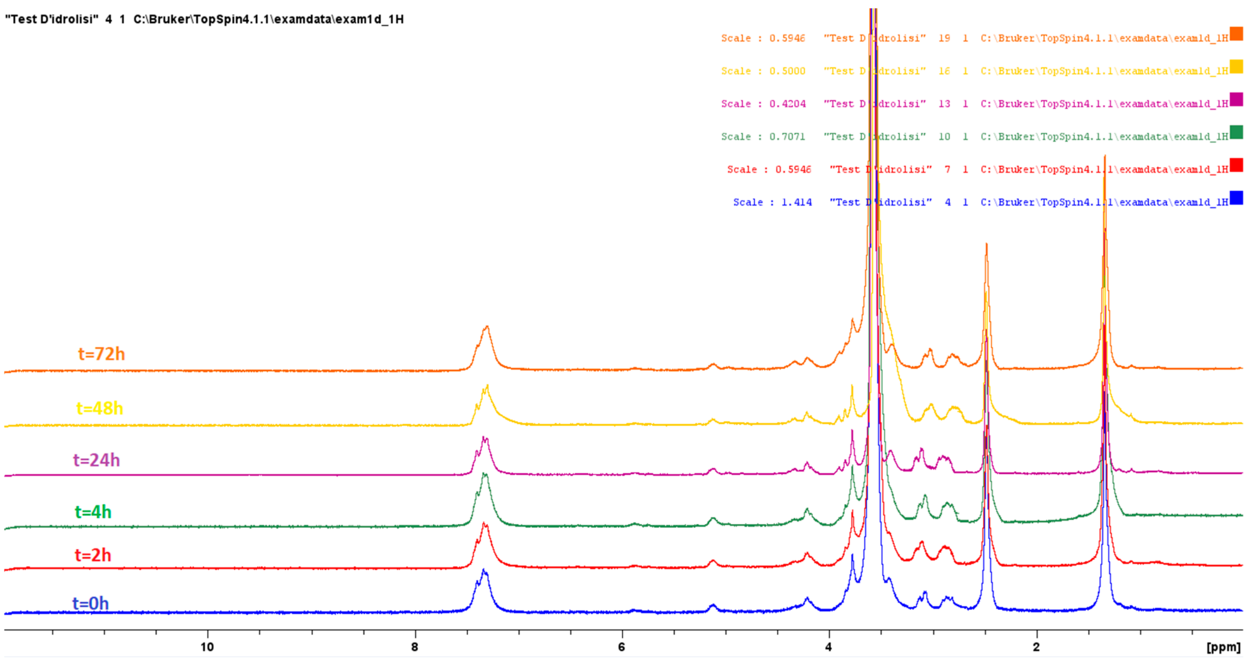

Figure S6 Hydrolytic Stability of the complex AuM1Phe

IC50 values

For the four main complexes (AuM1, AuL20, AgM1 and AgL20) at 20 μM after 24h and 48h of treatment were calculated (see also Table 2 in the manuscript). The related plots were reported in **Figure S7**.

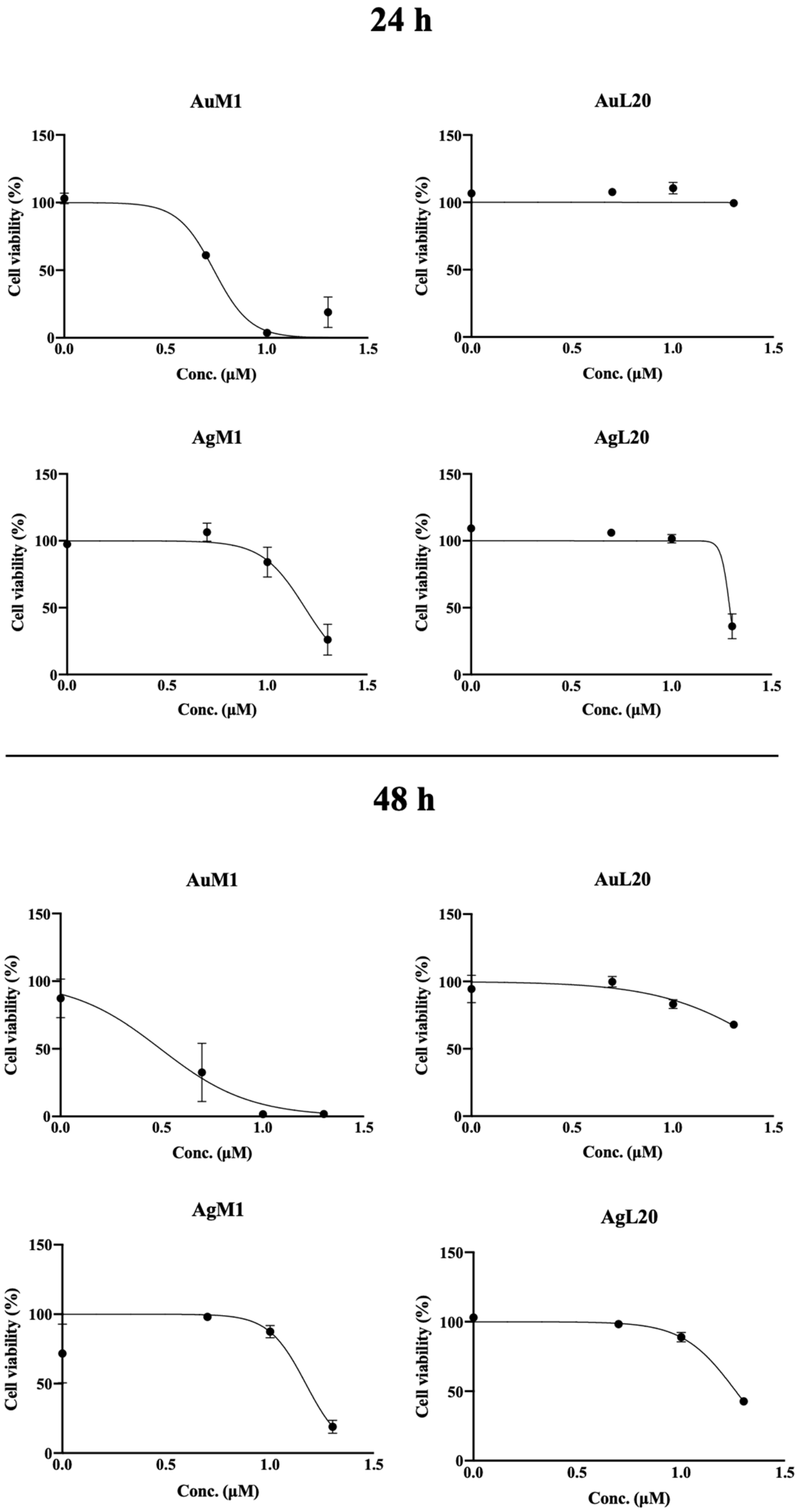

Figure S7 IC50 plots for AuM1, AuL20, AgM1 and AgL20.
